# One Dimensional Reduction of a Renewal Equation for a Measure-Valued Function of Time Describing Population Dynamics

**DOI:** 10.1007/s10440-021-00440-3

**Published:** 2021-10-06

**Authors:** Eugenia Franco, Mats Gyllenberg, Odo Diekmann

**Affiliations:** 1 Department of Mathematics and Statistics, University of Helsinki, Helsinki, Finland; 2 Mathematical Institute, Utrecht University, Utrecht, Netherlands

**Keywords:** Malthusian parameter, Balanced exponential growth, Volterra integral equations, Laplace transform, Convolution

## Abstract

Despite their relevance in mathematical biology, there are, as yet, few general results about the asymptotic behaviour of measure valued solutions of renewal equations on the basis of assumptions concerning the kernel. We characterise, via their kernels, a class of renewal equations whose measure-valued solution can be expressed in terms of the solution of a scalar renewal equation. The asymptotic behaviour of the solution of the scalar renewal equation, is studied via Feller’s classical renewal theorem and, from it, the large time behaviour of the solution of the original renewal equation is derived.

## Introduction

Renewal equations are a class of integral equations which, in their simplest form, look as

1.1 $$ b(t) = \int _{0}^{\infty }b(t-a) k(a) da \quad \text{ for } t >0. $$

The solution $b$ of a renewal equation evaluated at a certain time $t$ depends on its history, that is, on all its values up to time $t$. The dependence on the history is filtered by the kernel $k$, which weighs each point in the past. To obtain a unique solution one needs to prescribe $b(t)$ on $(-\infty ,0]$.

Consider a population of individuals, characterised by their age and concomitant reproducing ability. Reproductive individuals contribute to the population birth rate according to the reproduction capacity of their age. The density of individuals of age $a$ depends only on the population birth rate $a$ time units ago and the probability of surviving up to age $a$. We therefore infer that the population birth rate satisfies the renewal equation (1.1) with an appropriately chosen kernel. Similar considerations lead to the use of more general renewal equations (cf. (1.2) below) in a broad variety of structured population models. We find applications in demography [40], epidemiology [22, 39, 52], models of the immune system [17], and models of populations of animals and cells [21, 36, 41, 42, 50].

In this work we are interested in a class of linear structured population models, including a model of cell growth and division and a model of the waning and boosting of immunity, see Sect. 4. The populations that we study consist of individuals characterised by their *individual state*, or i-state for short. The set of all admissible i-states is called the i-state space and is denoted by $\Omega $. In all our applications it will be a nonempty subset of ${\mathbb{R}}^{n}$. The subset $\Omega _{0} \subset \Omega $ is the set of all possible states at birth, which in most models is considerably smaller than the full i-state space $\Omega $, but might have the cardinality of the continuum. The motivation of the subscript 0 in $\Omega _{0}$ is that $\Omega _{0}$ is the set of the states of individuals of age 0. We shall frequently abuse language and talk about individuals when we really mean i-states, for instance, in phrases like “individuals in the set $\omega \subset \Omega $”.

The i-level mechanisms that we model are

- Reproduction. Here we have to specify how much offspring an individual with a particular i-state produces per unit of time and what the states at birth of the offspring are. In models where an individual may change its i-state through a jump, it is often convenient to consider this jump as part of the reproduction process: the reappearance of an individual at a different location in the i-state space is modelled as the birth of a new individual.
- Development of the i-states, for example growth of a cell or waning of the immune level.
- Disappearance of individuals, by death or by a jump to elsewhere in the i-state space.

For general structured population models, we need to incorporate the state-at-birth in the bookkeeping. In [18] this was elaborated in terms of measures on the product of the time axis and $\Omega _{0}$. Here we focus on situations in which individuals give birth at a certain rate, i.e., with a certain probability per unit of time. In such situations it is natural to work with rates at the population level as well, as in (1.1). But in the state-at-birth variable we retain the measure character, as

(i) it allows us to treat the cases of finitely many birth states and a continuum of birth states in a unified manner
(ii) it prepares for a description of the population state by a measure on $\Omega $ (which is very natural: the number of individuals with i-state belonging to a certain subset of $\Omega $ is conceptually easier than a density on $\Omega $).

So the key variable is $b(t,\omega )$ such that $\int _{[t_{1},t_{2}]} b(t,\omega ) dt $ is the number of individuals born in the time interval $[t_{1},t_{2}]$ with state-at-birth in the set $\omega \subset \Omega _{0}$.

To formulate the renewal equation for measure valued functions $b$ of time, we introduce a kernel, i.e., a function $k: {\mathbb{R}}_{+} \times \Omega _{0} \times \mathscr {B}(\Omega _{0}) \to {\mathbb{R}}_{+}$ with certain natural properties as specified in Definition A.8 in the Appendix A, and write

1.2 $$ b(t, \omega )=\int _{0}^{\infty }\int _{\Omega _{0}} b(t-a, d\xi ) k(a, \xi , \omega ) da \quad \text{ for } t >0, \quad \omega \in \mathscr {B}( \Omega _{0}) . $$

A solution of (1.2) is a function $t \mapsto b(t, \cdot )$ from $\mathbb{R}_{+}^{*}$ to $M(\Omega _{0})$, satisfying (1.2) for all $\omega \in {\mathscr {B}}(\Omega _{0})$. The biological interpretation is that $b(t,\omega )$ is the rate at which individuals are born with state at birth in $\omega \in {\mathscr {B}}(\Omega _{0})$ at time $t$, and that $k(a,\xi ,\omega )$ is the rate at which an individual, which had i-state $\xi $ at birth, is expected to give birth to offspring with states at birth in $\omega $, when it is of age $a$. So an individual that dies before reaching age $a$ contributes zero to the expected value.

Models of physiologically structured populations are often formulated in terms of PDEs describing i-state development and survival together with a description of the reproduction process [41, 42]. The reproduction process is either modelled as a boundary condition or, if reproduction amounts to jumps in the i-state space, by nonlocal terms in the PDE.

When dealing with measure-valued solutions of PDEs, finding a good definition of a solution is already a non-trivial problem. In some works weak solutions have been considered, but in other cases also more complicated types of solutions have been defined [10, 15, 43]. Proving the existence of such solutions requires tools from functional analysis [11].

A first aim of the present paper is to emphasise, in the spirit of [18] and [53], that one can use the biological interpretation to go directly to an integral equation of variation-of-constants (or Duhamel) type, viz. (1.2), thus avoiding the need to specify the technical sense in which one has to interpret the derivatives in the PDE. Secondly, exploiting the positivity of the kernel $k$, we demonstrate that the existence and the uniqueness of a solution of (1.2) can be proven using well-known methods of the theory of Volterra integral equations [31] or Markov renewal theory [48], even when dealing with measure valued solutions. Thirdly, restrictive, but biologically motivated assumptions on the kernel $k$ allow us to fairly easily deduce the asymptotic behaviour of the solution of (1.2): balanced exponential growth, i.e. $b(t) \sim C e^{ rt } \phi $ is the generic asymptotic large time behaviour of linear renewal equations. (But there are, as we shall see, interesting exceptions.)

These are the main advantages of working with renewal equations. We shall, however, also sketch in Appendix B the PDE formulation and the relation between the PDE and the renewal equation for the models we present in Sect. 4.

Different approaches to the study of the asymptotic behaviour of the solutions of PDEs on measures with applications to structured population dynamics and growth-fragmentation processes can be found in, e.g. [2, 9, 12, 26, 29, 46, 57, 59]. There is a rich body of literature on Volterra integral equations in Banach spaces, see for instance the book [45] by Prüß and the review article [14] by Corduneanu and the references therein. Most of these works focus on situations in which the kernel is an unbounded operator and, as a consequence, already proving existence and uniqueness of solutions can be a formidable task. Moreover, the techniques presented in these works are not suitable for renewal equations with measure-valued solutions. Abstract Volterra equations in the context of population dynamics have been studied, for instance, by Heijmans [36] and Thieme [51].

If $\Omega _{0}$ has finite cardinality we say that there are finitely many states at birth. If there are $n$ states at birth, then $\Omega _{0}=\{\omega _{1}, \ldots ,\omega _{n}\}$ and $b(t,\cdot ) $ can be represented by a vector $b(t)$ in ${\mathbb{R}}^{n}$, with the $k$th component equal to $b(t,\omega _{k})$. This vector satisfies the equation

1.3 $$ b(t) = \int _{0}^{\infty }{\mathbf{K}}(a) b(t-a)da, $$

where $\mathbf{K}$ is an $n \times n$-matrix-valued function. The asymptotic behaviour of solutions of equation (1.3) depends crucially on whether the resolvent kernel of $\mathbf{K}$ belongs to $L^{1}$ or not, [31]. Let $\widehat{K}$ denote the Laplace transform of $K$. The Paley-Wiener Theorem states that the resolvent kernel of an $L^{1}$-kernel $K$ belongs to $L^{1}$ if and only if the *characteristic equation*

1.4 $$ \det \left (I-\widehat{\mathbf{K}}(\lambda )\right ) =0 $$

has no roots $\lambda $ with real part greater than or equal to zero. Because it is often easy to locate the roots of the characteristic equation (1.4) in the complex plane, for instance using positivity arguments or Nyquist’s criterion [31, p. 61], this result provides a powerful tool for analysing the asymptotic behaviour of solutions of (1.3).

If there are infinitely many states at birth, that is, if $\Omega _{0}$ has infinite cardinality, the equation (1.2) can still be written as (1.3) with $b(t) = b(t, \cdot )$, but now $\mathbf{K}(a)$ is a linear operator from the space of measures into itself defined by

$$ {\mathbf{K}}(a)m = \int _{ \Omega _{0}} m(d\xi )k(a,\xi ,\cdot ). $$

Under biologically justified conditions on the kernel $k$, the operator $\mathbf{K}$ is bounded.

In the paper [30] the result for equations in ${\mathbb{R}}^{n}$ presented above was extended to equations in Banach spaces as follows: If $\mathbf{K}$ belongs to $L^{1}(\rho )$, a weighted $L^{1}$-space, the resolvent kernel of $\mathbf{K}$ belongs to $L^{1}(\rho )$ if and only if the operator

1.5 $$ I - \widehat{\mathbf{K}}(\lambda ) $$

is invertible for all $\lambda $ with real part greater than or equal to $\rho $. The main difference between the finite and infinite dimensional cases is that in the infinite dimensional case there is no characteristic equation and it might be exceedingly difficult to check the invertibility of the operator (1.5). An assumption that helps to prove the invertibility of (1.5) is that $\widehat{\mathbf{K}}(\lambda )$ is compact. This helps because then we know that every non-zero element of the spectrum of $\widehat{\mathbf{K}}(\lambda )$ is an eigenvalue. Since compactness is an elusive property when working with measures, see [57, 58], applying the results of [30] is not easy in our case. An additional complication has to do with the fact that Bochner measurability assumptions are required to apply the results of [30].

For these reasons we do not follow [30] and, instead, we present a factorisation assumption on the kernel that allows us to reduce the renewal equation (1.2) to a one dimensional equation. Exploiting this reduction we deduce the behaviour of the solution of (1.2) from Feller’s Renewal Theorem. Thus we obtain strong results for a restricted class of equations.

The paper is organised as follows. In Sect. 2 we present the kernel factorisation that allows for a reduction to a one dimensional equation. We explain the intuitive reasons why we expect this factorisation to simplify the study of the asymptotic behaviour of the solutions of (1.2) and we also motivate the assumption in biological terms. In Sect. 3 we present the main results of this paper: the existence and uniqueness of a solution for equation (1.2), the reduction to a one dimensional equation, and the asymptotic behaviour of the solution of (1.2). Two applications of the results are presented in Sect. 4. The proofs of the main theorems are presented in Sect. 5 and in Sect. 6 we explain the relation between the renewal equations and the PDEs via a semigroup formulation. We conclude the paper with a discussion of possible extensions of the methods presented.

In Appendix A we present the notation we use throughout the paper. Appendix A contains also important definitions and auxiliary results with proofs. In Appendix B we sketch the PDE formulation of the models presented in Sect. 4 and briefly discuss the relation between the PDE and renewal equation approaches.

## Kernel Factorisation

In this section we introduce the main hypothesis of this work: the factorisation property of the kernel.

A *bounded measure component* is a positive function $M:\mathbb{R}_{+} \times \mathscr {B}(\Omega _{0}) \rightarrow \mathbb{R}_{+}$ such that

1. for every $a \in \mathbb{R}_{+}$, $M(a, \cdot ) \in M_{+}(\Omega _{0}) $,
2. for every $\omega \in \mathscr {B}(\Omega _{0}) $ the map $a \mapsto M(a, \omega ) $ is measurable,
3. $\sup _{a \in \mathbb{R}_{+}} M(a, \Omega _{0}) < \infty $.

A *function component* is a positive function $L: \mathbb{R}_{+} \times \Omega _{0} \rightarrow \mathbb{R}_{+} $ such that

1. the map $(a,\xi ) \mapsto L(a, \xi ) $ is measurable,
2. $\sup _{(a, \xi ) \in \mathbb{R}_{+} \times \Omega _{0}} L(a, \xi ) \leq 1 $
3. $L(\cdot , \xi )$ is a non-decreasing function for every $\xi \in \Omega _{0}$.

### Definition 2.1

Factorisable kernel

We say that a kernel $k$ is *factorisable* if there exist two components, a bounded measure component $M$, and a function component $L$, such that, for every $a \in \mathbb{R}_{+}$, $\xi \in \Omega _{0}$ and $\omega \in \mathscr {B}(\Omega _{0})$

2.1 $$\begin{aligned} k(a,\xi , \omega )=\int _{[0, a]} \mu _{L}(d\sigma , \xi ) M(a- \sigma , \omega ). \end{aligned}$$

Observe that properties 2. and 3. of $L$ together imply that $L(\cdot , \xi ) \in NBV(\mathbb{R}_{+})$.

In (2.1) $\mu _{L}(\cdot , \xi ) $ denotes the measure associated with the NBV function $L(\cdot , \xi ) $, as explained in Sect. A.2, Definition A.1. Notice that (2.1) is well defined since, for every $\omega \in \mathscr {B}(\Omega _{0})$, the function $M (\cdot , \omega )$ is Borel measurable and bounded, hence $\mu _{L}(\cdot , \xi )$ integrable for every $\xi \in \Omega _{0}$, see [38] for more details on Lebesgue-Stieltjes integral. In the following $M$ and $L$ always denote components as specified in Definition 2.1.

What causes a kernel to be factorisable? The underlying biological interpretation will be explained in detail by way of examples in Sect. 4, but here we provide a preview. Consider a size structured population in which the size-at-birth of offspring depends, perhaps in a stochastic manner, on the size of the mother. If the smallest mother is larger than the biggest newborn, there is a size that every individual has to pass before it can possibly give birth. We call such a size a renewal state or point. The life cycle can accordingly be split into the phase before passing the renewal point and the phase after passing the renewal point. Mathematically, this is captured by the two components.

The probability of an individual, born with size $x$, passing the renewal state before it has reached age $\sigma $ is $L(\sigma ,x)$. The rate at which an individual, who passed the renewal state $\tau $ time units ago, produces offspring in the set $\omega $ is $M(\tau , \omega )$.

**Fig. 1:**
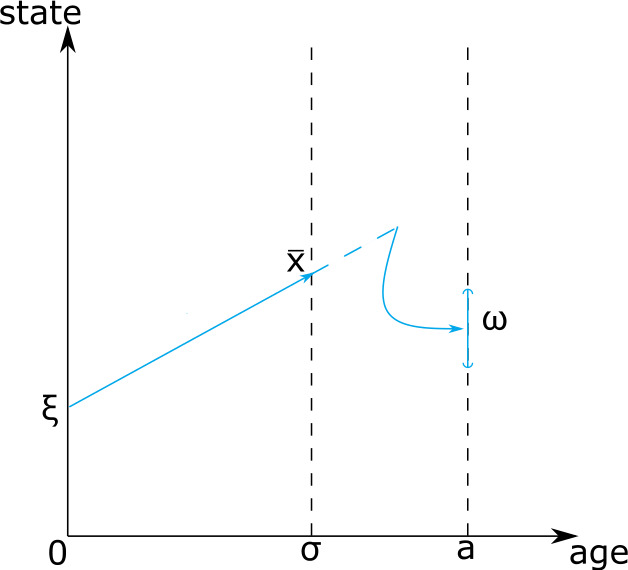
Graphical representation of the factorisation hypothesis

The interpretation of $M(\tau , \omega )$, then, coincides with the one of $k (\tau , \overline{x}, \omega )$ where $\overline{x} $ is the renewal point, but $k(\tau ,\overline{x},\omega )$ may not be defined as $\overline{x}$ may not belong to $\Omega _{0}$. See Fig. 1 for a visual representation of the existence of a renewal point. In the biological examples we are interested in, the function $L(\cdot ,\xi )$ is typically not differentiable, but only of bounded variation. Indeed, if we consider a model with deterministic movement, no mortality and we assume that there exists a renewal point, we obtain that $L(\sigma ,\xi ):= H(\sigma -h(\xi ) ) $, where $h(\xi )$ corresponds to the travel time from $\xi $ to the renewal point and $H$ is the Heaviside function (see Appendix A.1).

For more general models, for example including mortality, $L$ will be of the form

2.2 $$ L(\sigma ,\xi ):= f( \xi ) H(\sigma -h(\xi ) ) $$

where $f$ and $h$ are suitable functions with $0\leq f \leq 1 $. The reason for our characterisation of $L$ as a cumulative quantity is that its derivative is often a measure with a discrete component.

In the remainder of this section we show that for factorisable kernels it is very simple to determine the *basic reproduction number* $R_{0}$ and the *Malthusian parameter* $r$ (definition in Sect. A.4). In analogy with Definition A.22 we next introduce the definition of Laplace components.

One way to think about (2.1) is that the Laplace transform of $k$ (with respect to the variable $a$) is the product of the Laplace transforms of $L$ and $M$ and that, accordingly, the function of $\xi $ and $\omega $ decomposes as a product of a function of $\xi $ and a function of $\omega $. Thanks to the fact that a positive measure is uniquely determined by its Laplace transform, this observation ‘explains’ many of our results and we encourage our readers to reformulate and/or reinterpret various results below in terms of Laplace transforms (we thank Horst Thieme for advocating this point of view in a reaction to an earlier version of the manuscript).

### Definition 2.2

Laplace components

Let $z_{0} \le 0$. We say that $M $ and $L$ are $z_{0}$*-Laplace* components if the Laplace transform exists in the right half plane $\{\lambda \in {\mathbb{C}}: \mathrm{Re}\, \lambda \ge z_{0}\}$. More precisely, the conditions read

$$ \hat{M}(z_{0},\Omega _{0}) :=\int _{0}^{\infty }e^{- z_{0} a} M(a, \Omega _{0}) da < \infty $$

and

$$ \sup _{\xi \in \Omega _{0}} \hat{\mu }_{L}(z_{0},\xi ) = \sup _{\xi \in \Omega _{0}} \int _{0}^{\infty }e^{-z_{0} a} \mu _{L}(da, \xi ) < \infty , $$

respectively.

We refer to Appendix A, Eqs. (A.1) and (A.2) for the definition of the Laplace transform of functions and measures, respectively.

If $M $ and $L$ are $z_{0}$-Laplace components, then we say that the kernel $k $ given by formula (2.1) is a *factorisable* $z_{0}$*-kernel*.

It is reasonable to assume that the kernel $k $ in (1.2) is a Laplace kernel. We recall that, by the interpretation of $k$, an individual born with state $x$ is expected to produce $k(a,x,\omega )$ offspring, with state at birth belonging to $\omega $, per unit of time at age $a$. (The reason why time does not appear in the third argument of $k$ is that, by the definition of age, a newborn has age 0.) The assumption that the kernel $k$ is a Laplace kernel expresses that the expected offspring production decreases exponentially with age.

We now briefly explain the heuristic reason why the basic reproduction number and the Malthusian parameter are expected to determine the asymptotic behaviour of the solution of equation (1.2). From the interpretation of the kernel $k$, it follows that $\int _{0}^{\infty }k(a,\xi , \omega )da$ is the expected number of new individuals with birth state in $\omega $ produced by an individual, itself born with state at birth $\xi $, during its entire life. If we interpret $\phi $ as the distribution of state-at-birth in a certain generation, $\mathbb{K} \phi $ describes the same distribution in the next-generation. The operator $\mathbb{K} $ is called the *next-generation operator* (see the precise definition in Sect. A.4, Definition A.23).

The spectral radius of the next generation operator is the basic reproduction number $R_{0}$. Assume the spectral radius to be a strictly dominant and algebraically simple eigenvalue. The corresponding normalised eigenmeasure $\nu _{0}$ is called the stable distribution since, if we apply the operator $\mathbb{K}$ repeatedly, the population distribution converges to $\nu _{0}$ and the per-generation multiplication number to $R_{0}$. For this reason we can think of $R_{0}$ as the *expected* number of individuals produced by a newborn individual during its entire life.

Here ‘expected’ refers to both the state-at-birth and the reproduction during life. If, once the stable distribution is attained, we pick at random an individual from the pool of newborn individuals in a certain generation, the distribution of its state-at-birth is described by the measure $\nu _{0}$. The kernel $k$ describes the expected offspring production of an individual, conditional on its state-at-birth. The integration with respect to age is expressed in words by ‘during its entire life’. The eigen-relation $\mathbb{K} \nu _{0} = R_{0}\nu _{0} $ then translates into ‘the expected number of offspring equals $R_{0}$’. For more details see [18, 20, 22, 48].

Because of this interpretation, we expect the population birth rate to decline if $R_{0}<1 $, to increase if $R_{0}>1$ and to remain constant if $R_{0}=1 $. In Sect. 3.3, we prove that this is indeed the case. To see how the Malthusian parameter $r$ enters the picture, substitute as an Ansatz $b(t,\omega )= e^{rt}\nu (\omega )$ into (1.2). This leads to

$$ \nu (\omega )=\int _{\Omega _{0}} \hat{k}(r, x, \omega ) \nu (dx) $$

(cf. (A.22) in Definition A.25 in the appendix) as a condition on the combination of $r$ and $\nu $. More generally we may replace $r$ by a complex parameter $\lambda $, but positivity arguments guarantee that $\mathrm{Re}\,\lambda < r$ if $\mathrm{Im}\, \lambda \neq 0$ (with equality in exceptional situations). This is the reason why the Malthusian parameter is expected to be the population growth rate.

### Theorem 2.3

Spectral radius of the next generation operator

*Assume* $k$ *is a factorisable* $z_{0}$-*kernel with components* $M $ *and* $L$. *The next generation operator* (*cf*. *Definition* A.23) $\mathbb{K} : M_{+,b} (\Omega _{0}) \rightarrow M_{+,b} (\Omega _{0}) $, *corresponding to the kernel* $k$, *has one dimensional range*. *The only non*-*zero eigenvalue of* $\mathbb{K}$ *is*

2.3 $$ R_{0}= \int _{0}^{\infty }\int _{\Omega _{0}} M(a, d\xi ) \lim _{ \sigma \rightarrow \infty } L(\sigma , \xi ) da $$

*and it corresponds to the eigenmeasure* $\hat{M}(0,\omega ),\,\, \omega \in \mathscr {B}(\Omega _{0})$.

### Proof

It is enough to realise that

$$\begin{aligned} (\mathbb{K} \varphi )(\omega )& = \int _{\Omega _{0}}\varphi (d\xi ) \int _{0}^{\infty }\int _{0}^{a} \mu _{L}(d\sigma ,\xi ) M(a-\sigma , \omega ) da \\ &=\int _{0}^{\infty }M(\alpha , \omega ) d\alpha \int _{\Omega _{0}} \varphi (d\xi ) \int _{0}^{\infty }\mu _{L}(d\sigma ,\xi ) . \end{aligned}$$

 □

We show now that, under the factorisation assumption, the Malthusian parameter $r$ (see Sect. A.4 for a definition) is the unique solution of a characteristic equation defined in terms of the Laplace transform of a scalar measure. At the same time we will show that the factorisation assumption allows us to easily deduce that $\mathrm{sign}(R_{0}-1) = \mathrm{sign}(r)$.

Let us define $K: \mathbb{R}_{+} \rightarrow \mathbb{R}_{+}$ by

2.4 $$ K(\tau ):= \int _{0}^{\tau }\int _{\Omega _{0}} M(\eta ,d\xi ) L(\tau - \eta , \xi ) d\eta . $$

Notice that $K$ is well defined since the function

$$ \eta \mapsto \int _{\Omega _{0}} M(\eta ,d\xi ) L(\tau -\eta , \xi ) $$

is measurable by Lemma A.10.

### Proposition 2.4

*Let* $M$ *and* $L $ *be Laplace components*. *The function* $K$, *defined by* (2.4) *is a non*-*decreasing and right*-*continuous function on* $\mathbb{R}_{+}^{*}$.

### Proof

$K$ is non-decreasing because $L(\cdot ,\xi )$ is non-decreasing for every $\xi \in \Omega _{0}$. By the monotone convergence theorem $K$ is right-continuous because $L(\cdot , \xi )$ is right-continuous for every $\xi \in \Omega _{0}$. □

Observe that by combining (2.3) with the definition (2.4) of K it follows that

$$ R_{0}= \int _{0}^{\infty }\mu _{K} (d\tau ) = \lim _{ t \rightarrow \infty } K(t). $$

### Lemma 2.5

*Let* $M,L , K $ *be as in Proposition* 2.4. *If* $R_{0} \geq 1$, *then the characteristic equation*

2.5 $$ \hat{\mu }_{K}(z) :=\int _{0}^{\infty }e^{-z \tau } \mu _{K}(d\tau ) =1 $$

*has a unique real solution* $r \geq 0$. *If* $R_{0} < 1$ *and there exists a* $z<0$ *such that*

2.6 $$ \hat{\mu }_{K}(z) >1, $$

*then* (2.5) *has a unique real solution* $r<0$.

### Proof

Assume $R_{0} \geq 1$ and notice that by the dominated convergence theorem the function $z \mapsto \hat{\mu }_{K}(z) $ is a strictly monotone continuous function and takes value $R_{0} \geq 1 $ when $\lambda =0$. Moreover, it tends to 0 as $\lambda $ goes to infinity. Therefore, by the intermediate value theorem we deduce that there exists a unique non-negative solution of the characteristic equation.

Assume now $R_{0} < 1$ and that for some $z<0$ inequality (2.6) holds. Then the statement follows by applying the previous argument. □

When we in the following deal with the case $r < 0$ we shall tacitly assume that $K$ is such that existence of $r$ is indeed guaranteed, i.e., that there exists a $z<0$ such that (2.6) holds.

Assume $k $ is a factorisable $z_{0}$-kernel, with components $M$ and $L$. By the factorisation assumption the eigenproblem (see (A.22) in Appendix A) reads

2.7 $$\begin{aligned} \nu (\omega )=\int _{\Omega _{0}} \hat{k}(r, \xi , \omega ) \nu (d \xi ) = \int _{\Omega _{0}} \hat{ \mu _{L}} (r, \xi ) \nu (d\xi ) \hat{M}(r, \omega ). \end{aligned}$$

This implies that the eigen-measure is of the form $\alpha \hat{M}(r, \omega ) $ and the solution of the characteristic equation (2.5) is the Malthusian parameter (Definition A.25 in Appendix A).

## Main Results

### Existence and Uniqueness of the Solution of the Renewal Equation

In this section we consider the existence and uniqueness of solutions of the renewal equation (1.2). To do so we assume that, as an initial condition, $b(t, \cdot )$ is given on $(-\infty ,0]$. We next split the integral over $(0,\infty )$ in (1.2) into two integrals over $(0,t]$ and $(t,\infty )$, respectively. Then (1.2) takes the following form:

3.1 $$\begin{aligned} b(t, \omega ) = \int _{0}^{t} \int _{\Omega _{0}} k(t-s, \xi ,\omega ) b(s, d\xi ) ds + f_{0}(t, \omega ) \quad t >0, \quad \omega \in \mathscr {B}(\Omega _{0}) \end{aligned}$$

We start by specifying what kind of initial condition we consider and how the initial condition is reflected in the forcing function $f_{0}$. We allow the initial condition to be measure in both variables simply because it does not harm. We introduce a weight $\exp (-z_{0}\sigma )$ to include initial conditions with constant birth rates.

#### Definition 3.1

Initial condition

Let $z_{0} \leq 0$. We say that the measure $\Phi $ on the product $\sigma $-algebra $\mathscr {B}(\mathbb{R}^{-}) \times \mathscr {B}(\Omega _{0})$ is a $z_{0}$-initial condition if

$$ \int _{-\infty }^{0} \int _{\Omega _{0}} e^{- z_{0} \sigma } \Phi (d \sigma , dx) < \infty . $$

#### Definition 3.2

Forcing function

Let, for some $z_{0} \leq 0$, $k$ be a $z_{0}$-kernel and $\Phi $ a $z_{0}$-initial condition. The forcing function corresponding to $k$ and $\Phi $ is the function $f_{0}: \mathbb{R}_{+} \times \mathscr {B}(\Omega _{0}) \rightarrow \mathbb{R}_{+}$ defined by

3.2 $$ f_{0}(t, \omega ):= \int _{-\infty }^{0} \int _{\Omega _{0}} k(t- \sigma , x, \omega ) \Phi ( d\sigma , dx ) $$

The interpretation of the forcing function is that $f_{0}(t, \omega )$ is the rate at which offspring of individuals that were themselves born before time 0, are born with state-at-birth in $\omega \subset \Omega _{0}$ at time $t$.

#### Remark 3.3

Notice that, using the Fubini-Tonelli theorem, we can deduce that

$$\begin{aligned} \int _{0}^{\infty }e^{-z_{0} t} f_{0}(t, \Omega _{0}) d t &= \int _{0}^{\infty }e^{ -z_{0} t} \int _{-\infty }^{0} \int _{\Omega _{0}} k(t- a, x, \Omega _{0}) \Phi (da, dx ) dt \\ & = \int _{0}^{\infty }\int _{-\infty }^{0} \int _{\Omega _{0}} k(t- a, x, \Omega _{0}) e^{ z_{0}(a-t)} e^{ - z_{0} a} \Phi (da, dx) dt \\ & \leq \int _{-\infty }^{0} \int _{\Omega _{0}} \int _{0}^{\infty }k(t-a, x, \Omega _{0}) e^{z_{0}(a-t)} d t e^{ - z_{0} a} \Phi (da, dx ) \\ & \leq \sup _{ x \in \Omega _{0}} \int _{0}^{\infty }k(\tau , x, \Omega _{0}) e^{ - z_{0} \tau } d\tau \int _{-\infty }^{0} \int _{ \Omega _{0}} e^{ - z_{0} a} \Phi ( da, dx ) < \infty . \end{aligned}$$

The interpretation of these inequalities is that the contribution to the population birth rate of the individuals born before time 0 tends to zero exponentially as time tends to infinity. This gives an additional motivation to the weight $\exp (-z_{0}\sigma )$ in Definition 3.1 and in the definition of the $z_{0}$-kernels.

#### Theorem 3.4

Existence and uniqueness

*Let* $k$ *be a factorisable kernel and let* $f_{0}$ *be a forcing function*. *Then the renewal equation* (3.1) *has a unique solution*.

### One Dimensional Representation

In this section we introduce, whenever the kernel is factorisable, a renewal equation such that its scalar solution $B(t)$ contains all relevant information needed to recover the solution $b(t,\cdot )$ of (3.1). We call this *one dimensional reduction* or *representation* of (3.1). The renewal equation (3.5) below is called the *reduced equation*. This reduction will be crucial for the proof of the theorems on the asymptotic behaviour of the solutions of (3.1). The solution $B(t)$ of the reduced equation has, in Sect. 4, the interpretation that it is the (cumulative) number of individuals that were born after time zero and have passed the renewal point before time $t$.

We start by defining the scalar function $B$ with the above mentioned interpretation. Then we check that it is locally of bounded variation and satisfies a renewal equation.

#### Definition 3.5

Let $k $ be a factorisable $z_{0}$-kernel with function component $L$ and let $f_{0}$ be a forcing function. The *cumulative number of renewal events* is, as a function of time, given by $B : \mathbb{R}_{+} \rightarrow \mathbb{R}_{+}$ defined by

3.3 $$\begin{aligned} B(t)& := \int _{0}^{t} \int _{\Omega _{0}} b(t-\sigma , d\xi ) L( \sigma ,\xi ) d \sigma , \quad t >0, \\ B(0)&:=0, \end{aligned}$$

where $b$ is the unique solution of (3.1).

#### Proposition 3.6

*The cumulative number of renewal events corresponding to the factorisable* $z_{0}$-*kernel* $k$, *with function component* $L$ *and forcing function* $f_{0}$, *belongs to* $NBV_{\mathrm{loc}}(\mathbb{R}_{+})$.

#### Proposition 3.7

*Let* $K$ *be the kernel defined by* (2.4) *and let*

3.4 $$ z(t):=\int _{0}^{t} \int _{\Omega _{0}} f_{0}(t-\sigma ,d\xi ) L( \sigma ,\xi ) d\sigma . $$

*The cumulative number* $B$ *of renewal events is the unique solution of the one dimensional renewal equation*

3.5 $$\begin{aligned} B(t)&=\int _{0}^{t} \mu _{B}(d \tau ) K(t-\tau ) +z(t) \quad t>0. \end{aligned}$$

The integral in (3.5) is well defined because both $K$ and $B$ are locally of bounded variation (Propositions 2.4 and 3.6). The function $z $ is also well defined since the function

$$ \sigma \mapsto \int _{\Omega _{0}} f_{0}(t-\sigma ,d\xi ) L(\sigma , \xi ) $$

is measurable thanks to Lemma A.10. If we assume that there exists a renewal point, we can interpret $z(t)$ as the (cumulative) number of offspring of individuals born before time 0, that passed the renewal point before time $t$.

For later reference we now state some properties of the forcing function $z$ and the kernel $K$ in the reduced renewal equation (3.5).

#### Lemma 3.8

*We have*

1. $z_{\infty }:=\lim _{t \rightarrow \infty } z(t)< \infty $;
2. $\int _{0}^{\infty }s e^{- w s} \mu _{K}( ds) < \infty $ *for every* $w > z_{0}$;
3. *If* $z_{0} < w \leq 0$, *then* $\int _{0}^{\infty }e^{-w s} ( z_{\infty }-z(s) ) ds < \infty $;
4. *If* $w>0$, *then* $\hat{z}(w)=\int _{0}^{\infty }z(s ) e^{- w s} ds < \infty $.

We are now ready to state our result on how the population birth rate is represented by the cumulative number of renewal events.

#### Theorem 3.9

One dimensional representation

*Let* $k$ *be a factorisable* $z_{0}$-*kernel*, *with components* $L$ *and* $M$, *and let* $f_{0}$ *be a forcing function*. *Let* $B $ *be the cumulative number of renewal events*, *that is*, *the unique solution of* (3.5). *Then*, *the identity*

3.6 $$ b(t,\omega )=\int _{0}^{t} \mu _{B}(d\sigma ) M(t-\sigma , \omega )+f_{0}(t, \omega ), \quad t >0, \quad \omega \in \mathscr {B}(\Omega _{0}) $$

*holds in the following weak sense*: *for every* $\omega \in \mathscr {B}(\Omega _{0})$ *and for every* $t >0$

3.7 $$ \int _{0}^{t} b(\sigma ,\omega ) d\sigma =\int _{0}^{t} B(\sigma ) M(t- \sigma , \omega ) d\sigma + \int _{0}^{t} f_{0}(\sigma , \omega ) d \sigma . $$

#### Remark 3.10

We underline that (3.6) can be seen as an equality between measures. Indeed we can define $\tilde{b} \in M_{+} (\mathbb{R}_{+} \times \Omega _{0}) $ by

$$ \tilde{b}([0,t], \omega ):= \int _{0}^{t} b(\sigma ,\omega ) d\sigma \quad t >0, \quad \omega \in \mathscr {B}(\Omega _{0}). $$

Equality (3.7) can, consequently, be reinterpreted as

3.8 $$\begin{aligned} \tilde{b}( [0,t] ,\omega ) &=\int _{0}^{t} B(\sigma ) M(t-\sigma , \omega ) d\sigma + \tilde{f_{0}}([0,t], \omega ) \end{aligned}$$

where $\tilde{f_{0}} $ is a measure on the product Borel $\sigma $-algebra $\mathscr {B} (\mathbb{R}_{+}) \times \mathscr {B}(\Omega _{0}) $ defined by

$$ \tilde{f_{0}}([0,t], \omega ):= \int _{0}^{t} f_{0}(\sigma , \omega ) d \sigma . $$

To understand the intuitive idea behind the theorem it is convenient to assume the existence of a renewal point. Note that

$$ \mu _{B}(d\tau ) M(t-\tau , \omega ) $$

can be interpreted as the expected number, per unit of time, of newborns in $\omega $ produced at time $t$ by individuals that passed the renewal state at time $t-\tau $, so $\tau $ time units ago; and we condition on $\tau < t $, i.e., on passage after time zero. Integrating over all possible passage times, we obtain the population birth rate.

### Asymptotic Behaviour

In this section we state results on the asymptotic behaviour of the cumulative number $B(t)$ of renewal events, the cumulative population birth rate $\int _{0}^{t} b(\tau ,\cdot )d\tau $ and the population birth rate $b(t,\cdot )$. The results will be proved in Sect. 5 using Feller’s Renewal Theorem concerning the asymptotic behaviour of solutions of renewal equations. This is a natural approach because these three functions all satisfy renewal equations. We therefore start by recalling a definition, taken from Feller’s book [27].

#### Definition 3.11

Arithmetic function

We say that a mapping of $\mathbb{R}_{+}$ into $\mathbb{R}_{+} $ is arithmetic if it is a step function with jump discontinuities at the points $\alpha + n h$ with $n$ varying in ℕ and with $\alpha $ and $h $ two fixed non-negative numbers.

We now state additional assumptions and specify the notation. These pertain to the rest of the section and will not be repeated.

#### Notation 3.12

Let $M$ and $L$ be $z_{0}$-Laplace components with $z_{0} <0$. We assume that $\Phi $ is an $z_{0}$-initial condition. Then,

- $k$ denotes the factorisable $z_{0}$-kernel given by formula (2.1);
- $f_{0}$ denotes the forcing function, corresponding to $\Phi $ and $k$, given by formula (3.2);
- $b$ denotes the solution of equation (3.1) with kernel $k$ and forcing function $f_{0}$;
- $B$ denotes the cumulative number of renewal events corresponding to $L$ and $b$ as introduced in Definition 3.5 and subsequently characterised in Proposition 3.7;
- $K$ denotes the function defined by (2.4);
- $r$ denotes the Malthusian parameter, i.e., the unique real solution of the characteristic equation (2.5);
- A hat $\hat{\phantom{.}}$ denotes the Laplace transform of a function or a measure;
- $z$ denotes the function $z: \mathbb{R}_{+} \rightarrow \mathbb{R}_{+}$ defined by (3.4) and $z_{\infty }:=\lim _{t \to \infty }z(t)$.
- The constants $C_{r},\, K_{r},\, D$ and $B_{\infty }$ are defined as follows:

$$\begin{aligned} C_{r} := &\frac{\hat{z}(r)}{\int _{0}^{\infty }s e^{- r s } \mu _{K}(d s ) }, \\ K_{r} := &\frac{z_{\infty }/r - \int _{0}^{\infty }e^{-r s} ( z_{\infty }-z(s) ) ds }{\int _{0}^{\infty }s e^{-rs} \mu _{K}(ds) }, \\ D :=&\frac{z_{\infty }}{\int _{0}^{\infty }s \mu _{K}(ds) } \\ B_{\infty } :=& \lim _{t \rightarrow \infty }B(t). \end{aligned}$$

Because $B$ is non-decreasing the limit $B_{\infty }$ exists, but it may be equal to infinity.

In the following theorems the symbol ∼ is used to denote setwise asymptotic equivalence as explained in Sect. A.1, Eq. (A.4).

#### Theorem 3.13

Asymptotic behaviour of the cumulative number of renewal events

*Let* $K $ *be non*-*arithmetic*. *The following asymptotics hold for the cumulative number* $B$ *of renewal events*:

1. *If* $r>0$, *then* $B(t) \sim C_{r} e^{ t r }$ *as* $t \rightarrow \infty $;
2. *If* $z_{0}< r<0$, *then* $B_{\infty }< \infty $ *and* $B(t) \sim B_{\infty }+ e^{tr } K_{r}$, *as* $t \rightarrow \infty $;
3. *If* $r=0$, *then* $B(t) \sim t D$ *as* $t \rightarrow \infty $.

#### Theorem 3.14

Setwise asymptotic behaviour of the cumulative population birth rate

*Assume that* $K$ *is non*-*arithmetic*.

1. *If* $r > 0$, *then*,
  $$ \int _{0}^{t} b(\tau ,\cdot ) d\tau \sim C_{r} e^{r t} \hat{M}(r, \cdot ) \quad \textit{ setwise as } t \rightarrow \infty , $$
2. *If* $z_{0} < r<0$, *then*, $B_{\infty }< \infty $ *and*
  $$ \int _{0}^{t} b(\tau ,\cdot ) d\tau \sim K_{r} e^{r t} \hat{M}(r, \cdot ) +B_{\infty }\hat{M}(0,\cdot ) +\int _{0}^{\infty }f_{0}(\tau , \cdot ) d\tau \quad \textit{ setwise as } t \rightarrow \infty , $$
3. *If* $r=0$, *then*
  $$ \int _{0}^{t} b(\tau ,\cdot ) d\tau \sim t D \hat{M}(0,\cdot ) \quad \textit{ setwise as } t \rightarrow \infty . $$

In the following corollary we state the results on asymptotic behaviour in terms of the total variation norm and the flat norm, respectively (see Definitions A.3, A.4).

#### Corollary 3.15

Asymptotic behaviour, in norm, of the cumulative population birth rate

*Let* $\| \cdot \|=\| \cdot \|_{TV} $ *or* $\| \cdot \| = \|\cdot \|_{\flat }$. *Assume that* $K$ *is non*-*arithmetic*.

1. *If* $r > 0$, *then*
  $$ \lim _{t \rightarrow \infty } \left \| e^{- r t} \int _{0}^{t} b( \sigma , \cdot ) d\sigma - C_{r} \hat{M}(r, \cdot ) \right \| =0. $$
2. *If* $z_{0} < r<0$, *then*
  $$ \lim _{t \rightarrow \infty } \left \| \int _{0}^{t} b(\sigma , \cdot ) d\sigma - e^{ r t} K_{r} \hat{M}(r, \cdot ) - B_{\infty }\hat{M}(0, \cdot ) - \int _{0}^{\infty }f_{0}(\sigma , \cdot ) d \sigma \right \| = 0. $$
3. *If* $r=0$, *then*
  $$ \lim _{t \rightarrow \infty } \left \| \frac{1}{t} \int _{0}^{t} b( \sigma , \cdot ) d\sigma - D \hat{M}(0,\cdot ) \right \| =0. $$

We next describe the asymptotic behaviour of the population birth rate $b$ under the following additional assumption.

#### Assumption 3.16

The functions $z$ and $K$ are absolutely continuous functions and, therefore, there exist two integrable functions $z' : \mathbb{R}_{+}\rightarrow \mathbb{R}_{+}$ and $K': \mathbb{R}_{+}\rightarrow \mathbb{R}_{+}$ such that

$$ z(t)=\int _{0}^{t} z'(s) ds \quad \text{ and }\quad K(t)=\int _{0}^{t} K'(s)ds. $$

#### Theorem 3.17

Setwise asymptotic behaviour of the population birth rate

*Let* $K$ *and* $z$ *satisfy Assumption* 3.16. *We have*

1. *If* $r>0$, *then*
  $$ b(t, \cdot ) \sim r C_{r} e^{ rt} \hat{M}(r, \cdot ) \textit{ setwise as } t \rightarrow \infty . $$
2. *If* $z_{0} < r<0$, *then*
  $$ b(t, \cdot ) \sim r K_{r} e^{ rt} \hat{M}(r, \cdot ) \textit{ setwise as } t \rightarrow \infty . $$
3. *If* $r=0$, *then*
  $$ b(t, \cdot ) \sim D \hat{M}(0, \cdot ) \textit{ setwise as } t \rightarrow \infty . $$

#### Corollary 3.18

Asymptotic behaviour, in norm, of the population birth rate

*Let* $\| \cdot \|=\| \cdot \|_{TV} $ *or* $\| \cdot \| = \|\cdot \|_{\flat }$. *Let* $K$ *and* $z$ *satisfy Assumption* 3.16.

1. *If* $r > 0$, *then*
  $$ \lim _{t \rightarrow \infty } \left \| e^{- r t} b(t, \cdot ) - r C_{r} \hat{M}(r, \cdot ) \right \| =0; $$
2. *If* $z_{0} < r<0$, *then*
  $$ \lim _{t \rightarrow \infty } \left \| b(t, \cdot ) -r e^{ r t} K_{r} \hat{M}(r, \cdot ) \right \| = 0; $$
3. *If* $r=0$, *then*
  $$ \lim _{t \rightarrow \infty }\left \| b(t, \cdot ) - D \hat{M}(0, \cdot ) \right \| =0. $$

## Application to Two Structured Population Models

In this section we apply the results of Sect. 3.3 to two structured population models that have motivated the current research. The first one concerns a size structured cell population and the second one a population structured by the immune level of individuals. Although the two models describe completely different biological phenomena, they both lead to renewal equations with factorisable $z_{0}$-kernels and they can thus be treated in a unified way.

In Appendix B we briefly discuss for both examples an alternative modelling approach based on PDEs for the density $n(t,x)$ of individuals with size or immune level $x$ at time $t$.

### Cell Growth and Fission

Consider a population of cells reproducing by fission and structured by size. Size could for instance mean mass, volume, length, etc., the important thing is that the quantity is conserved at cell fission, that is, the sum of the sizes of the newly born daughter cells equals the size of the dividing mother cell. We assume that there is a maximum size (normalised to $x=1$) beyond which a cell cannot grow. The individual state space will therefore be the open interval $\Omega =(0,1)$. We assume that cells grow deterministically with the size dependent individual growth rate $g$ and denote the size dependent fission and death rates by $\gamma $ and $\mu $, respectively. We allow for unequal division and let $\eta (x,\omega )$ denote the expected number of individuals born with size in the set $\omega \in \mathscr {B} (\Omega _{0})$ by fission of an individual of size $x$. Recall that $\Omega _{0}$ denotes the set of possible states at birth.

In the next Assumption 4.1 we collect measurability and smoothness assumptions on the model ingredients as well as a consistency relation (clearly, $\gamma , \, \eta $ and $\Omega _{0}$ cannot be independent) and formalise the requirement that size is conserved at fission.

#### Assumption 4.1

**Fig. 2:**
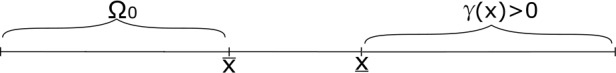
Depiction of the i-state space $\Omega $ as the union of the set $\Omega _{0}$ of states-at-birth, the set $[\overline{x},\underline{x}]$ of points that can serve as renewal states, and a set of i-states with positive reproduction ability

1. $\gamma : (0,1] \rightarrow \mathbb{R}_{+}$ is a measurable function, which is continuous on its support;
2. $\mu : \Omega \rightarrow \mathbb{R}_{+}$ is a measurable bounded function.
3. $g: (0,1] \rightarrow \mathbb{R}^{*}_{+}$ is a strictly positive continuous bounded function, such that
  4.1 $$ \int _{x}^{1} \frac{\gamma (z)}{g(z)} dz =\infty \quad \text{ for all } x \in \Omega . $$
4. $\eta (x, \cdot )$ is a Borel measure for every $x \in \Omega $.
5. $$ \Omega _{0}:=\bigcup _{\{x \in \Omega : \gamma (x)>0\}} \mathrm{supp} (\eta (x, \cdot )). $$
6. $\eta (x, \omega )=\eta (x, \tilde{\omega }_{x})$ for every $x \in \Omega $ and every $\omega \in \mathscr {B}(\Omega _{0})$, where $\tilde{\omega }_{x} $ is the complementary set of $\omega $ with respect to $x$ defined as $\tilde{\omega }_{x}:=\{ y \in \Omega _{0} : x-y \in \omega \}$. Moreover, $\eta (x,\Omega _{0})=2$ for any $x \in \Omega $;
7. $\max {\Omega _{0}}=:\overline{x} < \underline{x}:=\inf \{x \in \Omega : \gamma (x)>0 \} $. See Fig. 2 for a graphical representation of $\Omega_{0}.$

From the basic model ingredients we now derive compound ingredients that will constitute the kernel of the renewal equation of the cell fission model.

By the definition of individual growth rate, the size $X(a,\xi )$ of an individual that $a$ time units ago had size $\xi $ and did not divide, is the solution of the initial value problem

4.2 $$ \frac{d x}{da} = g(x) \quad x(0)=\xi . $$

The time $\tau (x,y)$ it takes to grow from size $x$ to size $y$ is given by

4.3 $$ \tau (x,y):=\int _{x}^{y} \frac{1}{g(z)} dz \quad x, y \in \Omega . $$

It is convenient to introduce the notation $T(x):=\tau (\overline{x}, x)$.

The probability that an individual that $a $ time units ago had size $\xi $ survives to the current time (that is, neither dies nor divides during the time interval $[t-a,t]$) is

$$ \mathscr {F}(a,\xi ):=e^{- \int _{0}^{a} \left ( \gamma (X(s,\xi ))+ \mu (X(s,\xi )) ds \right )}. $$

The probability that an individual that was alive with size $x$ survives at least until reaching size $y$ is

$$ \hat{\mathscr {F}} (x,y):=\mathscr {F}(\tau (x,y),x) =\exp \left (-\int _{x}^{y} \frac{\gamma (z) + \mu (z)}{g(z) } dz \right ) \quad x, y \in \Omega . $$

Notice that it follows from (4.1) that the probability that a cell reaches size 1 is zero.

Consider now a cell born with size $\xi $. With probability $\mathscr {F}(a,\xi )$ it will still be alive at age $a$ and if it is, it will have size $X(a,\xi )$ and divide with probability per unit of time $\gamma (X(a,\xi ))$ giving rise to $\eta (X(a,\xi ),\omega )$ daughter cells in the set $\omega $. The kernel of the cell fission model is therefore given by

4.4 $$ k(a,\xi ,\omega )= \mathscr {F}(a,\xi ) \gamma (X(a,\xi )) \eta (X(a, \xi ),\omega ) $$

for $a \in \mathbb{R}_{+}$, $\xi \in \Omega _{0}$ and $\omega \in \mathscr {B}(\Omega _{0})$. We will now show that this kernel is factorisable.

The key assumption, that allows the reduction, is that the biggest daughter is always smaller than the smallest mother, i.e. Assumption 4.1 7. Any point between $\overline{x} $ and $\underline{x}$ can serve as a renewal point. We choose $\overline{x} $.

We shall prove that the kernel (4.4) is factorisable under two alternative assumptions on $g$ and $\gamma $, which both guarantee that (4.1) holds.

#### Assumption 4.2

Either

1. $g$ is differentiable in $x= 1$ with $g(1)=0$ and $g'(1)\neq 0$ and $\gamma $ is continuous on $(0, 1]$ with $\gamma (1) >0$,

or

2. $g(1)>0 $ and $\gamma $ is such that (4.1) holds.

In case 1, we have $\tau (x, 1) =+\infty $ for every $x \in \Omega $ and we will therefore refer to this case as the case of unbounded lifetime. On the other hand, in case 2, we have $\tau (x,1)< \infty $ for every $x \in \Omega $ and we refer to this case as the case of bounded lifetime.

#### Lemma 4.3

*Under Assumptions* 4.1*and* 4.2*the kernel* $k$ *given by* (4.4) *is a factorisable* $z_{0}$-*kernel*, *with* $z_{0} >- \gamma (1) $ *in the case of unbounded lifetime and any* $z_{0}<0$ *in the case of bounded lifetime*. *In fact*,

$$ k(a,\xi ,\omega )= \int _{0}^{a} \mu _{L}(d\sigma ,\xi ) M(a-\sigma , \omega ) $$

*with*

4.5 $$ M(t,\omega )= \mathscr {F}(t,\overline{x})\gamma (X(t,\overline{x})) \eta (X(t,\overline{x}), \omega ) $$

*in the case of unbounded lifetime*, *and*

$$ M(t, \omega )= \textstyle\begin{cases} \mathscr {F}(t,\overline{x})\gamma (X(t,\overline{x}))\eta (X(t, \overline{x}), \omega ) &\textit{ if } t \leq T(1) \\ 0 &\textit{ if } t > T(1) \end{cases} $$

*if the lifetime is bounded*. *In both cases we have*

4.6 $$ L(t,\xi )= \hat{\mathscr {F}}(\xi ,\overline{x}) H(t-\tau (\xi , \overline{x})). $$

#### Proposition 4.4

*Let the assumptions of Lemma* 4.3*hold*. *Let* $\| \cdot \|=\| \cdot \|_{TV}$ *or* $\| \cdot \|_{\flat }$. *If either*

1. $\eta (x, \omega )=2 \delta _{\frac{1}{2}x}(\omega )$, $g(2x) <2g(x)$ *for every* $x \in \Omega $ *and every* $\omega \in \mathscr {B}(\Omega _{0})$

*or*

2. *for every* $x \in \Omega $ *the measure* $\eta (x,\cdot )$ *is absolutely continuous with respect to the Lebesgue measure and has bounded density* $d$,

*then the solution* $b$ *of* (3.1) *corresponding to the kernel* (4.4) *satisfies*

1. *if* $r>0$, *then* $\|e^{-rt} b(t, \cdot ) - r C_{r} \hat{M}(r,\cdot ) \| \rightarrow 0 $ *as* $t\rightarrow \infty $;
2. *if* $z_{0}< r<0$, *then* $\|e^{-rt} b(t, \cdot ) - r K_{r} \hat{M}(r,\cdot ) \| \rightarrow 0 $ *as* $t\rightarrow \infty $;
3. *if* $r=0$, *then* $\| b(t, \cdot ) - D \hat{M}(0,\cdot ) \|\rightarrow 0 $ *as* $t\rightarrow \infty $.

Thus, if $r \neq 0$ we have balanced exponential growth or decline, with stable distribution $\hat{M}(r,\cdot )$ for the population birth rate. On the other hand, if $r=0$ the population birth rate converges to a steady state. This result is in agreement with the one obtained in [23] and [35], see also [41], but here we allow the population birth rate to be a measure in size.

Because of the sign equivalence $\mathrm{sign}(R_{0}-1) = \mathrm{sign}(r)$ the most convenient way to decide the sign of $r$ is to compute the basic reproduction number

4.7 $$\begin{aligned} R_{0} & =\int _{0}^{\infty }\int _{\Omega _{0}} \hat{\mathscr {F}}(\xi , \overline{x}) \mathscr {F}(a, \overline{x}) \gamma (X(a, \xi ) ) \eta (X(a, \overline{x}) , d\xi ) da. \end{aligned}$$

We next give an example of $K $ being arithmetic.

#### Example 4.5

We show that $K$ is arithmetic if $\eta (x, \omega )=2 \delta _{\frac{1}{2}x}(\omega ) $, $g(x)=x$, $\Omega =[1/4, 1]$, $\overline{x} =1/2$ and $\Omega _{0}=[1/4, 1/2)$. With this choice of $g$ we notice that the time necessary to develop from size $x\in \Omega $ to size $2x\in \Omega $ is constant. Indeed,

$$ \tau (x,2x)= \int _{x}^{2x} \frac{1}{z} dz = \ln 2, \quad x \in \Omega . $$

The function $K: \mathbb{R}_{+} \rightarrow \mathbb{R}_{+}$ reduces to

$$\begin{aligned} K(s)& = \int _{0}^{s} \mathscr {F}(v, \overline{x}) \gamma (X(v, \overline{x}) ) H\left (s-v- \tau \left (\frac{1}{2} X(v, \overline{x}), \overline{x} \right )\right ) dv \\ &= \int _{0}^{s} \mathscr {F}(v, \overline{x}) \gamma (X(v,\overline{x}) ) H\left (s-\tau \left (\frac{1}{2} X(v, \overline{x}), X(v,\overline{x}) \right ) \right )dv \\ &= \int _{0}^{s} \mathscr {F}(v, \overline{x}) \gamma (X(v,\overline{x}) )dv H\left (s-\ln 2 \right ) \\ &= \textstyle\begin{cases} 0 &\text{ if } s < \ln 2, \\ \int _{0}^{\ln 2}\mathscr {F}(v , \overline{x}) \gamma (X(v,\overline{x}) ) dv &\text{ if } s \geq \ln 2 \end{cases}\displaystyle \end{aligned}$$

and so $K$ is arithmetic. This means that we cannot understand the asymptotic behaviour of $b(\cdot , \omega )$ with the method and results of this paper. We refer to [7, 28, 41] and the references in there for more details and results on the arithmetic case.

### Waning and Boosting

Consider a population of individuals characterised by the immune level, that changes continuously due to waning while experiencing (instantaneous) boosting when infection occurs. We assume that the possible immune states belong to the finite interval $\Omega :=(0, x_{m}]$. The immunity decreases deterministically between boosting events with a rate $g$ depending on the immunity level. The rate ℒ at which boosting takes place is assumed to be state dependent. In fact, we shall take a step function for ℒ. The immune state right after infection depends via a function $f$ on the state just before infection.

Here are the assumptions we make on the rates:

#### Assumption 4.6

1. There exist an $\overline{x} \in (0,x_{m})$ and a $\Lambda >0$ such that
  $$ \mathscr {L} (x)= \textstyle\begin{cases} \Lambda & \text{if } \, x \in (0,\overline{x}) \\ 0 & \text{if } \, x \in [\overline{x},x_{m}] \end{cases} $$
2. Let $x_{c} = (x_{m}- \overline{x})/2$. Then $f: (0,\overline{x}) \rightarrow (0,\overline{x})$ is given by
  $$ f(x)= \textstyle\begin{cases} &\overline{x}+2x_{c}-2 x \quad \text{ for } 0 < x < x_{c}, \\ &\overline{x} \quad \text{ for } x_{c} < x < \overline{x}. \end{cases} $$
3. $g$ is a strictly negative continuous function on $\Omega $ with $g(0)=0$

**Fig. 3:**
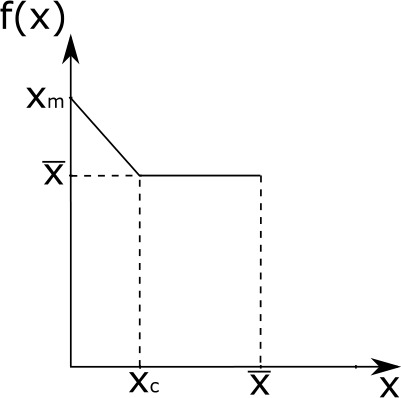
Graph of the boosting function $f$

Notice that there is no need to define $f(x)$ for $x \ge \overline{x}$ because by Assumption 4.6.1. no boosting occurs at such immune levels. The graph of $f$ is depicted in Fig. 3.

In our bookkeeping we incorporate the jump in the immune level resulting from boosting as the death of an individual with state $x$ and the simultaneous birth of an individual with state $f(x)$. Since $f((0, \overline{x}))\subset [\overline{x}, x_{m}]$ the set of possible states at birth is $\Omega _{0}:=[\overline{x}, x_{m}]$.

Our model is very similar to the one presented in [17] (see also [44]), but it has some differences. In [17] it is assumed that $\mathscr {L}(x)=\Lambda $ for any $x \in \Omega $, moreover the boosting function is more general than the one we have here. In our case, instead, we ‘massage’ the (assumptions about the) model ingredients such that there exists a renewal point: $\overline{x}$.

From the individual level mechanisms we construct the kernel as was done in Sect. 4.1 for the cell population model. Since waning is deterministic, an individual with immunity level $\xi $ that escapes boosting in a time interval of length $a$ will have, after time $a $, immunity level equal to $X(a, \xi ):=x(a)$ where $x$ solves the ODE (4.2), where $g $ is the waning rate. The survival probability, that is, the probability that an individual with immune level $\xi $ escapes boosting in the time interval of length $a$, is

$$ \mathscr {F}(a, \xi ) = e^{- \int _{0}^{a} \mathscr {L} (X(s, \xi )) ds}. $$

As in Sect. 4.1 we deduce that $k$ is equal to

4.8 $$ k(a, \xi ,\omega ) := \mathscr {L}(X(a, \xi ))\mathscr {F}(a, \xi ) \delta _{f(X(a, \xi ))}(\omega ) \quad a \in \mathbb{R}_{+} \times \Omega _{0} \times \mathscr {B}(\Omega _{0}); $$

which is a special case of (4.4) with $\gamma = \mathscr {L}$ and $\eta (x,\cdot ) =\delta _{f(x)}$.

#### Proposition 4.7

*The function* $k$ *defined by* (4.8) *is a factorisable* $z_{0}$-*kernel with* $z_{0} =-\Lambda $. *Its components are*

4.9 $$ M(t,\omega )= \Lambda \mathscr {F} (t, \overline{x}) \delta _{f(X(t, \overline{x}))} (\omega ), $$

4.10 $$ L(t,\xi )= H(t-\tau (\xi ,\overline{x})). $$

*Let* $\|\cdot \|=\|\cdot \|_{TV}$ *or* $\|\cdot \|=\|\cdot \|_{\flat }$. *If* $0 < \inf _{ x \in \Omega } \left | \frac{1}{g(x) } \right |< \infty $, *then*

$$ \| b(t, \cdot ) - D \hat{M}(0,\cdot )\| \rightarrow 0 \textit{ as } t \rightarrow \infty $$

The main difference between the model considered here and the one considered in [3] and [4] is that we do not explicitly divide the population into infected and susceptible individuals. As a consequence, we assume that the boosting does not depend on the number of infected individuals present in the population and we focus only on the dynamics of the immune levels without investigating the dynamics of the disease in the population.

Moreover, unlike in [13], we consider a continuum set of immunity levels.

## Proof of the Main and Some Auxiliary Results

To prove the results presented in Sect. 3 we proceed as follows:

1. In Sect. 5.1 we prove that equation (3.1) has a unique solution which can be expressed as a function of the solution of the one dimensional renewal equation (3.6);
2. In Sect. 5.2 we study the asymptotic behaviour of the solution of the reduced renewal equation via Feller’s Renewal Theorem;
3. In Sect. 5.3 we combine (3.6) with the results of Sect. 5.2 to deduce the asymptotic behaviour of the cumulative population birth rate;
4. In Sect. 5.4 we employ an additional regularity assumption to deduce the asymptotic behaviour of the population birth rate. We conclude the section by presenting a class of kernel components satisfying the additional regularity assumption.

### Existence of a Unique Measure-Valued Solution of the Renewal Equation and the Reduction of the Equation

In this section we prove the results stated in Sects. 3.1 and 3.2.

We shall show that (3.1) admits a unique solution by applying Theorem A.21 of the Appendix. As this theorem requires the kernel to be locally bounded we start by proving that factorisable $z_{0}$-Laplace kernels are indeed locally bounded

#### Proposition 5.1

*Let* $M$ *and* $L$ *be two* $z_{0}$-*Laplace components*. *Then the corresponding* $z_{0}$-*Laplace kernel defined by*

$$ k(a, \xi , \omega ):= \int _{[0, a]} \mu _{L}(d\sigma , \xi ) M(a- \sigma , \omega ) $$

*is locally bounded*.

#### Proof

For $(a, \xi , \omega ) \in \mathbb{R}_{+} \times \Omega _{0} \times \mathscr {B}(\Omega _{0})$ one has

$$\begin{aligned} k(a, \xi , \omega )&= \int _{[0, a]} \mu _{L}(d\sigma , \xi ) M(a- \sigma , \omega ) \leq \sup _{t \in \mathbb{R}_{+} } M(t,\Omega _{0}) \int _{[0,a]} \mu _{L}(d\sigma , \xi ) \\ & \leq \sup _{t \in \mathbb{R}_{+} } M(t,\Omega _{0}) L(a, \xi ). \end{aligned}$$

By the boundedness property included in the definition of a Laplace function component $L$ one has

$$ \sup _{(a,\xi ) \in \mathbb{R}_{+} \times \Omega _{0}} k(a, \xi , \omega ) < \infty , $$

for all $\omega \in \mathscr {B}(\Omega _{0})$. Moreover, the function $k(\cdot , \cdot , \omega ) $ is measurable for every $\omega \in \mathscr {B}(\Omega _{0})$ and

$$\begin{aligned} \sup _{\xi \in \Omega _{0}} \int _{0}^{\infty }e^{- z_{0} a} k(a, \xi , \Omega _{0}) da = \sup _{\xi \in \Omega _{0}} \int _{0}^{\infty }e^{- z_{0} \sigma } \mu _{L}(d\sigma , \xi ) \int _{0}^{\infty }e^{- z_{0} \alpha } M(\alpha ,\Omega _{0}) d\alpha < \infty . \end{aligned}$$

This completes the proof. □

#### Proof of Theorem 3.4

Since $f_{0}(\cdot , \Omega _{0})$ is locally bounded and, as shown in Proposition 5.1, $k $ is a locally bounded kernel, the conclusion follows from Theorem A.21. □

#### Proof of Proposition 3.6

The cumulative number $B$ of renewal events is a monotonically increasing function of time because $L$ is non-decreasing. Therefore $B $ is locally of bounded variation. By definition $B(0)=0$. We can apply the monotone convergence theorem to prove that $B$ is right continuous on $\mathbb{R}_{+}^{*}$. Indeed, recalling that $H$ is the Heaviside function, we have that

$$\begin{aligned} \lim _{h \rightarrow 0^{+}}B(t+h)&=\lim _{h \rightarrow 0^{+}}\int _{0}^{t+h} \int _{\Omega _{0}} b(\sigma ,d\xi ) L(t+h-\sigma ,\xi ) d\sigma \\ &= \lim _{h \rightarrow 0^{+}}\int _{0}^{\infty } \int _{\Omega _{0}} b( \sigma ,d\xi ) L(t+h-\sigma ,\xi )H(t+h-\sigma ) d\sigma \\ &=\int _{0}^{\infty } \int _{\Omega _{0}} b(\sigma ,d\xi ) \lim _{h \rightarrow 0^{+}} L(t+h-\sigma ,\xi )H(t+h-\sigma ) d\sigma =B(t). \end{aligned}$$

 □

#### Proof of Proposition 3.7

That equation (3.5) has a unique solution can be proven by an easy adaptation of the proof of [31, Theorem 3.1 p. 43] and [27, Theorem 1 pp. 185-186]. We therefore only need to show that $B$ solves (3.5). By the definition (2.4) of $K$ one has

$$\begin{aligned} \int _{0}^{t} \mu _{B}(ds) K(t-s) &= \int _{0}^{t} \mu _{B}(ds) \int _{0}^{t-s} \int _{\Omega _{0}} M(v,d\xi ) L(t-s-v,\xi ) dv \\ & = \int _{0}^{t} \mu _{B}(ds) \int _{s}^{t} \int _{\Omega _{0}} M( \sigma -s,d\xi ) L(t-\sigma ,\xi ) d\sigma \\ &= \int _{0}^{t} \int _{0}^{\sigma }\mu _{B}(ds) \int _{\Omega _{0}} M( \sigma -s,d\xi ) L(t-\sigma ,\xi ) d\sigma . \end{aligned}$$

Here the last equality follows from the Fubini-Tonelli theorem, which can be applied because the function $(s, \sigma ) \mapsto \int _{\Omega _{0}} M(\sigma -s,d\xi ) L(t- \sigma ,\xi )$ is bounded and measurable. From equation (3.8) and Theorem 5.2, applied to the functions $s \mapsto B(s) $ and $s \mapsto M(s,\omega )$, it follows that

$$\begin{aligned} &\int _{0}^{t} \int _{0}^{\sigma }\mu _{B}(ds ) M(\sigma -s, \omega ) d \sigma = \int _{0}^{t} \frac{d}{d\sigma } \int _{0}^{\sigma }B(s ) M( \sigma -s, \omega ) ds d\sigma \\ & = \int _{0}^{t} B(s ) M(t-s, \omega ) ds = \tilde{b}( [0,t] , \omega ) - \tilde{f_{0}}([0,t], \omega ). \end{aligned}$$

Therefore, for any Borel set $A \in \mathscr {B} (\mathbb{R}_{+})$

$$ \int _{A} \int _{0}^{\sigma }\mu _{B}(ds ) M(\sigma -s, \omega ) d \sigma = \tilde{b}(A, \omega ) - \tilde{f_{0}}(A , \omega ). $$

Now recall from equation (3.8), that $b(\cdot ,\omega )$ and $f_{0}(\cdot ,\omega )$ are, by definition, the densities corresponding to the measures $\tilde{b}(\cdot ,\omega )$ and $\tilde{f}_{0}(\cdot ,\omega )$. Hence for any rectangle $A \times C =R\in \mathscr {P}$, with $\mathscr {P}$ defined as (A.10), it holds that

$$\begin{aligned} &\int _{0}^{t} \int _{0}^{\sigma }\mu _{B}(ds) \int _{\Omega _{0}}M( \sigma -s,d\xi ) \chi _{A}(t-\sigma ) \chi _{C}(\xi ) d\sigma \\ & = \int _{0}^{t} \int _{\Omega _{0}} b(\sigma , d\xi ) \chi _{A}(t- \sigma ) \chi _{C} (\xi ) d\sigma - \int _{0}^{t} \int _{\Omega _{0}} f_{0}( \sigma , d\xi ) \chi _{A}(t-\sigma ) \chi _{C} (\xi ) d\sigma . \end{aligned}$$

Indeed, denoting $A_{t} = \{ v \in [0,t] : v= t-a \text{ with } a \in A\}$, we can write

$$\begin{aligned} &\int _{0}^{t} \int _{0}^{\sigma }\mu _{B}(ds) \int _{\Omega _{0}} M( \sigma -s,d\xi ) \chi _{A}(t-\sigma ) \chi _{C}(\xi ) d\sigma \\ & = \int _{0}^{t} \int _{0}^{\sigma }\mu _{B}(ds) M(\sigma -s,C) \chi _{A_{t}}(\sigma ) d \sigma \\ & = \int _{A_{t}} \int _{0}^{\sigma }\mu _{B}(ds) M(\sigma -s,C) d \sigma = \tilde{b}(A_{t}, C) - \tilde{f_{0}}(A_{t}, C) \\ & = \int _{0}^{t} \int _{\Omega _{0}} b (\sigma , d\xi ) \chi _{A}(t- \sigma ) \chi _{C}(\xi ) d\sigma - \int _{0}^{t} \int _{\Omega _{0}} f_{0} (\sigma , d\xi ) \chi _{A}(t-\sigma ) \chi _{C}(\xi ) d\sigma . \end{aligned}$$

We aim at proving that

5.1 $$\begin{aligned} &\int _{0}^{t} \int _{0}^{\sigma }\mu _{B}(ds) \int _{\Omega _{0}}M( \sigma -s,d\xi ) \chi _{A}(t-\sigma , \xi ) d\sigma \\ & = \int _{0}^{t} \int _{\Omega _{0}} b(\sigma , d\xi ) \chi _{A}(t- \sigma , \xi ) d\sigma - \int _{0}^{t} \int _{\Omega _{0}} f_{0}( \sigma , d\xi ) \chi _{A}(t-\sigma , \xi ) d\sigma \end{aligned}$$

holds for any $A \in \mathscr {B}(\mathbb{R}_{+} \times \Omega _{0} ) $. We proceed as in the proof of Lemma A.12 and define

$$ \mathscr {E}:= \left \{ A \subset \mathbb{R}_{+} \times \Omega _{0} : \text{such that } \text{(5.1)} \text{ holds} \right \} . $$

Notice that any set in $\mathscr {P} $ satisfies (5.1) and therefore $\mathscr {P} \subset \mathscr {E}$. The collection of sets ℰ is a Dynkin system. Indeed $\mathbb{R}_{+} \times \Omega _{0} \in \mathscr {E}$, since

$$\begin{aligned} &\int _{0}^{t} \int _{0}^{\sigma }\mu _{B}(ds) \int _{\Omega _{0}} M ( \sigma -s,d\xi ) \chi _{\mathbb{R}_{+} \times \Omega _{0}}(t-\sigma , \xi ) d\sigma \\ & = \int _{0}^{t} \int _{0}^{\sigma }\mu _{B}(ds) M(\sigma -s, \Omega _{0}) d\sigma \\ & = \int _{0}^{t} b (\sigma , \Omega _{0}) d\sigma - \int _{0}^{t} f_{0} (\sigma , \Omega _{0}) d\sigma \\ & =\int _{0}^{t} \int _{\Omega _{0}} b (\sigma , d\xi ) \chi _{ \mathbb{R}_{+} \times \Omega _{0}}(t-\sigma , \xi ) d\sigma - \int _{0}^{t} \int _{\Omega _{0}} f_{0} (\sigma , d\xi ) \chi _{\mathbb{R}_{+} \times \Omega _{0}}(t-\sigma , \xi ) d\sigma . \end{aligned}$$

If $A \in \mathscr {E}$, then $A^{c} \in \mathscr {E}$ thanks to the fact that $\chi _{A^{c}}=1-\chi _{A} $. If $A=\bigcup _{n=1}^{\infty }A_{n} $, with $\{A_{n} \} \subset \mathscr {E} $ a sequence of pairwise disjoint sets, then $A \in \mathscr {E}$ thanks to the fact that $\chi _{A}=\sum _{n} A_{n}$. Exactly as in Lemma A.12, since $\mathscr {P} \subset \mathscr {E}$ we conclude by Dynkin’s Lemma that $\mathscr {B}(\mathbb{R}_{+} \times \Omega _{0})= \mathscr {B}(\mathbb{R}_{+}) \otimes \mathscr {B}(\Omega _{0}) \subset \mathscr {E}$ and we conclude that (5.1) holds for any $A\in \mathscr {B}(\mathbb{R}_{+} \times \Omega _{0})$.

Therefore, the functions

$$ \varphi _{n} (\sigma , \xi )= \sum _{i=1}^{n} \alpha ^{n}_{i} \chi _{C^{n}_{i}}( \sigma , \xi ), \quad C^{n}_{i} \in \mathscr {B}(\mathbb{R}_{+} \times \Omega _{0}), \quad \alpha ^{n}_{i} \geq 0 $$

approximating $M $ satisfy

$$\begin{aligned} &\int _{0}^{t} \int _{0}^{\sigma }\mu _{B}(ds) \int _{\Omega _{0}}M( \sigma -s,d\xi ) \varphi _{n}(t-\sigma , \xi ) d\sigma = \int _{0}^{t} \int _{\Omega _{0}} b(\sigma , d\xi ) \varphi _{n}(t-\sigma , \xi ) d \sigma \\ & - \int _{0}^{t} \int _{\Omega _{0}} f_{0}(\sigma , d\xi ) \varphi _{n} (t-\sigma , \xi ) d\sigma . \end{aligned}$$

Passing to the limit we obtain

$$\begin{aligned} \int _{0}^{t} \int _{0}^{\sigma }\mu _{B}(ds) \int _{\Omega _{0}}M( \sigma -s,d\xi ) L(t-\sigma ,\xi ) d\sigma =& \int _{0}^{t} \int _{ \Omega _{0}} b(\sigma , d\xi ) L(t-\sigma , \xi ) d\sigma - z(t) \end{aligned}$$

and the conclusion follows. □

#### Proof of Lemma 3.8

From (3.4) and the defining properties of the function component $L $ and the forcing function $f_{0}$ we find that

$$\begin{aligned} z_{\infty }&= \lim _{t \rightarrow \infty } \int _{0}^{t}\! \int _{\Omega _{0}} f_{0}(t-\sigma ,d\xi ) L(\sigma ,\xi ) d\sigma = \lim _{t \rightarrow \infty } \int _{0}^{\infty }\!\int _{\Omega _{0}} f_{0}( \sigma ,d\xi ) L(t-\sigma ,\xi ) H(t-\sigma ) d\sigma \\ &\leq \int _{0}^{\infty }f_{0}(\sigma , \Omega _{0}) d \sigma \sup _{( \tau , \xi ) \in \mathbb{R}_{+} \times \Omega _{0} } L(\tau , \xi ) < \infty . \end{aligned}$$

This proves statement 1.

By the definition (2.4) of $K$ that for any integrable function $f$ one has

5.2 $$ \int _{0}^{\infty }f(s) \mu _{K}(ds) =\int _{0}^{\infty }\int _{\Omega _{0}} M(\tau , d\xi )\int _{0}^{\infty }f(\tau +\eta ) \mu _{L}(d\eta , \xi ). $$

The identity (5.2) can be proved by approximating the measurable function $f$ from below and using the definition of integral with respect to a measure and the dominated convergence theorem. We omit the details as the proof is similar to the one of Lemma A.14.

Assume next that $w > z_{0}$. Then, using (5.2) we get

$$\begin{aligned} & \int _{0}^{\infty }s e^{-ws} \mu _{K}(ds) = \int _{0}^{\infty }\int _{ \Omega _{0}} M(\tau , d\xi ) \int _{0}^{\infty }\left ( \eta + \tau \right ) e^{-w( \eta + \tau )} \mu _{L}(d\eta , \xi ) d \tau \\ &= \int _{0}^{\infty }\int _{\Omega _{0}}e^{-w\tau } M(\tau , d\xi ) d \tau \int _{0}^{\infty }\eta e^{-w\eta }\mu _{L}(d\eta , \xi ) \\ &+ \int _{0}^{\infty }\int _{\Omega _{0}}\tau e^{-w \tau } M(\tau , d \xi ) d\tau \int _{0}^{\infty }e^{-w \eta } \mu _{L}(d\eta , \xi ) \\ & \leq \int _{0}^{\infty }e^{-w \tau } M(\tau , \Omega _{0}) d\tau \sup _{ \xi \in \Omega _{0}} \int _{0}^{\infty }\eta e^{(z_{0}-w)\eta } e^{- z_{0} \eta } \mu _{L}(d\eta , \xi ) \\ &+ \int _{0}^{\infty }\tau e^{(z_{0}-w) \tau } e^{- z_{0} \tau } M(\tau , \Omega _{0}) d\tau \sup _{ \xi \in \Omega _{0}} \int _{0}^{\infty }e^{-w \eta } \mu _{L}(d\eta , \xi ) \\ & \leq \frac{1}{w- z_{0}} e^{-1} \left ( \hat{M}(z_{0}, \Omega _{0}) + \sup _{ \xi \in \Omega _{0}} \hat{ \mu _{L}} (z_{0}, \xi ) \right ) < \infty \end{aligned}$$

and statement 2. is proved.

Assume now that $z_{0}< w \leq 0$. Statement 3. follows because by (3.4)

$$\begin{aligned} \left | z(t) - z_{\infty }\right | &\leq \left | \int _{0}^{t} \int _{ \Omega _{0}} f_{0}(\sigma , d\xi ) L(t-\sigma , \xi ) - \int _{0}^{\infty }\int _{\Omega _{0}} f_{0}(\sigma , d\xi ) L(t-\sigma , \xi ) \right | \\ & \leq \int _{t}^{\infty }f_{0}(\sigma , \Omega _{0}) d\sigma \sup _{(s, \xi )\in \mathbb{R}_{+} \times \Omega _{0}} L(s, \xi ) \\ & \leq e^{ z_{0} t } \int _{0}^{\infty }f_{0}(\sigma , \Omega _{0}) e^{- z_{0} \sigma } d \sigma \sup _{(s, \xi )\in \mathbb{R}_{+} \times \Omega _{0}} L(s, \xi ). \end{aligned}$$

Finally, if $w>0$, statement 4. follows from $z_{\infty }< \infty $. □

We now turn our attention to the one dimensional representation. The proof of Theorem 3.9 is just an integral manipulation if $L(\cdot ,\xi )$ is a differentiable function. Since in our examples we deal, instead, with a not-necessarily differentiable bounded variation function of the form (2.2), we use the definition of weak derivative of a NBV function given in Sect. A.2 to prove Theorem 3.9.

Theorem 5.2 below, which can be found e.g. in the book by Gripenberg *& al.* [31, Corollary 7.3(ii)], will be very useful when we deal with derivatives of convolution products. To state it we need some notation.

If $a$ and $b$ are Lebesgue measurable functions on $\mathbb{R}_{+}$ we use the notation $a \star _{1} b$ for

$$ (a \star _{1} b)(t):=\int _{0}^{t} a(t-\sigma )b(\sigma ) d\sigma . $$

If $b$ is a Lebesgue measurable function and $\mu \in M(\mathbb{R}_{+})$ we use the notation $\mu \star _{2} b$ for

$$ (\mu \star _{2} b)(t):=\int _{0}^{t} b(t-\sigma )\mu (d\sigma ). $$

#### Theorem 5.2

*Let* $a \in BV_{\mathrm{loc}}(\mathbb{R}_{+})$ *and* $b \in L^{p}_{\mathrm{loc}}(\mathbb{R}_{+})$ *for some* $p \in [1,\infty ]$. *Then* $(a \star _{1} b)$ *is absolutely continuous and* $(a\star _{1} b)'=\mu \star _{2} b $ *where* $\mu $ *is the measure induced by the distributional derivative of* $a$. *In particular*, $(a \star _{1} b)' \in L^{p}_{\mathrm{loc}}(\mathbb{R}_{+})$.

#### Proof of Theorem 3.9

Because $b$ satisfies (3.1), we have

$$\begin{aligned} \int _{0}^{t} b(\tau ,\omega )d\tau &=\int _{0}^{t} \left ( \int _{0}^{\tau }\int _{\Omega _{0}} b(\sigma ,d\xi ) k(\tau -\sigma ,\xi , \omega ) d\sigma + f_{0}(\tau ,\omega ) \right ) d\tau \\ & = \int _{0}^{t}\int _{\Omega _{0}} b(\sigma ,d\xi ) \int _{\sigma }^{t} k(\tau -\sigma ,\xi ,\omega ) d\tau d\sigma + \int _{0}^{t} f_{0}( \tau , \omega ) d\tau \\ & = \int _{0}^{t}\int _{\Omega _{0}} b(\sigma ,d\xi ) \int _{0}^{t- \sigma } k(v ,\xi ,\omega ) dv d\sigma + \int _{0}^{t} f_{0}(\tau , \omega ) d\tau . \end{aligned}$$

Fix $(\xi ,\omega ) \in \Omega _{0} \times \mathscr {B}(\Omega _{0}) $ and apply Theorem 5.2 to the maps $\sigma \mapsto L(\sigma ,\xi )$ and $\sigma \mapsto M(\sigma ,\omega )$. This yields

$$\begin{aligned} \frac{d }{dv }\left (\int _{0}^{v} L(\sigma ,\xi ) M(v-\sigma , \omega ) d\sigma \right ) &= \int _{0 }^{v} \mu _{L}(d \sigma ,\xi ) M(v- \sigma , \omega ) =k(v,\xi ,\omega ). \end{aligned}$$

Therefore,

$$\begin{aligned} \int _{0}^{t-\sigma } k(v,\xi ,\omega ) dv &=\int _{0}^{t - \sigma } \frac{d}{dv} \left (\int _{0}^{v} L(s,\xi ) M(v-s, \omega ) ds \right ) dv \\ & =\int _{0}^{t-\sigma } L(s,\xi ) M(t-\sigma -s, \omega )ds. \end{aligned}$$

Consequently,

$$\begin{aligned} \int _{0}^{t} b(\tau ,\omega )d\tau - \int _{0}^{t}f_{0}(\tau , \omega ) d\tau &= \int _{0}^{t}\int _{\Omega _{0}} b(\sigma ,d\xi ) \int _{0}^{t-\sigma } L(s ,\xi ) M(t-\sigma -s, \omega ) ds d\sigma \\ &= \int _{0}^{t} \int _{0 }^{t-\sigma } \int _{\Omega _{0}} b(\sigma ,d \xi ) L(s,\xi ) M(t-\sigma -s,\omega ) ds d\sigma \\ &= \int _{0}^{t} \int _{0 }^{t-\sigma } \int _{\Omega _{0}} b(\sigma ,d \xi ) L(t-\sigma -v,\xi ) M(v,\omega ) dv d\sigma \\ &= \int _{0}^{t} \int _{0 }^{t-v} \int _{\Omega _{0}} b(\sigma ,d\xi ) L(t-\sigma -v,\xi ) d\sigma M(v,\omega ) dv \\ &=\int _{0}^{t} B(t-\sigma )M(\sigma , \omega ) d\sigma , \end{aligned}$$

where in the last equality we used the definition (3.3) of $B$. □

### Asymptotic Behaviour of the Reduced Equation

In this section we prove Lemma 3.8 and Theorem 3.13. We shall apply Feller’s Renewal Theory [27] to (3.5) and we therefore start by introducing the definition of direct integrability, which is important for the theory.

#### Definition 5.3

Direct Riemann integrability

A non-negative function $f : \mathbb{R}_{+} \rightarrow \mathbb{R}_{+}$ is called directly Riemann integrable if the upper and lower Riemann sums over the partition $0 < h < 2h < \cdots < (n-1)h < nh < \cdots $ are finite and tend to the same limit as $h \rightarrow 0$.

Every directly Riemann integrable function is in $L^{1}({\mathbb{R}})$ (with respect to Lebesgue measure) but the converse need not be true (counterexample: an $L^{1}$-function oscillating between 0 and 1 at infinity). However, the functions we will work with are either monotone functions or products of monotone functions and for them Lebesgue integrability is equivalent to direct Riemann integrability.

As detailed below, the asymptotic behaviour of the solution of the reduced renewal equation (3.5) follows from the renewal theorems proven by Feller in [27, Volume 2, Chap. 11, Sects. 1 and 6]. In the book [27] there are many useful versions of the theorem, below we summarise the ones that will be used in the proof of Theorem 3.13, viz. Renewal Theorem (first form) [27, p. 360], Renewal Theorem (alternative form) [27, p. 363] and Theorem 2 [27, p. 376]).

Feller’s renewal theorem is concerned with the asymptotic behaviour of solutions of the scalar renewal equation

5.3 $$ X(t) = \int _{0}^{t} X(t-s) \mu _{G}(ds) + y(t), $$

where $G\in NBV(\mathbb{R}_{+})$ and $y : \mathbb{R}_{+} \rightarrow \mathbb{R}_{+} $ is a bounded function.

Let $R$ be the resolvent of $G$. Recall from the appendix that $R$ is the unique solution of (A.6) and that the unique solution of (5.3) is given by the formula (A.7).

#### Theorem 5.4

Renewal Theorem

*Let* $G\in NBV(\mathbb{R}_{+})$ *be non*-*decreasing and non*-*arithmetic*, *with* $\int _{0}^{\infty }s \mu _{G}(ds) < \infty $ *and let* $R$ *be the resolvent of* $G$.

1. *If* $\lim _{t \rightarrow \infty } G(t)= \int _{0}^{\infty }\mu _{G}(ds) =1$, *then for every* $h>0$
  5.4 $$ R(t ) - R(t-h) \rightarrow \frac{h}{\int _{0}^{\infty }s \mu _{G}(ds) } \textit{ as } t \rightarrow \infty . $$
  *Let* $y$ *in* (5.3) *be directly Riemann integrable*. *Then the solution* $X$ *of* (5.3) *satisfies*
  5.5 $$ X(t) \rightarrow \frac{ \int _{0}^{\infty }y(s) ds }{ \int _{0}^{\infty }s \mu _{G}(ds) } \quad \textit{ as } t \rightarrow \infty . $$
2. *Assume that the limit* $y_{\infty }:=\lim _{t \to \infty } y(t)$ *exists*. *If* $\int _{0}^{\infty }\mu _{G}(ds) <1$, *if there exists a number* $r<0$ *such that* $\int _{0}^{\infty }e^{-r s} \mu _{G}(ds) =1$ *and if the function* $y_{\infty }- y(t) + y_{\infty }\frac{ \int _{0}^{\infty }\mu _{G}(ds) - \int _{0}^{t} \mu _{G}(ds) }{1- \int _{0}^{\infty }\mu _{G}(ds) } $ *is directly Riemann integrable*, *then*
  5.6 $$ e^{-r t} \left (\lim _{v \rightarrow \infty } X(v)- X(t) \right ) \int _{0}^{\infty }e^{-r s }s \mu _{G}(ds) \rightarrow \frac{ y_{\infty }}{-r}+ \int _{0}^{\infty }e^{-r s} \left (y_{\infty }- y(s) \right ) ds $$
  *as* $t$ *goes to infinity*, *provided that* $\int _{0}^{\infty }e^{-r s } s \mu _{G}(ds) < \infty $.

The proof of Theorem 3.13 is based on the Renewal Theorem 5.4. The main task is therefore to check that the assumptions of the Renewal Theorem hold for the renewal equation (3.5).

#### Proof of Theorem 3.13

Equation (3.5) can be written in the form

$$ B(t) = \int _{0}^{t} B(t-s) \mu _{K}(ds) + z(t). $$

Indeed

$$\begin{aligned} \int _{0}^{t} K(t-s) \mu _{B}(ds) &= \int _{0}^{t} \tilde{K}_{t}(s) \mu _{B}(ds) = B(t) K(0)- B(0) K(t) - \int _{0}^{t} B(s) \mu _{ \tilde{K}_{t}}(ds) \\ &= - \int _{0}^{t} B(s) \mu _{\tilde{K}_{t}}(ds) \end{aligned}$$

where, $\tilde{K}_{t}(s):=K(t-s)$ and the integration by parts for Lebesgue-Stieltjes integrals is used, see [37] for details. The measure $\mu _{\tilde{K}_{t}} $ can be seen as the pushforward measure of $\mu _{K}$ with respect to the left translation function. Therefore we can apply the change of variables formula to obtain that

$$\begin{aligned} - \int _{0}^{t} B(s) \mu _{\tilde{K}_{t}}(ds) = \int _{0}^{t} B(t-s) \mu _{K}(ds). \end{aligned}$$

Notice that $z(t)$ is an increasing function, therefore $z(t)$ is not integrable, but thanks to Lemma 3.8 we know that $z_{\infty }< \infty $.

**Case 1:** $r>0$.

We shall apply the alternative version of the Renewal Theorem to the rescaled equation

$$ B_{\#} (t) = \int _{0}^{t} B_{\#}(t-s) \mu _{K_{\#}}(ds) + z_{\#}(t) $$

with

$$ B_{\#} (t) := e^{-rt } B(t), \quad z_{\#}(t):= e^{-rt} z(t), \quad K_{ \#}(t) = \int _{0}^{t} e^{-rs} \mu _{K}(ds). $$

Notice that $K_{\#}(0)=0$, $K_{\#} $ is increasing and right-continuous, on the other hand $z_{\#}$ is integrable, indeed

$$\begin{aligned} \int _{0}^{\infty }z_{\#}(t) dt &=\int _{0}^{\infty }e^{-rt} z(t) dt = \int _{0}^{\infty }e^{- rt } \int _{0}^{t} \int _{\Omega _{0}} f_{0}( \sigma , d\xi ) M(t-\sigma , \xi ) d\sigma dt \\ & \leq \int _{0}^{\infty }e^{-rt} dt \int _{0}^{\infty }f_{0}(\sigma , \Omega _{0}) d\sigma \sup _{(s, \xi ) \in \mathbb{R}_{+} \times \Omega _{0} } M(s, \xi ) < \infty . \end{aligned}$$

Therefore, the hypothesis of Theorem 5.4, point 1, holds and we conclude that

$$ B_{\#}(t) \rightarrow \frac{\int _{0}^{\infty }z(s) e^{-rs} ds }{\int _{0}^{\infty }s e^{-rs}\mu _{K}(ds)} \quad t \rightarrow \infty . $$

Since $B(t)=e^{rt} B_{\#}(t)$, we obtain the statement of the theorem.

**Case 2:** $r<0$.

By Lemma 3.8 we know that $z_{\infty }< \infty $. It is also true that $B_{\infty }< \infty $. Indeed, if $R$ denotes the resolvent of the kernel $K$,

$$\begin{aligned} B_{\infty }&= \lim _{t \rightarrow \infty } B(t) = \lim _{t \rightarrow \infty } \int _{0}^{t} z(t-s) \mu _{R} (ds) + z(t) \leq z_{\infty }\sup _{ t \in \mathbb{R}_{+}} R(t) + z_{\infty }< \infty . \end{aligned}$$

The fact that $\sup _{ t \in \mathbb{R}_{+}} R(t) < \infty $, follows by the fact that $r<0$, and consequently $\int _{0}^{\infty }\mu _{K} (ds) < 1 $, combined with the definition of the resolvent.

We shall apply Theorem 5.4 point 2. The only hypothesis we need to check is that

$$ z_{\infty }- z(t) + z_{\infty } \frac{\int _{t}^{\infty }\mu _{K}(dt) }{1-\int _{0}^{\infty }\mu _{K}(dt) } $$

is integrable. We already know that $z_{\infty }- z(t)$ is integrable, we just need to verify that $\int _{t}^{\infty }\mu _{K}(ds) $ is integrable. From Lemma 3.8 we deduce that

$$\begin{aligned} \int _{0}^{\infty }\int _{t}^{\infty }\mu _{K}(ds) dt = \int _{0}^{\infty }\int _{0}^{s} dt \mu _{K}(ds) = \int _{0}^{\infty }s \mu _{K}(ds) < \infty . \end{aligned}$$

Therefore we can apply the Theorem 5.4 and obtain the conclusion.

**Case 3:** $r=0$.

Thanks to Lemma 3.8, the function $z(t) -z_{\infty }$ is integrable. We denote by $\tilde{B} $ the solution of the renewal equation

$$ \tilde{B}(t) = \int _{0}^{t} \tilde{B}(t-s) \mu _{K}(ds) + z(t) - z_{\infty }. $$

By Theorem 5.4 point 1, we conclude that

$$ \tilde{B} (t) \rightarrow \frac{\int _{0}^{\infty }\left ( z(s) - z_{\infty }\right ) ds }{\int _{0}^{\infty }s d\mu _{K} (s) } \quad t \rightarrow \infty . $$

On the other hand, we notice that the solution of equation (3.5) is given by

$$ B(t)= \tilde{B}(t)+ \tilde{B}_{\infty }(t) $$

where

$$ \tilde{B}_{\infty }(t) = \int _{0}^{t} \tilde{B}_{\infty }(t-s) \mu _{K}(ds) + z_{\infty }. $$

By the theory of renewal equations we know that

$$ \tilde{B}_{\infty }(t) = \int _{0}^{t} z_{\infty }\mu _{R}(ds) + z_{\infty }=z_{\infty }R(t) + z_{\infty }, $$

where $R$ is the resolvent of the equation and where we have used

$$ \int _{0}^{t} \mu _{R}(ds) = R(t)-R(0)=R(t). $$

We deduce the asymptotic behaviour of the resolvent applying the Renewal Theorem. We obtain

$$ R(t) \sim \frac{t}{\int _{0}^{\infty }s \mu _{K}(ds)} \quad t \rightarrow \infty . $$

From this we deduce

$$ \tilde{B}_{\infty }(t) \sim t \cdot \frac{z_{\infty }}{\int _{0}^{\infty }s \mu _{K}(ds) } \quad t \rightarrow \infty $$

and, therefore,

$$ B(t) \sim t \cdot \frac{z_{\infty }}{\int _{0}^{\infty }s \mu _{K}(ds) } \quad t \rightarrow \infty . $$

 □

### Behaviour of the Cumulative Population Birth Rate

The proof of Theorem 3.14 seems complicated at first sight, but it is in fact elementary. The main idea is to combine (3.6) with Theorem 3.13 and the fact that $M$ is a Laplace component while $f_{0}$ is a forcing function.

#### Proof of Theorem 3.14

**Case 1:** $r>0$.

We prove that, for every $\omega \in \mathscr {B}(\Omega _{0})$,

$$ \int _{0}^{t} B(\tau ) M(t- \tau , \omega ) d\tau \sim \int _{0}^{t} e^{r \tau }M(t- \tau , \omega ) d\tau \quad \text{ for } t \rightarrow \infty . $$

We notice that, since $M$ is a Laplace component, one has

5.7 $$\begin{aligned} \begin{aligned} \int _{0}^{t} e^{r \tau } M(t-\tau , \omega ) d\tau &= \int _{0}^{t} e^{(t- \sigma ) r } M(\sigma , \omega ) d\sigma \\ &= e^{ t r } \int _{0}^{t} e^{- \sigma r } M(\sigma , \omega ) d\sigma \rightarrow \infty \quad \text{ as } t \rightarrow \infty . \end{aligned} \end{aligned}$$

By Theorem 3.13, we know that $B(t) \sim e^{ r t } C_{r} $ for $t \rightarrow \infty $, thus for every $\varepsilon >0$ there exists a $T_{\varepsilon }>0$ such that for every $t > T_{\varepsilon }$, we have

$$ \left |B(t) - C_{r} e^{r t} \right | < \varepsilon C_{r} e^{ rt}. $$

Given any $\omega \in \mathscr {B}(\Omega _{0})$ we can conclude that, for every $\varepsilon >0$ there exists $T_{\varepsilon }$ such that

$$\begin{aligned} &\int _{0}^{t} \left | B(\tau )- C_{r} e^{ r \tau } \right | M(t-\tau , \omega ) d\tau = \int _{0}^{T_{\varepsilon }} \left | B(\tau )- C_{r} e^{ r \tau } \right | M(t-\tau , \omega ) d\tau \\ & + \int _{T_{\varepsilon }}^{t} \left | B(\tau )- C_{r} e^{ r \tau } \right | M(t-\tau , \omega ) d\tau \leq \max _{[0, T_{\varepsilon }]}{ \left | B(\tau )- C_{r} e^{ r \tau } \right | } \int _{0}^{T_{\varepsilon }} M(t-\tau , \omega ) d\tau \\ & + \varepsilon \int _{T_{\varepsilon }}^{t} C_{r} e^{ r \tau } M(t-\tau , \omega ) d\tau \leq \max _{[0, T_{\varepsilon }]}{\left | B(\tau )- C_{r} e^{ r \tau } \right | } e^{z_{0}(t- T_{\varepsilon })} \int _{0}^{T_{\varepsilon }} e^{- z_{0}(t- \tau )} M(t-\tau , \omega ) d\tau \\ & + \varepsilon C_{r} \int _{0}^{t} e^{ r \tau } M(t-\tau , \omega ) d \tau . \end{aligned}$$

Therefore,

$$\begin{aligned} & \left | \frac{ \int _{0}^{t} \left (B(\tau )- C_{r} e^{ r \tau } \right ) M(t-\tau , \omega ) d\tau }{ \int _{0}^{t} C_{r} e^{ r \tau } M(t-\tau , \omega ) d\tau } \right | \leq \frac{ \int _{0}^{t} \left | B(\tau )- C_{r} e^{ r \tau } \right | M(t-\tau , \omega ) d\tau }{ \int _{0}^{t} C_{r} e^{ r \tau } M(t-\tau , \omega ) d\tau } \\ & \leq \frac{ \max _{[0, T_{\varepsilon }]}{\left | B(\tau )- C_{r} e^{ r \tau } \right | } e^{ z_{0} t} e^{- z_{0} T_{\varepsilon }} \int _{0}^{T_{\varepsilon }} e^{- z_{0}(t- \tau )} M(t-\tau , \omega ) d\tau }{ \int _{0}^{t} C_{r} e^{ r \tau } M(t-\tau , \omega ) d\tau } + \varepsilon . \end{aligned}$$

From (5.7) we deduce that there exits a $\tilde{T}_{\varepsilon }>0$ such that for every $t >\tilde{T}_{\varepsilon }$

$$ \frac{ \max _{[0, T_{\varepsilon }]}{\left | B(\tau )- C_{r} e^{ r \tau } \right | } e^{ z_{0} t} e^{- z_{0} T_{\varepsilon }} \int _{0}^{T_{\varepsilon }} e^{- z_{0}(t- \tau )} M(t-\tau , \omega ) d\tau }{ \int _{0}^{t} C_{r} e^{ r \tau } M(t-\tau , \omega ) d\tau } < \varepsilon . $$

Therefore, for every $\varepsilon >0$, $\tilde{ T}_{\varepsilon }$ is such that, for every $t> \tilde{T}_{\varepsilon }$,

$$ \left | \frac{ \int _{0}^{t} \left ( B(\tau )- C_{r} e^{ r \tau } \right ) M(t-\tau , \omega ) d\tau }{ \int _{0}^{t} C_{r} e^{ r \tau } M(t-\tau , \omega ) d\tau } \right | < 2 \varepsilon $$

or equivalently,

$$ \int _{0}^{t} B(\tau ) M(t- \tau , \cdot ) d\tau \sim \int _{0}^{t} e^{r \tau } C_{r} M(t- \tau , \cdot ) d\tau $$

setwise as $t$ goes to infinity. Since $f_{0}$ is a forcing function

$$ \int _{0}^{\infty }f_{0}(\tau , \Omega _{0}) d\tau < \infty $$

it follows that for every $\omega \in \mathscr {B}( \Omega _{0})$,

$$\begin{aligned} \int _{0}^{t} b(\tau , \omega ) d \tau & = \int _{0}^{t} B(\tau ) M(t- \tau , \omega ) d\sigma + \int _{0}^{t} f_{0}(\tau , \omega ) d \sigma \sim e^{r t} C_{r} \int _{0}^{\infty }e^{ - r \tau }M(\tau , \omega ) d\tau \end{aligned}$$

as $t$ tends to infinity.

**Case 2:** $z_{0}< r<0$.

Since $M$ is a Laplace component we have for every $\omega \in \mathscr {B}(\Omega _{0})$

5.8 $$\begin{aligned} \int _{0}^{t} e^{ r\tau } M(t-\tau , \omega ) d\tau = e^{ r t } \int _{0}^{t} e^{- r\sigma } M(\sigma , \omega ) d\sigma \rightarrow 0 \quad \text{ as } t \rightarrow \infty . \end{aligned}$$

With the same argument as above, we obtain that for every $\varepsilon >0$, there exists a $T_{\varepsilon }>0$ such that for every $t > T_{\varepsilon }$,

$$\begin{aligned} & \left | \frac{ \int _{0}^{t} \left ( B(\tau )- K_{r} e^{ r \tau } - B_{\infty }\right ) M(t-\tau , \omega ) d\tau }{ \int _{0}^{t} \left ( K_{r} e^{ r \tau } + B_{\infty }\right ) M(t-\tau , \omega ) d\tau } \right | \leq \frac{ \int _{0}^{t} \left | B(\tau )- K_{r} e^{ r \tau }- B_{\infty }\right | M(t-\tau , \omega ) d\tau }{ \left | \int _{0}^{t} \left ( K_{r} e^{ r \tau } + B_{\infty }\right ) M(t-\tau , \omega ) d\tau \right | } \leq \\ & \leq \frac{ \max _{[0, T_{\varepsilon }]}{\left | B(\tau )- K_{r} e^{ r \tau }- B_{\infty }\right | } e^{ z_{0} t} e^{- z_{0} T_{\varepsilon }} \int _{0}^{T_{\varepsilon }} e^{- z_{0}(t- \tau )} M(t-\tau , \omega ) d\tau }{ \left | e^{ r t} \int _{0}^{t} \left (K_{r} e^{- r \sigma }+ B_{\infty }\right ) M(\sigma , \omega ) d\sigma \right | } + \varepsilon . \end{aligned}$$

Notice that

$$ 0 < \lim _{ t \rightarrow \infty }e^{ r t} \int _{0}^{t} \left | K_{r} e^{- r \sigma }+ B_{\infty }\right | M(\sigma , \omega ) d\sigma < \infty . $$

This together with (5.8), implies that for every $\varepsilon >0$ there exists a $t_{\varepsilon }>0 $ such that for every $t > t_{\varepsilon }$

$$ \left | \frac{ \int _{0}^{t} \left ( B(\tau )- K_{r} e^{ r \tau } - B_{\infty }\right ) M(t-\tau , \omega ) d\tau }{ \int _{0}^{t} \left ( K_{r} e^{ r \tau }+ B_{\infty }\right ) M(t-\tau , \omega ) d\tau } \right |< 2 \varepsilon , $$

which is equivalent to

5.9 $$ \int _{0}^{t} B(\tau ) M(t- \tau , \cdot ) d\tau \sim \int _{0}^{t} e^{r \tau } K_{r} M(t- \tau , \cdot ) d\tau + B_{\infty }\hat{M}(0,\cdot ) \quad \text{ setwise as } t \rightarrow \infty . $$

Since

$$ \int _{0}^{\infty }f_{0}(\sigma , \Omega _{0}) d\sigma < \infty $$

it follows that, if $z_{0} < r<0$, then, for every $\omega \in \mathscr {B}( \Omega _{0})$,

$$\begin{aligned} \int _{0}^{t} b(\sigma , \omega ) d \sigma &= \int _{0}^{t} B(\sigma ) M(t- \sigma , \omega ) d\sigma + \int _{0}^{t} f_{0}(\sigma , \omega ) d\sigma \\ & \sim e^{r t} K_{r} \hat{M}(r, \omega ) + B_{\infty }\hat{M}(0,\omega ) + \hat{f_{0}}(0, \omega ) \end{aligned}$$

as $t$ tends to infinity.

**Case 3:** $r=0$.

Since $B(t) \sim t D $ as $t \rightarrow \infty $, for every $\varepsilon >0$, there exists a $T_{\varepsilon }>0$ such that for every $t> T_{\varepsilon }$

$$ \left | B(t) - D t \right | < \varepsilon D t. $$

Therefore, for any $\omega \in \mathscr {B}(\Omega _{0})$

$$\begin{aligned} &\frac{\int _{0}^{t} \left | B(\tau )- \tau D \right | M(t-\tau , \omega ) d\tau }{ D \int _{0}^{t} \tau M(t-\tau , \omega ) d\tau } \\ &\leq \frac{\int _{0}^{T_{\varepsilon }} \left | B(\tau )- \tau D \right | M(t-\tau , \omega ) d\tau }{ D \int _{0}^{t} \tau M(t-\tau , \omega ) d\tau } +\varepsilon \frac{\int _{T_{\varepsilon }}^{t} \tau D M(t-\tau , \omega ) d\tau }{ D \int _{0}^{t} \tau M(t-\tau , \omega ) d\tau } \\ & \leq \max _{[0, T_{\varepsilon }]}\left | B(\tau ) - \tau D \right | e^{ z_{0} t} e^{- z_{0} T_{\varepsilon }} \frac{\int _{0}^{\infty }e^{- z_{0} \sigma } M(\sigma , \omega ) d\sigma }{D \int _{0}^{t} \tau M(t-\tau , \omega ) d\tau } + \varepsilon \end{aligned}$$

Since for every $\omega \in \mathscr {B}(\Omega _{0})$, we have that $\int _{0}^{\infty }\tau M(t- \tau , \omega ) d\tau \rightarrow \infty \text{ as } t \rightarrow \infty $ and since $M $ is a $z_{0}$-Laplace component, we can find $\tilde{T}_{\varepsilon }$ such that for every $t > \tilde{T}_{\varepsilon }$ we have

$$ \frac{\int _{0}^{t} \left | B(\tau )- \tau D \right | M(t-\tau , \omega ) d\tau }{ D \int _{0}^{t} \tau M(t-\tau , \omega ) d\tau } < 2 \varepsilon . $$

This is equivalent to

5.10 $$ \int _{0}^{t} B(\tau ) M(t-\tau , \cdot ) d\tau \sim \int _{0}^{t} D \tau M(t-\tau , \cdot ) d\tau \ \text{ setwise as } \ t \rightarrow \infty . $$

Notice that,

$$ \int _{0}^{t} D \tau M(t-\tau , \cdot ) d\tau = D t \int _{0}^{t} M( \tau , \cdot ) d\tau - D \int _{0}^{t} \tau M(\tau , \cdot ) d\tau $$

Since

$$ D t \int _{0}^{t} M(\tau , \cdot ) d\tau \rightarrow \infty \quad \text{ and } \quad \int _{0}^{\infty }\tau M(\tau , \cdot ) d\tau < \infty $$

the conclusion follows. □

#### Proof of Corollary 3.15

**Case 1:** $r>0$.

Thanks to (3.6), we have

$$\begin{aligned} & \left \| e^{- rt} \int _{0}^{t} b(\sigma , \cdot ) d\sigma - \int _{0}^{\infty }C_{r} e^{ - r \sigma } M(\sigma , \cdot ) d\sigma \right \| \\ =& \left \| e^{- rt} \int _{0}^{t} b(\sigma , \cdot ) d\sigma - \int _{0}^{t} C_{r} e^{ - r \sigma } M(\sigma , \cdot ) d\sigma - \int _{t}^{\infty }C_{r} e^{ - r \sigma } M(\sigma , \cdot ) d \sigma \right \| \\ \leq &\left \| e^{- rt} \int _{0}^{t} b(\sigma , \cdot ) d\sigma - \int _{0}^{t} C_{r} e^{ - r \sigma } M(\sigma , \cdot ) d\sigma \right \| + \left \| \int _{t}^{\infty }C_{r} e^{ - r \sigma } M(\sigma , \cdot ) d \sigma \right \| \\ \leq & e^{- rt} \left \| \int _{0}^{t} B(\sigma ) M(t- \sigma , \cdot ) d\sigma - \int _{0}^{t} C_{r} e^{ r \tau } M(t-\tau , \cdot ) d \tau \right \| \\ &+ e^{- rt} \int _{0}^{t}\left \| f_{0}(\sigma , \cdot )\right \| d \sigma + \int _{t}^{\infty }C_{r} e^{ - r \sigma }\left \| M(\sigma , \cdot )\right \| d \sigma \\ \leq & e^{- rt} \int _{0}^{t} \left | B(\sigma ) - C_{r} e^{ r \sigma } \right | \left \| M(t- \sigma , \cdot )\right \| d\sigma + e^{- rt} \int _{0}^{t}\left \| f_{0}(\sigma , \cdot )\right \| d \sigma \\ & + \int _{t}^{\infty }C_{r} e^{ - r \sigma }\left \| M(\sigma , \cdot ) \right \| d \sigma \\ \leq & e^{- rt} \int _{0}^{t} \left | B(\sigma ) - C_{r} e^{ r \sigma } \right | M(t- \sigma , \Omega _{0} ) d\sigma + e^{- rt} \int _{0}^{t} f_{0}( \sigma , \Omega _{0}) d \sigma \\ & + \int _{t}^{\infty }C_{r} e^{ - r \sigma } M(\sigma , \Omega _{0} ) d \sigma . \end{aligned}$$

We now show that both the terms in the right hand side of the last inequality tend to zero as time tends to infinity. Notice that

$$ \int _{t}^{\infty }C_{r} e^{ - r \sigma } M(\sigma , \Omega _{0} ) d \sigma \rightarrow 0 \text{ as } t \rightarrow \infty $$

because $\int _{0}^{\infty }M(\sigma , \Omega _{0}) d\sigma < \infty $. We also know that

$$ e^{- rt} \int _{0}^{t} f_{0}(\sigma , \Omega _{0}) d \sigma \rightarrow 0 \text{ as } t \rightarrow \infty $$

because $f_{0}(\cdot , \Omega _{0})$ is an integrable function. Lastly, we recall that we proved (proof of Theorem 3.14) that

$$ \int _{0}^{t} B(\sigma ) M(t-\sigma , \omega ) d\sigma \sim \int _{0}^{t} C_{r} e^{ r \sigma } M(t-\sigma , \omega ) d\sigma \quad \text{ as } t \rightarrow \infty $$

and therefore

$$ \frac{\int _{0}^{t}\left | B(\sigma )- C_{r} e^{ r \sigma } \right | M(t-\sigma , \omega ) d\sigma }{e^{r t} \int _{0}^{t} C_{r} e^{ - r v} M(v, \omega ) d v } \rightarrow 0 \quad \text{ as } t \rightarrow \infty $$

which implies, since $0< \int _{0}^{\infty }M(\tau , \Omega _{0}) < \infty $, that

$$\begin{aligned} & e^{- rt} \int _{0}^{t} \left | B(\sigma ) - C_{r} e^{ r \sigma } \right | M(t- \sigma , \Omega _{0} ) d\sigma \rightarrow 0 \quad \text{ as } t \rightarrow \infty . \end{aligned}$$

The conclusion follows.

**Case 2:** $r< z_{0} <0 $.

As in Case 1, we have

$$\begin{aligned} & \left \| e^{- rt} \int _{0}^{t} b(\sigma , \cdot ) d\sigma - \int _{0}^{\infty }\left (K_{r} e^{ - r \sigma } + e^{-rt } B_{\infty }\right ) L( \sigma , \cdot ) d\sigma - e^{-rt} \int _{0}^{\infty }f_{0}(\sigma , \cdot ) d\sigma \right \| \\ \leq & \left \| e^{- rt} \int _{0}^{t} B(\sigma ) M(t-\sigma , \cdot ) d\sigma - \int _{0}^{t} \left (K_{r} e^{-r\sigma } + e^{-rt } B_{\infty }\right ) M(\sigma , \cdot ) d\sigma \right \| \\ & + \left \| \int _{t}^{\infty }\left (K_{r} e^{-r\sigma } + e^{-rt } B_{\infty }\right ) M(\sigma , \cdot ) d\sigma \right \| + \left \| e^{-rt} \int _{t}^{\infty }f_{0}(\sigma , \cdot ) d\sigma \right \| \\ \leq & \left \| e^{- rt} \int _{0}^{t} \left ( B(\sigma ) - K_{r} e^{ r \sigma } - B_{\infty }\right ) M(t- \sigma , \cdot ) d\sigma \right \| \\ &+ \left \| \int _{t}^{\infty }\left (K_{r} e^{ - r \sigma } +e^{ -rt} B_{\infty }\right ) M(\sigma , \cdot ) d \sigma \right \| + \left \| e^{-r t} \int _{t}^{\infty }f_{0}(\sigma , \cdot ) d\sigma \right \| \\ \leq & e^{- rt} \int _{0}^{t} \left | B(\sigma ) -K_{r} e^{ r \sigma } - B_{\infty }\right | M(t- \sigma , \Omega _{0}) d\sigma + e^{-r t} \int _{t}^{\infty }f_{0}(\sigma , \Omega _{0}) d\sigma \\ & + \int _{t}^{\infty }\left | K_{r} e^{ - r \sigma } +B_{\infty }\right | M( \sigma , \Omega _{0}) d \sigma . \end{aligned}$$

The three terms on the right hand side of the last inequality tend to zero as time goes to infinity. Indeed,

$$\begin{aligned} & \int _{t}^{\infty }\left ( K_{r} e^{ - r \sigma } + e^{-rt } B_{\infty }\right ) M(\sigma , \Omega _{0}) d \sigma \rightarrow 0 \text{ as } t \rightarrow \infty , \end{aligned}$$

and

$$ e^{-r t} \int _{t}^{\infty }f_{0}(\sigma , \Omega _{0}) d\sigma \leq \int _{t}^{\infty }e^{-r \sigma }f_{0}(\sigma , \Omega _{0}) d\sigma \rightarrow 0 \quad t \rightarrow \infty . $$

Using the fact that $M$ is a $z_{0}$-Laplace component and (5.9) we obtain that

$$ e^{- rt} \int _{0}^{t} \left | B(\sigma ) -K_{r} e^{ r \sigma } - B_{\infty }\right | M(t- \sigma , \Omega _{0}) d\sigma \rightarrow 0 $$

as $t \rightarrow \infty $.

**Case 3:** $r=0$.

We have

$$\begin{aligned} \left \| \frac{1}{t} \int _{0}^{t} b(\sigma , \cdot ) - D \int _{0}^{\infty }M(\sigma , \cdot ) d\sigma \right \| \leq &\frac{1}{t} \int _{0}^{t} \left | B(\sigma ) - t D \right | M(t-\sigma , \Omega _{0}) \\ & + \frac{1}{t} \int _{0}^{t} f_{0}(\sigma , \Omega _{0}) - D \int _{t}^{\infty }M(\sigma , \Omega _{0}). \end{aligned}$$

Notice that

$$ \frac{1}{t} \int _{0}^{t} f_{0}(\sigma , \Omega _{0}) \rightarrow 0 , \quad \int _{t}^{\infty }M(\sigma , \Omega _{0}) \rightarrow 0 \text{ as } t \rightarrow \infty . $$

On the other hand, by (5.10) we deduce that

$$\begin{aligned} & \frac{1}{t} \int _{0}^{t} \left | B(\sigma ) - t D \right | M(t- \sigma , \Omega _{0}) \rightarrow 0 \text{ as } t \rightarrow \infty \end{aligned}$$

 □

### Behaviour of the Population Birth Rate

#### Lemma 5.5

*If Assumption* 3.16*holds*, *then the function* $t \mapsto B(t) $ *is absolutely continuous and therefore there exists* $\tilde{b} \in L^{1}(\mathbb{R}_{+}) $ *such that*

5.11 $$ B(t)= \int _{0}^{t} \tilde{b}(s) ds. $$

Notice that $\tilde{b}(t)$ corresponds to the expected number of individuals that pass the renewal point per unit of time at time $t$ and are offspring of individuals born after time zero.

#### Proof

By Proposition 3.7$B(t)$ is the solution of the reduced renewal equation (3.5) and hence it is given by the formula

5.12 $$ B(t)= \int _{0}^{t} \mu _{R}(ds) z(t-s) + z(t), $$

where $R$ is the resolvent of $K$ (see Appendix A (A.7). Since $K$ is absolutely continuous by Assumption 3.16, the same is true of its resolvent $R$. The assertion now follows from (5.12) because both the convolution and sum of two absolutely continuous functions are absolutely continuous. See [31] for details. □

#### Lemma 5.6

*If Assumption* 3.16*holds*, *then the function* $\tilde{b} $ *satisfies the following equation*

5.13 $$ \tilde{b}(t) = \int _{0}^{t} \tilde{b}(t-s) K' (s) ds+ z'(t) \quad a.e. $$

*with*

$$ \int _{0}^{t} z'(s) ds =z(t). $$

#### Proof

We obtain this result by differentiating (3.5). □

The function $E$ from $\mathbb{R}_{+} \times \mathscr {B} (\Omega _{0})$ to $\mathbb{R}_{+} $ defined by

5.14 $$ E(t, \omega ):=\int _{0}^{t} M(t-s, \omega ) \tilde{b} (s) ds, $$

represents the contribution to the population birth rate by individuals that were born after time 0, and therefore, have crossed the renewal point after time 0. Similarly, the function $\mathscr {Z}$ from $\mathbb{R}_{+} \times \mathscr {B} (\Omega _{0})$ to $\mathbb{R}_{+} $ defined by

5.15 $$ \mathscr {Z}(t, \omega ):=\int _{0}^{t} M(t-s, \omega ) z'(s) ds, $$

is the contribution to the population birth rate by individuals that were born before time 0. The intuitive idea behind renewal equations that those born after time 0 are either direct offspring or descendants (grandchildren, great-grandchildren, etc.) of someone born before time 0 suggests that $E$ should satisfy a renewal equation with forcing function $\mathscr {Z}$. The following lemma shows that this is indeed the case.

#### Lemma 5.7

*Let Assumption* 3.16*hold*. *The function* $E$ *given by* (5.14) *is the unique solution of the renewal equation*

5.16 $$ E(t, \omega ) = \int _{0}^{t} E(t-s, \omega ) \mu _{K}(ds) + \mathscr {Z}(t, \omega ), \quad \omega \in \mathscr {B}(\Omega _{0}), $$

*with* $\mathscr {Z}$ *defined by* (5.15).

#### Proof

The uniqueness stems from the fact that, once $\omega \in \mathscr {B}(\Omega _{0})$ is fixed, the equation is one dimensional. The proof of the uniqueness of the solution of this equation can be found in [27] and[31] (see also Appendix A.2).

To show existence, we integrate both sides of equation (5.13) from 0 to $t$ against $M(t-s, \omega )$ and use (5.16) to obtain

$$\begin{aligned} E(t, \omega )= \int _{0}^{t} M(t-s, \omega ) \left (\int _{0}^{s} \tilde{b}(s-v) \mu _{K} (dv) + z'(s) \right ) ds. \end{aligned}$$

Notice that

$$\begin{aligned} & \int _{0}^{t} M(t-s, \omega ) \int _{0}^{s} \tilde{b}(s-v) \mu _{K} (dv) ds= \int _{0}^{t} \int _{v}^{t} M(t-s, \omega ) \tilde{b}(s-v) ds \mu _{K} (dv) \\ &= \int _{0}^{t} \int _{0}^{t-v} M(t-\sigma -v, \omega ) \tilde{b}( \sigma ) d\sigma \mu _{K} (dv) = \int _{0}^{t} E(t-v, \omega ) \mu _{K} (dv). \end{aligned}$$

This concludes the proof. □

Equation (5.16) formalises the fact that, when we deal with a factorisable kernel $k$, the distribution of the state-at-birth of the offspring of an individual depends only on how long ago the individual passed the reference point.

The following lemma is the mathematical formalisation of the interpretation of $E$ as the contribution to the population birth rate by individuals that were born after time 0.

#### Lemma 5.8

*If Assumption* 3.16*holds*, *then the function* $E(t, \omega )+f_{0}(t, \omega ) $ *is the solution of equation* (3.1).

#### Proof

First of all by the identity (5.2) we have that

$$\begin{aligned} &\int _{0}^{t} E(t-s, \omega ) \mu _{K}(ds) \\ &= \int _{0}^{\infty }\int _{\Omega _{0}} M(r, d\xi ) \int _{0}^{\infty } \chi _{[0,t]}(r+ \sigma ) E(t-r-\sigma , \omega )\mu _{L}(d\sigma , \xi ) dr \\ & = \int _{0}^{t} \int _{\Omega _{0}} M(r, d\xi ) \int _{0}^{t-r} E(t-r- \sigma , \omega ) \mu _{L}(d\sigma , \xi ) dr \end{aligned}$$

From this last formula and from (5.14) we obtain that

$$\begin{aligned} &\int _{0}^{t} E(t-s, \omega ) \mu _{K}(ds) \\ &= \int _{0}^{t} \int _{\Omega _{0}} M(r, d\xi ) \int _{0}^{t-r} \int _{0}^{t-r- \sigma } \tilde{b} (t-r-\sigma -s) M(s, \omega ) ds \mu _{L}(d\sigma , \xi ) dr \\ &= \int _{0}^{t} \int _{\Omega _{0}} M(r, d\xi ) \int _{0}^{t-r} \int _{\sigma }^{t-r} \tilde{b} (t-r-s) M(s-\sigma , \omega ) ds \mu _{L}(d \sigma , \xi ) dr \\ &= \int _{0}^{t} \int _{\Omega _{0}} M(r, d\xi ) \int _{0}^{t-r} \tilde{b} (t-r-s) \int _{0}^{s} M(s-\sigma , \omega ) \mu _{L}(d \sigma , \xi ) ds dr \\ &= \int _{0}^{t} \int _{\Omega _{0}} M(r, d\xi ) \int _{0}^{t-r} \tilde{b} (t-r-s) k(s, \xi , \omega ) ds dr \\ &= \int _{0}^{t} \int _{0}^{t-s} \int _{\Omega _{0}} M(r, d\xi ) \tilde{b} (t-r-s)dr k(s, \xi , \omega )ds. \end{aligned}$$

We notice that a formula analogous to (5.2) holds for $z'$, viz.

$$ \int _{0}^{\infty }f(s) z'(s) ds = \int _{0}^{\infty }\int _{\Omega _{0}} \int _{0}^{\infty }f(s+ \eta ) f_{0}(\eta , d\xi ) \mu _{L}(ds, \xi ) d \eta . $$

This allows us to show that

$$\begin{aligned} \mathscr {Z}(t,\omega )& =\int _{0}^{t} M(t-s, \omega ) z'(s) ds = \int _{0}^{\infty }\int _{\Omega _{0}} \int _{0}^{\infty }M(t-s- \eta , \omega )\chi _{[0,t]}(s+\eta ) \mu _{L}(ds, \xi ) d\eta \\ &= \int _{0}^{t} \int _{\Omega _{0}} \int _{0}^{ t -\eta } M(t-s- \eta , \omega ) \mu _{L}(ds, \xi ) f_{0}(\eta , d\xi )d\eta \\ &= \int _{0}^{t} \int _{\Omega _{0}} f_{0}(\eta , d\xi ) k(t-\eta , \xi , \omega )d\eta \end{aligned}$$

Therefore,

$$\begin{aligned} E(t, \omega ) + f_{0}(t, \omega ) =& \int _{0}^{t} E(t-s, \omega ) \mu _{K}(ds) + \mathscr {Z}(t,\omega ) + f_{0}(t, \omega ) \\ =& \int _{0}^{t} \int _{0}^{t-s} \int _{\Omega _{0}} M(r, d\xi ) \tilde{b} (t-r-s, \omega )dr k(s, \xi , \omega )ds \\ &+ \int _{0}^{t} \int _{\Omega _{0}} f_{0}(\eta , d\xi ) k(t-\eta , \xi , \omega )d\eta + f_{0}(t, \omega ) \\ =& \int _{0}^{t} \left ( E(t-s, d\xi ) + f_{0}(t-s, d\xi ) \right )k(s, \xi , \omega )ds +f_{0}(t, \omega ) \end{aligned}$$

and therefore $E(t, \omega ) + f_{0}(t, \omega )=b(t, \omega )$. □

#### Proof of Theorem 3.17

First of all we notice that for fixed $\omega \in \mathscr {B}(\Omega _{0})$, equation (5.16) is a one dimensional renewal equation. Therefore the asymptotic behaviour of its solutions can be obtained by the Renewal Theorem.

**Case 1:** $r=0$.

For any $\omega \in \mathscr {B}(\Omega _{0})$ we have

$$ \mathscr {Z}(t, \omega ) \leq \int _{0}^{t} z'(s) \sup _{\sigma \in \mathbb{R}_{+}} M(\sigma , \Omega _{0}) \leq z(t) \sup _{\sigma \in \mathbb{R}_{+}} M(\sigma , \Omega _{0}). $$

Therefore, for any $\omega \in \mathscr {B}(\Omega _{0})$, the map $t \mapsto \mathscr {Z} (t, \omega )$ is bounded by the directly Riemann integrable function $z$, and therefore directly integrable.

We can now apply the alternative version of the Renewal Theorem to conclude that, for every $\omega \in \mathscr {B}(\Omega _{0})$

$$ E(t, \omega ) \sim \frac{\int _{0}^{\infty }\int _{0}^{\tau }M(\tau -s, \omega ) z'(ds) d\tau }{\int _{0}^{\infty }s \mu _{K}(ds)} \text{ as } t \rightarrow \infty . $$

Since the Laplace transform of a convolution is the product of the Laplace transforms we have

$$\begin{aligned} \int _{0}^{\infty }\int _{0}^{\tau }M(\tau -s, \omega ) z'(s) ds d\tau = \hat{z'}(0)\hat{M}(0, \omega )= z_{\infty }\hat{M}(0, \omega ), \end{aligned}$$

and the result follows, since $f_{0}(\cdot , \Omega _{0}) $ is bounded.

**Case 2:** $r>0$.

Fix $\omega \in \mathscr {B}(\Omega _{0})$. Since $\mathscr {Z}$ is directly Riemann integrable and $e^{-r t} $ is a monotone decreasing integrable function we conclude that $\mathscr {Z}_{\#}(t):= e^{-r t} \mathscr {Z}(t) $ is directly Riemann integrable. The alternative version of the Renewal Theorem applied to the equation

$$ E_{\#}(t, \omega ) = \int _{0}^{t} E_{\#} (t-s, \omega ) \mu _{K_{\#}}(ds) + \mathscr {Z}_{\#}(t, \omega ) $$

with $E_{\#}(t, \omega ):= e^{-r t} E(t, \omega )$, and $\mu _{K_{\#}}(t):= e^{-r t} \mu _{K}(t)$, yields

$$ E(t, \omega ) \sim e^{ rt} \frac{\int _{0}^{\infty }\mathscr {Z}(\tau , \omega ) e^{-r \tau } d\tau }{ \int _{0}^{\infty }\tau e^{-r \tau } \mu _{K}(d\tau )}. $$

Taking the Laplace transform of (5.15) we obtain

$$\begin{aligned} \hat{\mathscr {Z}}(r, \omega ) = \hat{M}(r, \omega ) \hat{z'}(r) = r \hat{M}(r, \omega ) \hat{z}(r). \end{aligned}$$

It follows that

$$ \frac{\int _{0}^{\infty }\mathscr {Z}(\tau , \omega ) e^{-r \tau } d\tau }{\tau e^{-r \tau } \mu _{K}(d\tau )}= r C_{r} \int _{0}^{\infty }e^{- r \tau } M(\tau , \omega ) d\tau , $$

and this concludes the proof for this case.

**Case 3:** $r<0$.

Consider the weighted equation

$$ E_{\#}(t, \omega ) = \int _{0}^{t} E_{\#} (t-s, \omega ) \mu _{K_{\#}}(ds) + \mathscr {Z}_{\#}(t, \omega ). $$

Notice that, for any $\omega \in \mathscr {B}(\Omega _{0}) $,

$$ \mathscr {Z}_{\#}(t, \omega ) =e^{-rt} \mathscr {Z}(t, \omega ) \leq e^{- z_{0} t } \mathscr {Z}(t, \omega ). $$

We already know that $\mathscr {Z} $ is directly Riemann integrable and that the map $t \mapsto e^{- z_{0} t } \mathscr {Z}(t, \omega )$ is integrable. As a consequence we have that $\mathscr {Z}_{\#}$ is directly Riemann integrable. Applying the alternative version of the Renewal Theorem we obtain

$$ E(t, \omega ) \sim e^{ rt} \frac{\int _{0}^{\infty }\mathscr {Z}(\tau , \omega ) e^{-r \tau } d\tau }{ \int _{0}^{\infty }\tau e^{-r \tau } \mu _{K}(d\tau )}. $$

Moreover,

$$\begin{aligned} r K_{r} = - z_{\infty }+ \int _{0}^{\infty }r e^{-rs} (z_{\infty }- z(s)) ds = \int _{0}^{\infty }e^{-rs} z'(s) ds \end{aligned}$$

where the last equality follows from integration by parts and the facts $z(0)=0$ and $\lim _{t \rightarrow \infty } e^{- r t }\left ( z_{\infty }- z(t) \right ) =0$. □

#### Proof of Corollary 3.18

Let $r>0$ and notice that

$$\begin{aligned} \left \| e^{-rt } b(t, \cdot ) - r C_{r} \hat{M}(r, \cdot ) \right \| & \leq \left \| e^{-rt }\int _{0}^{t} \tilde{b}(t- \sigma ) M(\sigma , \cdot ) d\sigma - r C_{r} \int _{0}^{t} e^{-r\sigma } M(\sigma , \cdot ) d\sigma \right \| \\ & + e^{-rt} f_{0}(t, \Omega _{0}) + r C_{r} \int _{t}^{\infty }e^{-r \sigma } M(\sigma , \Omega _{0}) d\sigma \end{aligned}$$

Notice that since $f_{0}(\cdot , \Omega _{0}) $ is bounded, then

$$ e^{-rt} f_{0}(t, \Omega _{0}) \rightarrow 0 \text{ as } t \rightarrow \infty . $$

On the other hand it is also true that, since $M$ is a measure Laplace component, then

$$ \int _{t}^{\infty }e^{-r\sigma } M(\sigma , \Omega _{0}) d\sigma \rightarrow 0 \text{ as } t \rightarrow \infty . $$

By Theorem (3.17) we also have that

$$ \left \| e^{-rt }\int _{0}^{t} \tilde{b}(t- \sigma ) M(\sigma , \cdot ) d\sigma - r C_{r} \int _{0}^{t} e^{-r\sigma } M(\sigma , \cdot ) d \sigma \right \| \rightarrow 0 \text{ as } t \rightarrow 0. $$

With the same argument, the other two statements of the Corollary follow. □

We state a sufficient assumption on the components that guarantees the absolute continuity of $B$.

#### Assumption 5.9

We assume that $L$ is of the form

5.17 $$ L(\sigma , \xi ):= f(\xi ) H(\sigma - h(\xi )) $$

with $f: \Omega _{0} \rightarrow \mathbb{R}_{+}$ Lipschitz continuous and bounded by 1, $h: \Omega _{0} \rightarrow \mathbb{R}_{+} $ measurable and such that

$$ \sup _{\xi \in \Omega _{0}} e^{- z_{0} h(\xi )} f(h(\xi )) < \infty . $$

Moreover, we assume that the function

$$ t \mapsto \int _{0}^{t} \int _{ \Omega _{0}} M(\sigma , d\xi ) H(t- \sigma - \tau (\xi )) d \sigma $$

is Lipschitz continuous with Lipschitz constant $c$.

#### Lemma 5.10

*Let* $M $ *and* $L$ *be* $z_{0}$-*Laplace components satisfying Assumption* 5.9, *then the functions* $t \mapsto K(t) $ *and* $t \mapsto z(t) $ *are Lipschitz continuous functions*.

#### Proof

Notice that,

$$\begin{aligned} & K(t) = \int _{0}^{t} \int _{ \Omega _{0}} M(\sigma , d\xi ) f(\xi ) H({t}- \sigma - h(\xi )) d \sigma \end{aligned}$$

is Lipschitz continuous, since $f$ is bounded and

$$ \int _{0}^{t} \int _{ \Omega _{0}} M(\sigma , d\xi ) H({t}- \sigma - h( \xi )) d \sigma $$

is Lipschitz continuous by hypothesis.

Next note that $z$ can be rewritten, plugging in equation (3.4) the formula for the forcing function (3.2), as

$$ z(t)= \int _{-\infty }^{0} \int _{ \Omega _{0}} \int _{0}^{t} \left ( \int _{ \Omega _{0}} k(\sigma -s,x,d\xi ) H(t-\sigma -\tau (\xi )) \right ) f(\xi ) d\sigma \Phi (ds, dx). $$

With a similar argument to the one used to prove that $K$ is Lipschitz continuous we can see that when the map

$$ t \mapsto \int _{0}^{t} \int _{ \Omega _{0}} k(\sigma -s, x, d\xi ) H(t- \sigma -\tau (\xi )) d\sigma $$

is Lipschitz, so is $z$.

Using the fact that $k$ is a factorisable kernel with kernel components $L$ and $M $ we obtain that

$$\begin{aligned} &\int _{0}^{t} \int _{ \Omega _{0}} k(\sigma -s, x, d\xi ) H(t- \sigma -h(\xi )) d\sigma \\ &= \int _{0}^{t} \int _{ \Omega _{0}} \int _{0}^{\sigma -s} \mu _{L}(dv, x) M(\sigma -s-v, d\xi ) H(t-\sigma -h(\xi )) d\sigma \\ &= \int _{0}^{t} \int _{ \Omega _{0}} f(x) M(\sigma -s-h(x), d\xi ) H(t- \sigma -h(\xi )) d\sigma . \end{aligned}$$

From Assumption 5.9 and from the fact that $f$ is bounded we conclude that z is Lipschitz. □

Motivated by the models we present some examples of kernel components that satisfy Assumption 5.9.

#### Lemma 5.11

*Assume* $L$ *to have the form* (5.17).

*If the measure* $M(t, \cdot ) $ *is absolutely continuous with respect to the Lebesgue measure and has density* $d(t, \cdot )$, *which is a bounded map from* $\mathbb{R}_{+} \times \mathscr {B}( \Omega _{0})$ *to* $\mathbb{R}_{+}$, *then Assumption* 5.9*is satisfied*.

*If*, *instead*, $M(\sigma , \omega )=l(\sigma ) \delta _{p(\sigma )}(\omega )$, *where* $l: \mathbb{R}_{+} \rightarrow \mathbb{R}_{+}$ *is a bounded function and* $p : \mathbb{R}_{+} \rightarrow \Omega _{0}$ *a measurable function*, *then Assumption* 5.9*is satisfied provided that the map* $\sigma \mapsto G(\sigma ):= \sigma + h(p(\sigma ))$ *is invertible and the inverse is Lipschitz continuous*.

In the lemma $l $ represents the probability per unit of time that an individual, that passed by the renewal point $\sigma $ time ago, reaches age $\sigma $ and reproduces. The fact that $M(\sigma , \omega )=l(\sigma ) \delta _{p(\sigma )}(\omega )$ means that this individual will give birth to an offspring with state at birth $p(\sigma )$.

#### Proof

If $M(\sigma , \cdot ) $ is absolutely continuous with respect to the Lebesgue measure and has density $d(\sigma , \cdot )$, we have that

$$\begin{aligned} \int _{0}^{t} \int _{ \Omega _{0}} M(\sigma , d\xi ) H(t- \sigma - h( \xi )) d \sigma &= \int _{ \Omega _{0}}\int _{0}^{t} d(\sigma ,\xi ) H(t- \sigma - h(\xi )) d \sigma d\xi \\ & = \int _{ \Omega _{0}} \int _{0}^{t - h(\xi )} d(\sigma ,\xi ) d \sigma d\xi . \end{aligned}$$

By the hypothesis on $d$, we conclude that the map

$$ t \mapsto \int _{ \Omega _{0}} \int _{0}^{t - h(\xi )} d(\sigma ,\xi ) d \sigma d\xi $$

is Lipschitz continuous and Assumption 5.9 holds.

If $M(\sigma , \omega )= l(\sigma )\delta _{p(\sigma )}(\omega )$, then

$$\begin{aligned} &\int _{0}^{t} \int _{ \Omega _{0}} M(\sigma , d\xi ) H(t- \sigma - h( \xi )) d \sigma = \int _{0}^{t} \int _{ \Omega _{0}} l(\sigma ) \delta _{p(\sigma )}(d\xi ) H(t- \sigma - h(\xi )) d \sigma \\ &= \int _{0}^{t} l(\sigma ) H(t- \sigma - h(p(\sigma ) )) d \sigma = \int _{0}^{G^{-1}(t)} l(\sigma ) d\sigma \end{aligned}$$

and therefore, since $G^{-1}$ is Lipschitz continuous and $l$ is bounded, Assumption 5.9 holds. □

### Proofs of the Results of Sect. 4

#### Proof of Lemma 4.3

First of all, given the assumptions on $g$, $\gamma $, $\eta $, and $\mu $ we have that $k$ is a kernel.

Moreover, for $\xi $ less than $\overline{x}$ and $a$ such that $X(a,\xi ) > \overline{x}$

$$\begin{aligned} \mathscr {F}(a, \xi )& = \exp \left (- \int _{0}^{a} \left (\gamma (X(s, \xi )) +\mu (X(s,\xi )) \right )ds \right ) \\ & =\exp \left (- \int _{\xi }^{X(a,\xi )} \frac{\gamma (z) +\mu (z) }{g(z)} dz \right ) \\ &=\exp \left (- \int _{\xi }^{\overline{x}} \frac{\gamma (z) +\mu (z)}{g(z)} dz \right ) \exp \left (- \int _{ \overline{x}}^{X(a,\xi )} \frac{\gamma (z)+\mu (z)}{g(z)} dz \right ) \\ & =\hat{\mathscr {F}}(\xi , \overline{x} ) \mathscr {F}(a-\tau (\xi , \overline{x}), \overline{x} ). \end{aligned}$$

Therefore

$$\begin{aligned} k(a, \xi , \omega )& = \mathscr {F}(a,\xi )\gamma (X(a,\xi ))\eta (X(a, \xi ),\omega ) \\ & = \mathscr {F}(a-\tau (\xi ,\overline{x}), \overline{x})\gamma (X(a- \tau (\xi ,\overline{x}),\overline{x}))\eta (X(a-\tau (\xi , \overline{x}),\overline{x}),\omega ) \mathscr {F}(\xi ,\overline{x}) \\ &= \int _{0}^{a} \mathscr {F}(a-\sigma , \overline{x})\gamma (X(a- \sigma ,\overline{x}))\eta (X(a-\sigma ,\overline{x}),\omega ) \delta _{ \tau (\xi ,\overline{x})}(d\sigma )\mathscr {F}(\xi ,\overline{x}) \\ & = \int _{0}^{a} \mu _{L}(d\sigma ,\xi ) M(a-\sigma , \omega ). \end{aligned}$$

Notice that, for every $\xi \in \Omega _{0}$ we have that $L(\cdot , \xi ) \in NBV(\mathbb{R}_{+}) $, moreover

$$ \sup _{ (t,\xi ) \in \mathbb{R}_{+} \times \Omega _{0}} L(t,\xi )= \sup _{ (t, \xi ) \in \mathbb{R}_{+} \times \Omega _{0}} \hat{\mathscr {F}}(\xi ,\overline{x}) H(t-\tau (\xi ,\overline{x})) \leq 1 $$

and

$$\begin{aligned} \sup _{\xi \in \Omega _{0} } \int _{0}^{\infty }e^{-z_{0} \sigma } \mu _{L}(d \sigma , \xi ) &= \sup _{\xi \in \Omega _{0}} e^{-z_{0} \tau (\xi , \overline{x})} \mathscr {F}(\xi , \overline{x}) \\ &=\sup _{\xi \in \Omega _{0}} e^{-z_{0} \tau (\xi , \overline{x})} e^{ - \int _{0}^{\tau (\xi , \overline{x})} \gamma (X(s, \overline{x} )) + \mu (X(s, \overline{x} )) ds } \\ & \leq \sup _{\xi \in \Omega _{0}} e^{-z_{0} \tau (\xi , \overline{x})} < \infty . \end{aligned}$$

Next consider the component $M$. In this case

$$\begin{aligned} \sup _{\mathbb{R}_{+}} M(t, \Omega _{0}) \leq 2 e^{ - \int _{0}^{t} \gamma (X(s, \overline{x} )) ds } \gamma (X(t, \overline{x})). \end{aligned}$$

By assumption (4.1) we have that

$$ \lim _{t \rightarrow \infty } e^{- \int _{0}^{t} \gamma (X(s, \overline{x})) ds } \gamma (X(t, \overline{x})) =0, $$

implying

$$ \sup _{t \in \mathbb{R}_{+}} M(t, \Omega _{0}) < \infty . $$

On the other hand, we also have that

$$\begin{aligned} \int _{0}^{\infty }e^{- z_{0} t} M(t, \Omega _{0}) dt \leq 2 \int _{0}^{\infty }e^{-z_{0} t} \mathscr {F}(t, \overline{x}) \gamma (X(t, \overline{x})) dt < \infty . \end{aligned}$$

In the case of bounded lifetime this follows directly from the fact that $M(t, \Omega _{0})=0 $ if $t > \tau (\overline{x},1) $. If, instead $g(1)=0$, $g'(1) \neq 0$ and $\gamma (1) >0$, then

$$ e^{-\int _{\overline{x}}^{X(t, \overline{x})} \frac{\gamma (z)}{g(z)} dz} \sim e^{-t \gamma (1) } \text{ as } t \rightarrow \infty . $$

Consequently, since $z_{0} >- \gamma (1)$

$$\begin{aligned} \int _{0}^{\infty }e^{-z_{0} t} \mathscr {F}(t, \overline{x}) \gamma (X(t, \overline{x})) dt &= \lim _{t \rightarrow \infty } e^{-z_{0} t -\int _{ \overline{x}}^{X(t, \overline{x})} \frac{\gamma (z)}{g(z)} dz} + z_{0} \int _{0}^{\infty }e^{-z_{0} t-\int _{\overline{x}}^{X(t, \overline{x})} \frac{\gamma (z)}{g(z)} dz} dt < \infty . \end{aligned}$$

 □

#### Proof of Proposition 4.4

We first consider the case $\eta (x, \omega )=2 \delta _{1/2x}(\omega ) $ and $g(2x) < 2 g(x) $. We aim at applying Lemma 5.11 to deduce that $K $ is Lipschitz continuous, and hence non-arithmetic. To this end we consider the function $G(s):=\tau \left (\frac{1}{2} X(s, \overline{x}), X(s,\overline{x}) \right )$ and notice that it is a monotonically increasing function and hence invertible. Indeed,

$$\begin{aligned} \frac{d}{ds}G(s)= \frac{d}{ds} \int _{\frac{1}{2} X(s, \overline{x})}^{ X(s , \overline{x})} \frac{1}{g(z)} dz = 1- \frac{ g(X(s, \overline{x}))}{2 g\left ( \frac{1}{2} X(s, \overline{x}) \right )} >0. \end{aligned}$$

Moreover,

$$\begin{aligned} & \left | G(s_{1})- G(s_{2}) \right |= \left | s_{1}-s_{2}\right | + \left | \int _{\frac{1}{2} X(s_{2}, \overline{x})}^{ \frac{1}{2} X(s_{1} , \overline{x})} \frac{1}{g(z)} dz \right | \geq \left | s_{1}-s_{2} \right | \\ &+ \frac{1}{2} \frac{1}{\sup _{ z \in \Omega } g(z)} \left | X(s_{2}, \overline{x}) - X(s_{1} , \overline{x}) \right | \geq c \left | s_{1}-s_{2} \right | \end{aligned}$$

for a constant $c>0$. Hence $G^{-1} $ is a Lipschitz function. By Lemma 5.11 we deduce that $K$ and $z$ are Lipschitz continuous (hence $K$ is not arithmetic) and we can apply Theorem 3.17.

If $\eta (x, \cdot ) $ is absolutely continuous with respect to the Lebesgue measure and has bounded density, we can apply the first part of Lemma 5.11 to deduce that $K$ is Lipschitz continuous and hence non-arithmetic and that $z$ is Lipschitz continuous and we can again apply Theorem 3.17. □

#### Proof of Proposition 4.7

Because the kernel (4.8) is just a particular case of kernel (4.4) we can define $\hat{\mathscr {F}}(x,y)$ and $\tau (x,y) $ as in the cell fission model and by a small notational adaptation of the proof of Proposition 4.4 we deduce that the kernel $k$ is a factorisable $- \Lambda $-kernel.

For this model the map $G$ in Lemma 5.11 is given by

$$ G(\eta ) := \textstyle\begin{cases} \eta &\text{ if } \eta < \tau (x_{c},\overline{x}), \\ \tau (f(X(\eta , \overline{x})), \overline{x}) + \eta &\text{ if }\eta \geq \tau (x_{c},\overline{x}). \end{cases} $$

It is a strictly monotone function and therefore it has an inverse, $G^{-1}$. Notice also that

$$\begin{aligned} \tau (f(X(\eta , \overline{x})), \overline{x})&= - \int ^{f(X(\eta , \overline{x} ))}_{ \overline{x}} \frac{1}{g(z)} dz \\ &= \textstyle\begin{cases} 0 &\text{ if } \eta < \tau (x_{c}, \overline{x}) \\ - \int ^{\overline{x} + 2 x_{c} - 2 X(\eta , \overline{x} )}_{ \overline{x} } \frac{1}{g(z)} dz &\text{ otherwise } \end{cases}\displaystyle \end{aligned}$$

Since $0 < \inf _{x \in \Omega }\left | 1/g(x) \right |< \infty $, the inverse $G^{-1} $ is Lipschitz continuous and with the aid of Lemma 5.11 we deduce that the assumptions of Theorem 3.17 hold. The basic reproduction number is

$$\begin{aligned} R_{0}&=\Lambda \int _{0}^{\infty }\int _{\Omega _{0} } \mathscr {F}(a, \overline{x}) \delta _{f(X(a, \overline{x}))}(d\xi ) da = \Lambda \int _{0}^{\infty }e^{-a \Lambda } da =1 \end{aligned}$$

and therefore $r=0$. We conclude that $b(t, \cdot )$ converges to a steady state. □

## A Short Excursion into the Formalism of Perturbed Semigroups of Operators

In this section we relate the results of the previous sections to the formalism of semigroups of operators.

The concrete population model we studied in Sect. 4 can be reformulated as an abstract Cauchy problem of the form

6.1 $$\begin{aligned} \dot{u}(t) =&A_{0} u(t)+B u(t), \quad t>0, \end{aligned}$$

6.2 $$\begin{aligned} u(0) =&u_{0} \end{aligned}$$

for a function $u$ defined on ${\mathbb{R}}_{+}$ and taking on values in a Banach space $X$. In (6.1) the operator $A_{0}: \mathscr {D}(A_{0}) \subset X \rightarrow X $ is the generator of a strongly continuous semigroup $T_{0}$ of bounded linear operators on $X$ and the perturbation $B: X \rightarrow X $ is a bounded linear operator. The initial value $u_{0}$ belongs to $X$. Our line of reasoning extends to (in particular relatively bounded) unbounded operators $B$ [25, 54, 55].

The solution $u(t)$ of (6.1) & (6.2) satisfies the following abstract integral equation (variation-of-constants formula aka Duhamel’s formula)

6.3 $$ u(t)=T_{0}(t) u_{0} + \int _{0}^{t} T_{0}(t-\tau ) Bu(\tau ) d\tau . $$

In our context $T_{0}$ describes development and survival, while $B$ describes reproduction. In [18] it is explained how (6.3) can be obtained from modeling considerations, therefore we focus on (6.3). We refer to [25] for various ways of giving a precise meaning to the integral.

Asynchronous (balanced) exponential growth of operator semigroups has been studied by many authors, for instance in [34, 54, 55, 61].

Here we focus on formula manipulations and we do not state precise assumptions and rigorous results. Indeed, in the earlier sections we have rigorously treated the concrete population models; when we put these into a semigroup framework, lots of technical issues have to be taken care of and there would not be additional results. Yet, from the formula manipulation point of view, the semigroup setting relates our approach based on renewal equations, via an *abstract* renewal equation, to the PDE approach and thus enhances, so we hope, understanding.

The abstract renewal equation

6.4 $$ v(t)=B T_{0}(t) u_{0} + \int _{0}^{t} B T_{0}(t-\tau ) v(\tau ) d \tau $$

is obtained by applying $B$ to (6.3) and replacing $Bu(t)$ by $v(t)$. The standard way of proving existence and uniqueness of a solution is based on the contraction mapping principle.

Once (6.4) is solved, we define $u(t) $ by

$$ u(t)=T_{0}(t) u_{0} + \int _{0}^{t} T_{0}(t-\tau ) v(\tau ) d\tau . $$

It requires a bit of formula manipulation to next show that $T(t) u_{0} := u(t)$ defines a semigroup of operators, see [25] and the references in there.

For special $B$ or special $(B, T_{0})$ combinations, (6.4) reduces to a finite dimensional equation. We refer to [25] for an account of how this works for delay equations. Here we first provide some simple one-dimensional examples before explaining the situation considered in the previous sections.

### Example 6.1

Assume that, for every $\phi \in X$

$$ B \phi = \langle q^{\diamond }, \phi \rangle q \text{ with } q \in X, q^{\diamond }\in X^{*}. $$

Define

$$ k(t)=\langle q^{\diamond }, T_{0}(t) q \rangle $$

and

$$ z_{0}(t)= \langle q^{\diamond }, T_{0}(t) u_{0} \rangle . $$

Let $z$ be the solution of

6.5 $$ z=z_{0} + k * z $$

then, $v(t)=z(t) q $ solves (6.4).

Indeed plugging in equation (6.5) the formulas for $z_{0}$ and $k$, and multiplying the resulting equation by $q$, we obtain equation (6.4).

We denote the dual space of $X$ by $X^{\ast }$ and use duality brackets for the pairing of elements in $X$ and $X^{\ast }$: $\langle x^{\ast }, x\rangle = x^{\ast }(x), \,\, x \in X,\, x^{\ast }\in X^{\ast }$.

### Example 6.2

Assume that for every $\phi \in X$

$$ B T_{0}(t) \phi = \langle q^{\diamond }(t), \phi \rangle q \text{ with } q \in X \text{ and } q^{\diamond }(t) \in X^{*} \text{ for every } t >0. $$

Define

$$ k(t)=\langle q^{\diamond }(t) , q \rangle $$

and

$$ z_{0}(t)= \langle q^{\diamond }(t) , u_{0} \rangle . $$

Let $z$ be the solution of (6.5). Then $v(t)=z(t) q $ solves (6.4).

### Example 6.3

Assume that for every $\phi \in X$

$$ B T_{0}(t) \phi = \int _{0}^{t} \langle q^{\diamond }(\sigma ), \phi \rangle q(t-\sigma ) d\sigma \quad q(t) \in X, q^{\diamond }(t) \in X^{*} \text{ for every } t >0. $$

Define

$$ k(t)=\int _{0}^{t} \langle q^{\diamond }(\sigma ), q (t-\sigma ) \rangle d\sigma $$

and

$$ z_{0}(t)= \langle q^{\diamond }(t) , u_{0} \rangle . $$

Let $z$ be the solution of (6.5). Then $v(t)=\int _{0}^{t} z(\sigma ) q(t-\sigma ) d\sigma $ solves (6.4).

This follows from the fact that, if $v(t)=\int _{0}^{t} z(\sigma ) q(t-\sigma ) d\sigma $, then

6.6 $$ \int _{0}^{t} BT_{0}(t-\tau ) v(\tau ) d\tau = \int _{0}^{t} (k * z ) ( \sigma ) q(t-\sigma ) d\sigma . $$

Indeed,

$$\begin{aligned} &\int _{0}^{t} (k * z)(\sigma ) q(t-\sigma ) d\sigma = \int _{0}^{t} \int _{0}^{\sigma }k(\sigma -s) z(s) ds q(t-\sigma ) d\sigma \\ &= \int _{0}^{t} \int _{0}^{\sigma }\int _{0}^{\sigma - s} \langle q^{\diamond }(r) , q(\sigma -s - r) \rangle z(s) q(t-\sigma ) dr ds d \sigma \\ &= \int _{0}^{t} \int _{0}^{\sigma }\int _{0}^{\sigma - r} \langle q^{\diamond }(r) , q(\sigma -s - r) \rangle z(s) q(t-\sigma ) ds dr d \sigma \\ &= \int _{0}^{t} \int _{0}^{\sigma }\langle q^{\diamond }(r) , \int _{0}^{ \sigma - r} z(s) q(\sigma -s -r) ds \rangle q(t-\sigma ) dr d\sigma \\ &= \int _{0}^{t} \int _{0}^{\sigma }\langle q^{\diamond }(r) , v(\sigma -r) \rangle q(t-\sigma ) dr d\sigma \\ &= \int _{0}^{t} \int _{0}^{t-r} \langle q^{\diamond }(r) , v(\tau ) \rangle q(t-\tau -r) d\tau dr \\ &= \int _{0}^{t} \int _{0}^{t-\tau } \langle q^{\diamond }(r) , v(\tau ) \rangle q(t-\tau -r) dr d\tau \\ &= \int _{0}^{t} BT_{0}(t-\tau ) v(\tau ) d\tau . \end{aligned}$$

As a consequence of (6.6) we have that (6.4) reduces to

$$ 0=\int _{0}^{t} \left ( z(\sigma )- \langle q^{\diamond }(\sigma ) , u_{0} \rangle - (k* z)(\sigma ) \right ) q(t-\sigma ) d\sigma $$

and the statement of the example follows.

In our setting, we assume that there exist functions $q: {\mathbb{R}}_{+} \to X$ and $Q^{\diamond }:{\mathbb{R}}_{+} \to X^{\ast }, \,\, Q^{\diamond }(0)=0$ such that for every $\phi $ belonging to the range of $B$,

6.7 $$ BT_{0} (t) \phi = \int _{0}^{t} d_{\sigma }\langle Q^{\diamond }(\sigma ) , \phi \rangle q(t-\sigma ), \quad t>0. $$

For the integral to make sense, we have to assume that $\sigma \mapsto \langle Q^{\diamond }(\sigma ) , \phi \rangle $ is of bounded variation. The notation $d_{\sigma }\langle Q^{\diamond }(\sigma ) , \phi \rangle $ represents the integration against the measure corresponding to the function $\langle Q^{\diamond }(\sigma ) , \phi \rangle $.

### Remark 6.4

The reason why we assume that formula (6.7) holds only for $\phi $ in the range of $B$, is that this is what we need for the following integral manipulations. And, in fact, (6.7) does not hold for all $\phi \in X$ in the examples presented in Sect. 4.

### Lemma 6.5

*Assume that identity* (6.7) *holds*, *then*

$$ \int _{0}^{t} BT_{0}(\sigma ) \phi d\sigma = \int _{0}^{t} \langle Q^{\diamond }(\sigma ) , \phi \rangle q(t-\sigma ) d\sigma , $$

*for every* $\phi $ *in the range of* $B$.

### Proof

Integrate (6.7). □

### Corollary 6.6

*Assume that the identity* (6.7) *holds*. *Then the integrated version of* (6.4):

6.8 $$\begin{aligned} \int _{0}^{t} v(\tau ) d\tau =& \int _{0}^{t} \langle Q^{\diamond }( \sigma ) , u_{0}\rangle q(t- \sigma ) d\sigma \\ & + \int _{0}^{t} \int _{0}^{t-\tau } \langle Q^{\diamond }(\sigma ) , v( \tau ) \rangle q(t- \sigma - \tau ) d\sigma d\tau \end{aligned}$$

*holds*.

### Proof

By (6.4) we know that

$$ \int _{0}^{t} v(\tau ) d\tau =\int _{0}^{t} B T_{0}(\tau ) u_{0} d \tau + \int _{0}^{t} \int _{0}^{\sigma }B T_{0}(\sigma -\tau ) v(\tau ) d \tau d \sigma . $$

First of all, by (6.7) we have

$$ \int _{0}^{t} B T_{0}(\tau ) u_{0} d\tau = \int _{0}^{t} \langle Q^{\diamond }(\sigma ), u_{0} \rangle q(t- \sigma ) d\sigma . $$

Moreover,

$$\begin{aligned} & \int _{0}^{t} \int _{0}^{\sigma }B T_{0}(\sigma -\tau ) v(\tau )d \tau d\sigma = \int _{0}^{t} \int _{\tau }^{t} B T_{0}(\sigma -\tau ) v( \tau ) d\sigma d \tau \\ & = \int _{0}^{t} \int _{0}^{t-\tau } B T_{0}(r)v(\tau ) dr d \tau = \int _{0}^{t} \int _{0}^{t-\tau } \langle Q^{\diamond }(r) , v(\tau ) \rangle q(t- \tau -r) dr d \tau . \end{aligned}$$

 □

The following corollary represents the one dimensional reduction of the abstract renewal equation (6.4).

### Corollary 6.7

*Assume that* $Z$ *is a bounded variation function and is the solution of*

$$ Z(t)=\int _{0}^{t} \mu _{K}(d\tau ) Z(t- \tau ) + Z_{0}(t) $$

*with* $Z(0)=0$ *and with*

$$ Z_{0}(t):= \langle Q^{\diamond }(t), u_{0}\rangle \textit{ and } K(t):= \int _{0}^{t} \langle Q^{\diamond }(\sigma ), q(t-\sigma ) \rangle d \sigma . $$

*Then* $v$, *characterised by*

6.9 $$ \int _{0}^{t} v(\tau ) d\tau :=\int _{0}^{t} Z(\sigma ) q(t- \sigma ) d \sigma , $$

*is a solution of* (6.8).

Notice that, since $Z$ is of bounded variation and $Z(0)=0$, then (6.9) amounts to

6.10 $$ v(t)=\int _{0}^{t} Z (d \sigma ) q(t- \sigma ) \quad a.e. $$

### Proof

Using (6.9) and the formula for $Z_{0}$ we can rewrite (6.8) as

6.11 $$\begin{aligned} \int _{0}^{t} Z(\sigma ) q(t-\sigma ) d\sigma &= \int _{0}^{t} Z_{0}( \sigma ) q(t- \sigma ) d\sigma \end{aligned}$$

6.12 $$\begin{aligned} &+ \int _{0}^{t} \int _{0}^{t-\tau } \langle Q^{\diamond }(\sigma ), v( \tau ) \rangle q(t-\tau -\sigma ) d\sigma d\tau . \end{aligned}$$

Notice that

$$\begin{aligned} & \int _{0}^{t} \int _{0}^{t-\tau } \langle Q^{\diamond }(\sigma ), v( \tau ) \rangle q(t-\tau -\sigma ) d\sigma d\tau \\ &= \int _{0}^{t} \int _{\tau }^{t} \langle Q^{\diamond }( r-\tau ), v( \tau ) \rangle q(t-r) dr d\tau \\ & = \int _{0}^{t} \int _{0}^{r} \langle Q^{\diamond }(r-\tau ), \int _{0}^{\tau }q(\tau -s) Z(ds) \rangle q(t-r) d\tau dr, \end{aligned}$$

moreover

$$\begin{aligned} & \int _{0}^{r} \langle Q^{\diamond }(r-\tau ), \int _{0}^{\tau }q(\tau -s) Z(ds) \rangle d\tau \\ &= \int _{0}^{r} \int _{0}^{\tau }\langle Q^{\diamond }(r-\tau ), q(\tau -s) \rangle Z(ds) d\tau \\ &= \int _{0}^{r} \int _{0}^{r-s} \langle Q^{\diamond }(r-s-\sigma ), q( \sigma ) \rangle d\sigma Z(ds) = \int _{0}^{r} K(r-s) Z(ds) \end{aligned}$$

This implies that equation (6.8) can be rewritten as

$$ 0= \int _{0}^{t} \left ( Z(\sigma ) - Z_{0}(\sigma )- \int _{0}^{r} Z(r-s) \mu _{K}(ds) \right ) q(t-\sigma ) d\sigma . $$

Since $Z $ is a solution of this equation the proof is complete. □

## Extensions and Open Problems

First of all we remark that the results presented in Sect. 3 are valid for any model that can be written as (1.2) for a factorisable kernel and not only for the models that have a renewal point. For example, in the simple case where $k(a,\xi ,\omega )$ factorises as the product of $c_{1}(\omega )$ and $c_{2}(a,\xi )$, see for instance Sect. 8.4 and Exercise 9.5 in [22].

The focus of this work has been on the one dimensional reduction, even if the techniques can be adapted to a finite number of renewal points. The assumption on the kernel, in that case, changes into

$$ k(a,\xi , \omega ) = \sum _{i=1}^{n} \int _{[0, a]} \mu _{M_{i}} (ds , \xi ) L_{i}(a-s, \omega ). $$

**Fig. 4:**
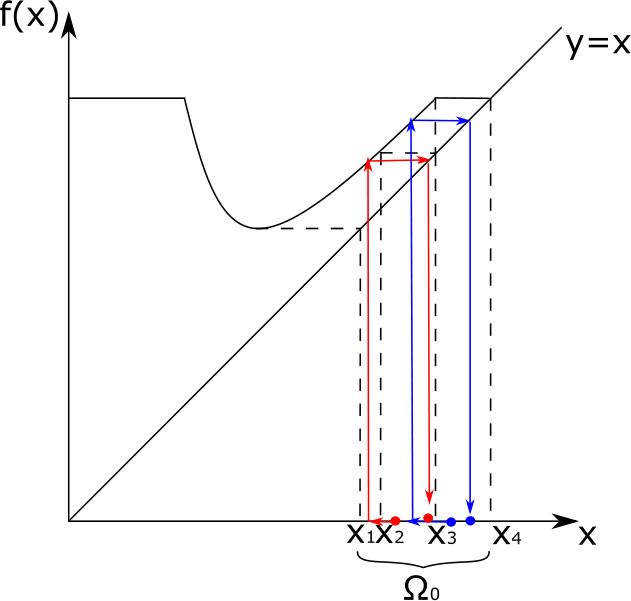
Graph of a boosting function which gives rise to two renewal points $x_{2}$ and $x_{3}$. The blue dot is an individual with immune level at birth in $[x_{3}, x_{4}]$ that crosses the point $x_{3}$ before boosting. The red dot is an individual with immune state at birth in $[x_{2}, x_{3}]$ that crosses $x_{2}$ before boosting (Color figure online)

**Fig. 5:**
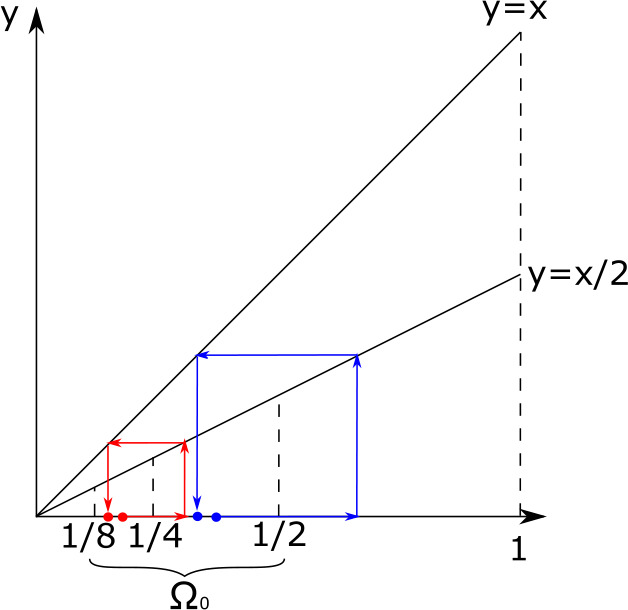
Graph of a cell model with $\Omega =[1/8, 1]$ and such that every cell fragments into two cells of equal size. In red a cell born with size in $[1/8, 1/4]$ that fragments after having crossed $1/4$ and in blue a cell born with size in $[1/4, 1/2]$ that fragments after having crossed $1/2$. The renewal points are $1/4$ and $1/2$ (Color figure online)

The most straightforward interpretation is that in between being born with state $\xi $ and being able to produce offspring with state-at-birth in $\omega $, an individual has to pass through one of $n$ renewal points. But, as indicated in Figs. 4 and 5, the assumption also covers more subtle situations, where individuals with certain states-at-birth are not ‘counted’ themselves and yet the offspring they produce is ‘counted’. From this assumption it should be possible to obtain a system of renewal equations of the form (3.5) and, from the asymptotic behaviour of the solution of that system (see e.g. [47], deduce the one of the solution of (3.1). We stress that the case of finitely many renewal points is relevant for applications, indeed it allows to consider a broader class of models. Two examples of models with two renewal points are indicated in Figs. 4 and 5.

The reduction of the abstract renewal equation works also for time periodic kernels, like the ones considered in [24] and [51], if the kernels are factorisable. Also in this case the asymptotic behaviour found for the reduced equation is expected to determine the behaviour of the solution of the abstract equation, but the details of the “lifting” should be adapted.

It would be interesting to investigate whether the method presented in this work can be adopted for non-biological applications, such as models of growth and fragmentation of aerosols.

A natural extension of this work is the study of the non-linear case as in [19]. In particular, it would be natural to couple equation (3.1) with an equation for the environment, see [53, Eqs. (3.12), (3.13)] or [21, Eq. (2.11)]. The non-linearity arises, in these cases, from the feedback loop between the population and the environment. In the examples of the cell population the environment could represent the food resources as in [21] and [53].

Is the set $\mathscr {X}$ (Definition A.17) isomorphic to a linear subspace of the space of measures on the product $\sigma $-algebra $\mathscr {B}(\mathbb{R}_{+}) \times \mathscr {B}(\Omega _{0})$, so that we can characterise the space in which the solution of the renewal equation (3.1) lives? At the moment we do not have an answer to this question, but it seems possible to apply the Disintegration Theorem to deduce that $\mathscr {X}$ is (up to an isomorphism) a closed subset, hence a linear subspace, of the space of measures endowed with the flat norm. We plan to investigate this in detail in a follow-up paper.

We end with another open problem: can the present results be used to understand the asymptotic behaviour of the solution of equation (3.1), when $k$ is a non factorisable kernel that can be approximated (in a sense to be specified) with factorisable kernels?

## Data Availability

Not applicable.

## Appendix A: Notation, Definitions, and Auxiliary Results

In this appendix we present the notation that we adopt in the paper and the definitions and the lemmas that allow us to prove the existence of a unique solution of the renewal equation (3.1). We follow the approach of [18, 31, 48] and define kernels, the convolution product between kernels and the resolvent.

### A.1 Notation

We specify that $\mathbb{R}_{+}:=[0, \infty ) $ and $\mathbb{R}_{+}^{*}:=(0, \infty )$. We assume $\Omega _{0} \subset \mathbb{R}_{+}^{n}$ to be a Borel set. We assume that $\mathscr {B}(\Omega _{0}):=\{\omega \cap \Omega _{0}: \omega \in \mathscr {B}(\mathbb{R}^{n})\}$ and that $M(\Omega _{0})$ denotes the space of all the signed Borel measures on $\Omega _{0}$, with $M_{+}(\Omega _{0})$ the set of all the non-negative Borel measures on $\Omega _{0}$ and with $M_{+,b}(\Omega _{0})$ the set of all the non-negative bounded Borel measures on $\Omega _{0}$.

$NBV(\mathbb{R}_{+})$ denotes the space of the functions $f$ of bounded variation with $f(0)=0$ and with $f $ right-continuous on the open set $\mathbb{R}_{+}^{*}$ and $NBV_{\mathrm{loc}}(\mathbb{R}_{+})$ denotes the set of the normalised locally bounded variation functions.

$H $ is the Heaviside function, defined as

$$ H(t):= \textstyle\begin{cases} 0\text{ if } t = 0 \\ 1 \text{ if } t > 0. \end{cases} $$

Notice that $H \in NBV(\mathbb{R}_{+})$.

#### Definition A.1

Given a function $G \in NBV_{loc}(\mathbb{R}_{+})$, we say that its distributional derivative is the measure $\mu _{G}$ such that

$$ \mu _{G}([0,t]):=G(t) \quad \forall t \in \mathbb{R}_{+} . $$

This definition is taken from [31, Chap. 3.7]. It is well known that the map $G \mapsto \mu _{G}$ is a one-to-one map from $NBV_{loc}(\mathbb{R}_{+})$ to the space of the finite Borel measures, see for instance [63, Theorem 3.29]. Therefore the measure $\mu _{G}$ is well defined.

#### Remark A.2

If $G \in NBV_{loc}(\mathbb{R}_{+}) $ and the function $f$ is integrable with respect to the measure $\mu _{G}$, then, the integral

$$ \int f(x) \mu _{G}(dx) $$

is called the Lebesgue-Stieltjes integral of $f$ with respect to the function $G$.

When it is well defined, $\hat{f}(\lambda ) $ denotes the Laplace transform of the function $f$,

A.1 $$ \hat{f}(\lambda ) := \int _{0}^{\infty }e^{-\lambda t } f(t) dt, $$

and $\hat{\mu }(\lambda )$ denotes the Laplace transform of a measure

A.2 $$ \hat{\mu }(\lambda ) := \int _{0}^{\infty }e^{-\lambda t } \mu (dt). $$

Since we are interested in the asymptotic behaviour of functions with values in a space of measures, we introduce the notion of total variation and flat norm.

#### Definition A.3

The total variation norm of a measure $\mu \in M(\Omega _{0})$ is defined by

$$ \| \mu \|_{TV} = \sup _{\Pi } \sum _{i=1}^{n} |\mu (\Omega _{0}^{i}) |, $$

where the supremum is taken over all the finite measurable partitions $\Pi :=\{\Omega _{0}^{1}, \dots , \Omega _{0}^{n}\} $ of the set $\Omega _{0}$.

$BL(\Omega _{0})$ denotes the space of the real valued bounded Lipschitz functions, endowed with the norm

$$ \| f \|_{BL}:= \sup _{x \in \Omega _{0}} |f (x)| + \sup _{x \ne y} \frac{|f(x)-f(y)|}{|x-y |}. $$

#### Definition A.4

The flat norm of a measure $\mu \in M(\Omega _{0})$ is defined by

$$ \| \mu \|_{\flat } = \sup \left \{ \left |\int _{\Omega _{0}} f d\mu \right | : f \in BL(\Omega _{0}) \text{ such that } \| f \|_{BL} \leq 1 \right \} . $$

For positive measures $\mu \in M_{+}(\Omega _{0})$

$$ \| \mu \|_{TV} = \| \mu \|_{\flat } = \mu (\Omega _{0}). $$

See [32, Sect. 4, Eq. (4.3] for the above formula and see the appendix of that paper for a nice overview of different characterisations of the flat norm.

For real-valued functions $f$ and $g$ defined on ℝ we use the well-known symbol ∼ to denote asymptotic equivalence: $f \sim g$ as $t \rightarrow \infty $ if

A.3 $$ \lim _{t \rightarrow \infty } \frac{f(t)}{g(t)}=1. $$

For real-valued functions on ${\mathbb{R}} \times \mathscr {B}(\Omega _{0})$ we use the following analogous definition.

#### Definition A.5

Setwise asymptotic equivalence

Let $f$ and $g$ be defined on ${\mathbb{R}} \times \mathscr {B}(\Omega _{0})$. We say that $f(t, \cdot ) \sim g(t,\cdot ) $ *setwise* as $t \rightarrow \infty $ if for every $\omega \in \mathscr {B}(\Omega _{0}) $

A.4 $$ \lim _{t \rightarrow \infty } \frac{f(t,\omega )}{g(t,\omega )}=1. $$

### A.2 Kernel, Convolution Product, Resolvent

The definitions and results presented in this and the following subsection are all variations of the same theme of algebraic nature.

#### Definition A.6

Let $(\mathscr {A}, \ast )$ be an associative algebra and let $k \in \mathscr {A}$. If $r \in \mathscr {A}$ satisfies the equation

$$ r= k + k\ast r = k + r\ast k, $$

then $r$ is called the *resolvent* of $k$.

The definite article is justified by the following proposition.

#### Proposition A.7

*Let* $(\mathscr {A}, \ast )$ *be an associative algebra*. *An element* $k \in \mathscr {A}$ *has at most one resolvent*.

*Let* ℱ *be a left module over* $\mathscr {A}$ *and let* $f \in \mathscr {F}$. *If* $k \in \mathscr {A}$ *has a resolvent* $r \in \mathscr {A}$, *then the equation*

$$ x = f + k\ast x $$

*has a unique solution* $x \in \mathscr {F}$ *and this solution is given by the formula*

$$ x = f + r\ast f. $$

For details we refer to [31, pp. 233-234]

As an application of Proposition A.7 we consider the scalar renewal equation

A.5 $$ X(t) = \int _{0}^{t} X(t-s) \mu _{G}(ds) + y(t), $$

where $G:{\mathbb{R}}_{+}\to {\mathbb{R}}$ is locally of bounded variation and $y:{\mathbb{R}}_{+}\to {\mathbb{R}}$ is locally bounded and measurable. It is easy to show that $G$ has a resolvent $R$, that is the unique solution of

A.6 $$ R(t) = \int _{0}^{t} R(t-s) \mu _{G}(ds) + G(t), $$

and that $R$ is locally of bounded variation. It follows that the unique solution of (A.5) is given by

A.7 $$ X(t) = \int _{0}^{t} \mu _{R}(ds)y(t-s) +y(t). $$

Next we consider more complicated kernels related to the renewal equation (1.2) for unknown measure-valued functions.

#### Definition A.8

Kernel

A kernel is a positive function $k: \mathbb{R}_{+} \times \Omega _{0} \times \mathscr {B}(\Omega _{0}) \rightarrow \mathbb{R}_{+}$ with the following properties

1. for every $(a, \xi ) \in \mathbb{R}_{+} \times \Omega _{0}$, $k(a,\xi , \cdot ) \in M_{+}(\Omega _{0})$ (space of positive Borel measures)
2. for every $\omega \in \mathscr {B}(\Omega _{0})$, the function
  $$ (a,\xi ) \mapsto k(a,\xi ,\omega ), \quad (a,\xi ) \in \mathbb{R_{+}} \times \Omega _{0} $$
  is measurable (with respect to the product Borel $\sigma $-algebra).

Our first aim is to show that the convolution product of two kernels is well defined. It is well known that in the finite dimensional case, (local) integrability of the kernels suffices, see e.g., [31]. Here we shall employ a local boundedness assumption (we do not know whether this assumption is necessary, but we have not been able to proceed without it).

#### Definition A.9

Locally bounded kernel

A locally bounded kernel is a kernel such that for any $T>0$

$$ \sup _{ (a,\xi ) \in [0,T] \times \Omega _{0}} k(a,\xi ,\Omega _{0}) < \infty . $$

#### Lemma A.10

*Let* $k_{1}: \mathbb{R}_{+} \times \Omega _{0} \times \mathscr {B}(\Omega _{0}) \rightarrow \mathbb{R}_{+}$ *and* $k_{2}: \mathbb{R}_{+} \times \Omega _{0} \times \mathscr {B}(\Omega _{0}) \rightarrow \mathbb{R}_{+}$ *be two locally bounded kernels*, *then*, *for every* $a>0$, *the map*

A.8 $$\begin{aligned} s \mapsto \int _{\Omega _{0}} k_{2}(a-s, \xi ,\omega )k_{1}(s , x, d \xi ), \quad a>s \end{aligned}$$

*is measurable and locally bounded*.

#### Proof

Since, for a fixed $\omega \in \mathscr {B}(\Omega _{0})$, the map $(s,\xi ) \mapsto k_{2}(s,\xi ,\omega ) $ is measurable and positive, it can be (pointwise) approximated from below by a non decreasing sequence of simple functions $\varphi _{\omega }^{n}$ [38, Theorem 11.35]. The approximating functions are of the following form

A.9 $$ \varphi _{\omega }^{n}(s,\xi ) :=\sum _{i=1}^{n} \alpha ^{n}_{i}( \omega ) \chi _{A_{i}^{n}(\omega )} (s,\xi ) \quad s \geq 0 \quad \xi \in \Omega _{0} $$

where $A_{i}^{n}(\omega ) \in \mathscr {B}(\mathbb{R}_{+} \times \Omega _{0})$. To prove the measurability of

$$ s \mapsto \int _{\Omega _{0}} \varphi ^{n}_{\omega }(a-s, \xi ) k_{1}(s,x,d \xi ) $$

for a fixed $a >0$, a fixed $\omega \in \mathscr {B}(\Omega _{0})$ and a fixed $x \in \Omega _{0}$, we first prove the measurability of

$$ s \mapsto \int _{\Omega _{0}} \chi _{A}(a-s, \xi ) k_{1}(s,x,d\xi ) ds $$

for every $A \in \mathscr {B}(\mathbb{R}_{+} \times \Omega _{0})$, every $a>0$ and every $x \in \Omega _{0}$. To this end we define the set

$$ \mathscr {D}_{a,x}:=\left \{ A \subset \mathbb{R}_{+} \times \Omega _{0} \text{ s.t. } s \mapsto \int _{\Omega _{0}} \chi _{A}(a-s, \xi ) k_{1}(s,x,d \xi ) \text{ is measurable} \right \} . $$

We show now that $\mathscr {D}_{a,x}$ is a Dynkin system, which is a collection $\mathscr {C} $ of subsets of $\mathbb{R}_{+} \times \Omega _{0}$ such that

1. $\mathbb{R}_{+} \times \Omega _{0} \in \mathscr {C}$;
2. if $A \in \mathscr {C} $, then $A^{c} \in \mathscr {C} $;
3. if $\{ A_{i}\}$ is a sequence of pairwise disjoint sets in $\mathscr {C}$, then $\bigcup _{i=1}^{\infty }A_{i} \in \mathscr {C}$.

Here $A^{c}$ denotes the complement of the set $A$.

Firstly, we show that $\mathbb{R}_{+} \times \Omega _{0} \in \mathscr {D}_{a,x}$. This follows by the fact that

$$ s \mapsto \int _{\Omega _{0}} \chi _{\mathbb{R}_{+} \times \Omega _{0} }(a-s, \xi ) k_{1}(s,x,d\xi ) =k_{1}(s,x,\Omega _{0}) $$

is measurable. If $A \in \mathscr {D}_{a,x}$, then, since $\chi _{A^{c}} = 1- \chi _{A}$ we know that

$$ \int _{\Omega _{0}} \chi _{A^{c}}(a-s, \xi ) k_{1}(s,x,d\xi ) = k_{1}(s,x, \Omega _{0}) - \int _{\Omega _{0}} \chi _{A}(a-s, \xi ) k_{1}(s,x,d \xi ) $$

and by the fact that $\mathbb{R}_{+} \times \Omega _{0} \in \mathscr {D}$ and $A \in \mathscr {D}_{a,x}$ we deduce that $A^{c} \in \mathscr {D}_{a,x}$. Similarly, if $A =\bigcup _{i=1}^{n} A_{i}$ where $\{A_{i}\}$ is a countable family of disjoint sets $A_{i} \in \mathscr {D}$, then from the fact that

$$ \chi _{A }= \sum _{i} \chi _{A_{i}}, $$

it follows that $A \in \mathscr {D}$. We conclude that $\mathscr {D}_{a,x}$ is a Dynkin system. $\mathscr {P}$ denotes the set of all the rectangular subsets of $\mathbb{R}_{+} \times \Omega _{0}$, namely

A.10 $$ \mathscr {P} :=\left \{ A \subset \mathbb{R}_{+} \times \Omega _{0} \text{ s.t. } A=A_{1} \times A_{2} \text{ with } A_{1} \in \mathscr {B}( \mathbb{R}_{+}), \ A_{2} \in \mathscr {B}(\Omega _{0}) \right \} . $$

We first of all notice that $\mathscr {P}$ is a $\pi $-system [62, definition (a), Lemma 1.6]. Indeed, it is non-empty and closed under finite intersections. We aim at proving that $\mathscr {P} \subset \mathscr {D}_{a,x}$. Consider $A \in \mathscr {P} $, since $A=\tilde{A} \times \hat{A}$ with $\tilde{A} \in \mathscr {B}(\mathbb{R}_{+})$ and $\hat{A} \in \mathscr {B}( \Omega _{0})$, we notice that

A.11 $$ \int _{\Omega _{0}} \chi _{A} (a-s,\xi ) k_{1}(s , x, d\xi )= \chi _{ \tilde{A}} (a-s) k_{1}(s , x,\hat{A}). $$

On the other hand, for every $a >0$ and $x \in \Omega _{0}$, the map

$$ s \mapsto \chi _{\tilde{A}} (a-s) k_{1}(s , x,\hat{A}) $$

is measurable because it is the product of the two measurable functions

$$ s \mapsto k_{1}(s , x,\hat{A}), $$

which is measurable by definition, and

$$ s \mapsto \chi _{\tilde{A}}(a-s), $$

which is measurable because $\chi _{\tilde{A}}(a-s)= \chi _{\tilde{A}_{a} }(s)$ where $\tilde{A}_{a}:=\{s \in \mathbb{R}_{*} : s= a-v , \ v \in \tilde{A} \}$ is a Borel set because it is the translation of a Borel set. We conclude that $\mathscr {P} \subset \mathscr {D}_{a,x}$. It follows from [8, Lemma 6.4.2], that

A.12 $$ \mathscr {B}(\mathbb{R}_{+} \times \Omega _{0}) = \mathscr {B}(\mathbb{R}_{+}) \otimes \mathscr {B}(\Omega _{0}) $$

where $\mathscr {B}(\mathbb{R}_{+}) \otimes \mathscr {B}(\Omega _{0})$ denotes the $\sigma $-algebra generated by all the measurable rectangles (sets of the type $R=R_{1} \times R_{2} $ with $R_{1} \in \mathscr {B}(\mathbb{R}_{+})$ and $R_{2} \in \mathscr {B}(\Omega _{0})$). By Dynkin’s Lemma (Lemma A.1.3 in [62]) we deduce that $\mathscr {B} (\mathbb{R}_{+}) \otimes \mathscr {B}( \Omega _{0} ) \subset \mathscr {D}$ and from (A.12) that $\mathscr {B} (\mathbb{R}_{+} \times \Omega _{0} ) \subset \mathscr {D}$. Therefore, for every $A \in \mathscr {B} (\mathbb{R}_{+} \times \Omega _{0} )$, every $a>0$ and every $x \in \Omega _{0}$, the map

$$ s \mapsto \int _{\Omega _{0}} \chi _{A}(a-s, \xi ) k_{1}(s,x,d\xi ) ds $$

is measurable. As a consequence, the map

$$ s \mapsto \int _{\Omega _{0}} \varphi ^{n}_{\omega }(a-s, \xi ) k_{1}(s,x,d \xi ) $$

is measurable for any $a >0$, for any $x \in \Omega _{0}$ and any $\varphi ^{n}_{\omega }$ of the form (A.9) approximating $k_{2}(\cdot , \cdot , \omega )$. By the dominated convergence theorem and by the fact that the limit of measurable functions is a measurable function we can conclude that, for every $\omega \in \mathscr {B} (\Omega _{0})$, the map

$$ s \mapsto \int _{\Omega _{0}} k_{2}(a-s, \xi , \omega ) k_{1}(s,x,d \xi ) $$

is measurable.

The map (A.8) is also locally bounded, indeed for every $a>T>0$

$$\begin{aligned} &\sup _{s \in [0,T]} \int _{\Omega _{0}} k_{2}(a-s, \xi ,\omega )k_{1}(s , x, d\xi ) \\ &\leq \sup _{(s,x) \in [0,T]\times \Omega _{0} } k_{1}(s , x, \Omega _{0}) \sup _{(v,\xi ) \in [a-T,a]\times \Omega _{0} } k_{2}(v , \xi , \Omega _{0}) < \infty . \end{aligned}$$

 □

Next we introduce the definition and the properties of the convolution of two kernels. The definition of convolution that we are going to present is a particular case of the convolution product between kernels presented in [18].

#### Definition A.11

Convolution product of kernels

We define the convolution product of two locally bounded kernels $k_{1}, k_{2}: \mathbb{R}_{+} \times \Omega _{0} \times \mathscr {B}( \Omega _{0}) \rightarrow \mathbb{R}_{+} $ as

A.13 $$ (k_{2} * k_{1})(a, x, \omega ) := \int _{0}^{a}\int _{\Omega _{0}} k_{2}(a-s, \xi ,\omega )k_{1}(s , x, d\xi ) ds. $$

Now we will prove some properties of the convolution that correspond to the properties of [18, Theorem 2.2].

#### Lemma A.12

*The convolution product of two locally bounded kernels is a locally bounded kernel*.

#### Proof

We first of all show that for every $\omega \in \mathscr {B}(\Omega _{0})$, the map

$$ (a,x) \mapsto (k_{2} * k_{1})(a, x, \omega ) $$

is measurable.

As explained in the proof of Lemma A.10, since for a fixed $\omega \in \mathscr {B}(\Omega _{0})$, the map $(s,\xi ) \mapsto k_{2}(s,\xi ,\omega ) $ is measurable and positive, it can be (pointwise) approximated from below by a non decreasing sequence of simple functions $\varphi _{\omega }^{n}$ of the form (A.9).

To prove the measurability of

$$ (a, x) \mapsto \int _{0}^{a} \int _{\Omega _{0}} \varphi ^{n}_{\omega }(a-s, \xi ) k_{1}(s,x,d\xi ) ds $$

we first prove the measurability of

$$ (a, x) \mapsto \int _{0}^{a} \int _{\Omega _{0}} \chi _{A}(a-s, \xi ) k_{1}(s,x,d \xi ) ds $$

for every $A \in \mathscr {B}(\mathbb{R}_{+} \times \Omega _{0})$.

To this end we define the set

$$ \mathscr {D}:=\left \{ A \subset \mathbb{R}_{+} \times \Omega _{0} \text{ s.t. } (a,x) \mapsto \int _{0}^{a} \int _{\Omega _{0}} \chi _{A}(a-s, \xi ) k_{1}(s,x,d\xi ) ds \text{ is measurable} \right \} . $$

We show now that $\mathscr {D} $ is a Dynkin system. Firstly, we show that $\mathbb{R}_{+} \times \Omega _{0} \in \mathscr {D}$. This follows by the fact that

$$ (a,x) \mapsto \int _{0}^{a} k_{1}(s,x,\Omega _{0} ) ds $$

is measurable since it is a Carathéodory function (see [1, Lemma 4.51]). Indeed, once we have fixed $x \in \Omega _{0} $, the map

$$ a \mapsto \int _{0}^{a} k_{1}(s,x,\Omega _{0} ) ds $$

is continuous and the map

$$ x \mapsto \int _{0}^{a} k_{1}(s,x,\Omega _{0} ) ds $$

is measurable thanks to a corollary of Tonelli’s Theorem [8, Corollary 3.4.6]. If $A \in \mathscr {D}$ then, since $\chi _{A^{c}} = 1- \chi _{A}$ we know that

$$ \int _{0}^{a}\! \int _{\Omega _{0}} \chi _{A^{c}}(a-s, \xi ) k_{1}(s,x,d \xi ) ds = \int _{0}^{a} \!k_{1}(s,x,\Omega _{0}) ds - \int _{0}^{a}\! \int _{\Omega _{0}} \chi _{A}(a-s, \xi ) k_{1}(s,x,d\xi ) ds $$

and by the fact that $\mathbb{R}_{+} \times \Omega _{0} \in \mathscr {D}$ and $A \in \mathscr {D}$ we deduce that $A^{c} \in \mathscr {D}$. Similarly, if $A =\bigcup _{i=1}^{n} A_{i}$ where $\{A_{i}\}$ is a countable family of disjoint sets $A_{i} \in \mathscr {D}$, then from the fact that

$$ \chi _{A }= \sum _{i} \chi _{A_{i}}, $$

it follows that $A \in \mathscr {D}$. We conclude that $\mathscr {D}$ is a Dynkin system.

Let $\mathscr {P}$ be defined as (A.10), we aim at proving that $\mathscr {P} \subset \mathscr {D}$. Consider $A \in \mathscr {P} $, with $A=\tilde{A} \times \hat{A}$ with $\tilde{A} \in \mathscr {B}(\mathbb{R}_{+})$ and $\hat{A} \in \mathscr {B}( \Omega _{0})$. Thanks to (A.11), we have that for every fixed $x \in \Omega _{0}$ the map

A.14 $$ a \mapsto \int _{0}^{a} \int _{\Omega _{0}} \chi _{A}(a-s, \xi ) k_{1}(s,x,d \xi ) ds = \int _{0}^{a} \chi _{\tilde{A}} (a-s) k_{1}(s , x,\hat{A}) ds $$

is continuous thanks to the fact that the convolution of bounded functions is continuous [31, Chap. 2, Theorem 2.2]. On the other hand, the map

$$ (s,x) \mapsto \chi _{\tilde{A}} (a-s) k_{1}(s , x,\hat{A}) $$

is measurable because it is the product of the two measurable functions

$$ (s,x) \mapsto k_{1}(s , x,\hat{A}), $$

which is measurable by definition, and

$$ (s,x) \mapsto \chi _{\tilde{A}}(a-s), $$

which is measurable because it is a constant in $x$ and $\chi _{\tilde{A}}(a-s)= \chi _{\tilde{A}_{a} }(s)$ where $\tilde{A}_{a}:=\{s \in \mathbb{R}_{*} : s= a-v , \ v \in \tilde{A} \}$ is a Borel set because is the translation of a Borel set. As a consequence, the map

A.15 $$ x \mapsto \int _{0}^{a} \int _{\Omega _{0}} \chi _{A}(a-s, \xi ) k_{1}(s,x,d \xi ) ds = \int _{0}^{a} \chi _{\tilde{A}} (a-s) k_{1}(s , x,\hat{A}) ds $$

is measurable thanks to [8, Corollary 3.4.6]. Combining the measurability of (A.15) and the continuity of (A.14), we conclude that if $A \in \mathscr {P}$, then

$$ (a,x) \mapsto \int _{0}^{a} \int _{\Omega _{0}} \chi _{A}(a-s, \xi ) k_{1}(s,x,d \xi ) ds $$

is a Carathéodory function and is therefore measurable. We conclude that $\mathscr {P} \subset \mathscr {D}$.

Dynkin’s Lemma and equality (A.12) imply that for every $A \in \mathscr {B} (\mathbb{R}_{+} \times \Omega _{0} )$ the map

$$ (a, x) \mapsto \int _{0}^{a} \int _{\Omega _{0}} \chi _{A}(a-s, \xi ) k_{1}(s,x,d \xi ) ds $$

is measurable.

As a consequence, the map

$$ (a, x) \mapsto \int _{0}^{a} \int _{\Omega _{0}} \varphi ^{n}_{\omega }(a-s, \xi ) k_{1}(s,x,d\xi ) ds $$

is measurable for any $\varphi ^{n}_{\omega }$ of the form (A.9) approximating $k_{2}(\cdot , \cdot , \omega )$.

By the dominated convergence theorem and by the fact that the limit of measurable functions is a measurable function we can conclude that, for every $\omega \in \mathscr {B} (\Omega _{0})$, the map

$$ (a,x) \mapsto (k_{2} * k_{1})(a, x, \omega ) $$

is measurable.

For every $\omega \in \mathscr {B}(\Omega _{0})$

$$\begin{aligned} \sup _{(a,x) \in [0,T] \times \Omega _{0}} (k_{2}* k_{1} )(a,x, \omega )& \leq \sup _{(s, \xi ) \in [0,T] \times \Omega _{0} } k_{2}(s, \xi , \omega ) \sup _{x \in \Omega _{0}} \int _{0}^{T} k_{1}(s, x, \Omega _{0} ) ds < \infty . \end{aligned}$$

Let us check that, for every $(a, \xi ) \in \mathbb{R}_{+}\times \Omega _{0}$ the map $(k_{2} * k_{1})(a, \xi , \cdot ) $ is a measure. Clearly

$$ (k_{2} * k_{1})(a, \xi , \emptyset )= \int _{0}^{a}\int _{\Omega _{0}} k_{2}(a-s, \xi ,\emptyset )k_{1}(s , x, d\xi ) ds=0, $$

for every $\omega \in \mathscr {B}(\Omega _{0}) $

$$ (k_{2} * k_{1})(a, \xi , \omega ) \geq 0 $$

and for every $(a, \xi ) \in \mathbb{R}_{+} \times \Omega _{0} $

$$ (k_{2} * k_{1})(a, \xi , \Omega _{0}) < \infty . $$

Let us check that $(k_{2} * k_{1})(a, \xi , \cdot )$ is countably additive. Consider a countable collection of disjoint sets $\{ E_{i} \}_{i=1}^{\infty }$ in $\mathscr {B}(\Omega _{0}) $, then, by the monotone convergence theorem we have that

$$\begin{aligned} (k_{2} * k_{1})(a, \xi , \bigcup _{i=1}^{\infty }E_{i} ) &= \int _{0}^{a} \int _{\Omega _{0}} k_{2}(a-s, \xi , \bigcup _{i=1}^{\infty }E_{i} )k_{1}(s , x, d\xi ) ds \\ &= \int _{0}^{a}\int _{\Omega _{0}} \sum _{i=1}^{\infty }k_{2}(a-s, \xi , E_{i} )k_{1}(s , x, d\xi ) ds \\ &= \sum _{i=1}^{\infty }\int _{0}^{a}\int _{\Omega _{0}} k_{2}(a-s, \xi , E_{i} )k_{1}(s , x, d\xi ) ds \\ &= \sum _{i=1}^{\infty }\int _{0}^{a}\int _{\Omega _{0}} (k_{2} * k_{1})(a, \xi , E_{i} ). \end{aligned}$$

 □

#### Remark A.13

As already observed above, if we assume that the set $\Omega _{0}$ is finite, and in particular that $\Omega _{0} := \{ \xi _{1}, \xi _{2}, \dots , \xi _{n}\}$, then we can omit the boundedness assumption in Definition A.11. Indeed, in this case, it is easy to see that the convolution product of kernels is well defined. In this case

$$\begin{aligned} & \int _{0}^{a}\int _{\Omega _{0}} k_{2}(a-s, \xi ,\xi _{m})k_{1}(s , \xi _{j}, d\xi ) ds= \int _{0}^{a} \sum _{i} k_{2}^{i,m}(a-s)k^{j,i}_{1}(s) ds \\ & \leq \sum _{i} \int _{0}^{a} k_{2}^{i,m}(a-s)k^{j,i}_{1}(s) ds=(k_{2} * k_{1} )_{m,j}(a), \quad 1\leq m,j \leq n, \end{aligned}$$

where $k^{i,j}(a)=k(a, \xi _{j}, \xi _{i})$. Since the convolution product of $L^{1}$ “functions” is an $L^{1} $ “function” (see for instance [31]), we can conclude that $k_{2} * k_{1}$ is well defined almost everywhere.

When $\Omega _{0}$ is not finite it is sufficient to assume the boundedness of the kernel as in Definition A.22 to be able to conclude that the convolution product is well defined. We do not know if this assumption is necessary, but we have not been able to relax it.

Let $h: \Omega _{0} \rightarrow \mathbb{R}_{+}$ be integrable with respect to the measure $(k_{2} * k_{1})(a, x , \cdot )$ and consider the integral

A.16 $$ \int _{\Omega _{0}} h (\xi ) (k_{2} * k_{1})(a, x , d\xi ) \quad (a,x ) \in \mathbb{R}_{+} \times \Omega _{0}. $$

If we substitute in the integral the expression for $k_{2} * k_{1}$ we formally obtain that

$$ \int _{\Omega _{0}} h(\xi ) (k_{2} * k_{1})(a, x, d\xi ) = \int _{ \Omega _{0}} \int _{0}^{a} \int _{\Omega _{0}} k_{1}(s ,x , d\eta ) ds h(\xi ) k_{2}(a-s, \eta , d\xi ) . $$

The right hand side of the above equation is not well defined, as the meaning of the expression $\int _{\Omega _{0}} k_{2}(a-s, \eta , d\xi )k_{1}(s ,x , d\eta ) $ is not clear. In the following lemma, we show that this difficulty is only a notational difficulty and, using the definition of integral with respect to a measure, we find a concrete expression for (A.16).

#### Lemma A.14

*Assume* $k_{1}, k_{2} : \mathbb{R}_{+} \times \Omega _{0} \times \mathscr {B}( \Omega _{0}) \rightarrow \mathbb{R}_{+}$ *are two locally bounded kernels*. *Let* $h : \Omega _{0} \rightarrow \mathbb{R}$ *be a bounded and measurable function*, *then*

A.17 $$ \int _{\Omega _{0}} h(\xi ) (k_{2} * k_{1})(a, x, d\xi ) = \int _{0}^{a} \left (\int _{\Omega _{0}} \left ( \int _{\Omega _{0}} h(\xi ) k_{2}(a-s, \eta , d\xi ) \right ) k_{1}(s ,x , d\eta ) \right ) ds. $$

#### Proof

We first of all show that (A.17) is true for step functions. Let

$$ h_{n}(\xi ) := \sum _{i=1}^{n} \alpha _{i} \chi _{A_{i}}(\xi ) \quad \alpha _{i} \in \mathbb{R}_{+}, \quad A_{i} \in \mathscr {B}(\Omega _{0}) $$

then

$$\begin{aligned} & \int _{\Omega _{0}} h_{n}(\xi ) (k_{2} * k_{1})(a, x, d\xi ) = \int _{\Omega _{0}} \sum _{i=1}^{n} \alpha _{i} \chi _{A_{i}}(\xi ) (k_{2} * k_{1})(a, x, d\xi ) \\ &= \sum _{i=1}^{n} \alpha _{i} (k_{2} * k_{1})(a, x, A_{i}) =\sum _{i=1}^{n} \alpha _{i} \int _{0}^{a}\int _{\Omega _{0}} k_{2}(a-s, \eta ,A_{i})k_{1}(s , x, d\eta ) ds \\ &=\int _{0}^{a} \left ( \int _{\Omega _{0}} \left ( \int _{\Omega _{0}} \sum _{i=1}^{n} \alpha _{i} \chi _{A_{i}}(\xi ) k_{2}(a-s, \eta ,d \xi ) \right ) k_{1}(s , x, d\eta ) \right ) ds \\ &=\int _{0}^{a} \left ( \int _{\Omega _{0}} \left ( \int _{\Omega _{0}} h_{n}(\xi ) k_{2}(a-s, \eta ,d \xi ) \right ) k_{1}(s , x, d\eta ) \right ) ds. \end{aligned}$$

Next assume that $h$ is a non negative measurable function. By the definition of integral with respect to a measure we have that

$$ \int _{\Omega _{0}} h(\xi ) (k_{2} * k_{1})(a, x, d\xi )= \lim _{n \rightarrow \infty } \int _{\Omega _{0}} s_{n}(\xi )(k_{2} * k_{1})(a, x, d\xi ) $$

where $s_{n}$ is a sequence of step functions that approximate $h$. Since (A.17) holds for step functions we conclude that it holds for any non negative measurable function by passing to the limit.

If $h$ is allowed to take negative values, we can argue in the classical way, writing $h=h^{+} - h^{-}$ and applying the above result for the positive functions $h^{+} $ and $h^{-}$. □

#### Lemma A.15

*The product* ∗ *is associative and distributive over* +. *Let* $\{k_{n}\}_{n}$ *be an increasing sequence of locally bounded kernels pointwise converging to a locally bounded kernel* $k$, *and let* $l$ *be a locally bounded kernel*, *then*

A.18 $$ \lim _{n \rightarrow \infty } l * k_{n} = l * k. $$

#### Proof

Let us check, using Fubini-Tonelli theorem that $(k_{3} *k_{2} )* k_{1} = k_{3}* (k_{2} * k_{1} ) $:

$$\begin{aligned} & ((k_{3} *k_{2} )* k_{1} )) (a, \xi , \omega ) \\ &= \int _{0}^{a} \left ( \int _{\Omega _{0}} k_{1}(r,\xi , d\eta ) \int _{0}^{a-r} \left ( \int _{\Omega _{0}} k_{2}(s,\eta , dx) k_{3}(a-r-s,x, \omega ) \right ) ds \right ) dr \\ & = \int _{0}^{a} \int _{0}^{a-r} \left ( \int _{\Omega _{0}} \left ( \int _{\Omega _{0}} k_{3}(a-r-s,x, \omega ) k_{2}(s,\eta , dx) \right ) k_{1}(r,\xi , d\eta )\right ) dsdr \\ &= \int _{0}^{a} \int _{r}^{a} \left ( \int _{\Omega _{0}}\left ( \int _{\Omega _{0}} k_{3}(a-v,x, \omega ) k_{2}(v-r,\eta , dx) \right ) k_{1}(r,\xi , d\eta ) \right ) dv dr \\ &= \int _{0}^{a} \int _{0}^{v} \left ( \int _{\Omega _{0}}\left ( \int _{\Omega _{0}} k_{2}(v-r,\eta , dx) k_{3}(a-v,x, \omega ) \right ) k_{1}(r,\xi , d\eta ) \right ) dr dv \\ &= ( k_{3} * (k_{2} * k_{1} ) )(a, \xi , \omega ) \end{aligned}$$

Notice that for the last equality we have used equation (A.17). In a similar way, it is possible to check that $(k_{1} + k_{2} ) * k_{3} = k_{1} * k_{3} + k_{2} * k_{3} $.

The last property follows from the monotone convergence theorem. □

Let us introduce the following notation,

$$ k^{*1} :=k $$

and

$$ k^{*(n+1)} :=k * k^{*n} \text{ for } n \geq 1. $$

#### Lemma A.16

Resolvent

*Let* $k$ *be a locally bounded kernel*. *For every* $t >0 $, $x \in \Omega _{0}$, $\omega \in \mathscr {B}(\Omega _{0})$, *the series*

$$ r(t, x, \omega ):= \sum _{n=1}^{\infty }k^{* n}(t, x , \omega ), $$

*converges in* $\overline{\mathbb{R}_{+}}$. *In fact*, $r$ *is a locally bounded kernel and it is the only solution of the resolvent equation*

$$ r = k + r * k = k + k * r. $$

We say that $r$ is the *resolvent of* $k$.

#### Proof

First of all we notice that the resolvent equation has a unique solution. Indeed, suppose there exists another solution $Q$, then

$$\begin{aligned} r &=k + r *k= k +r * (Q - k * Q) = k+ r* Q - r * k * Q= k + (r-r * k) * Q \\ &=k + k * Q = Q. \end{aligned}$$

The convergence of

$$ r(t, x, \omega ):= \sum _{n=1}^{\infty }k^{* n}(t, x , \omega ), $$

in $\overline{\mathbb{R}_{+}}$ follows from the positivity of $k$.

The map $(t,x) \mapsto r(t, x, \omega ) $ is measurable because it is the pointwise limit of measurable functions.

Notice also that for any $\omega \in \mathscr {B}(\Omega _{0})$ and for any $T>0$

$$\begin{aligned} \sup _{(t,\xi ) \in [0,T] \times \Omega _{0} } r(t, \xi , \omega ) &\leq \sum _{n=1}^{\infty }\sup _{(t,x) \in [0,T] \times \Omega _{0} } k^{* n}(t, x , \omega ) \\ &\leq \sup _{(t,\xi ) \in [0,T] \times \Omega _{0} } k(t, \xi , \omega ) \sum _{n=2}^{\infty }\sup _{x \in \Omega _{0}} \int _{0}^{T} k^{*(n-1) }(a, x , \Omega _{0})da \\ & \leq \sup _{(t,\xi ) \in [0,T] \times \Omega _{0} } k(t, \xi , \Omega _{0}) \sum _{n=2}^{\infty }\left ( \sup _{x \in \Omega _{0}} \int _{0}^{T} k(a, x , \Omega _{0})da \right )^{n-1}. \end{aligned}$$

Where, for the last inequality we have used the associativity property of the convolution product. From the above computations we conclude that, if we assume that

$$ \sup _{x \in \Omega _{0}} \int _{0}^{T} k(a, x , \Omega _{0})da < 1, $$

then $r$ is a locally bounded kernel. Moreover, thanks to (A.18) we deduce that $r_{T}$, the restriction of $r$ over $[0,T]$, solves the resolvent equation

$$ r = k + r * k = k + k * r $$

for every $t \in [0,T]$.

If, instead

$$ \sup _{ x \in \Omega _{0}} \int _{0}^{T} k(a, x , \Omega _{0}) da \geq 1 $$

we can adapt the above argument. Since $k(\cdot ,\cdot , \Omega _{0})$ is locally bounded, there exists a $\lambda >0$ such that

$$ \sup _{x \in \Omega _{0}} \int _{0}^{T} e^{- \lambda a} k(a, x , \Omega _{0})da < 1. $$

Applying the above argument to $k_{\lambda }(a,x, \omega ) := e^{- \lambda a} k(a, x , \omega )$ we deduce that $r(a,x,\omega )=e^{\lambda a} r_{\lambda }(a,x, \omega )$ is a locally bounded kernel and its restriction on $[0,T]$ is the only solution of the resolvent equation, for the kernel $k$, on $[0,T]$.

We can repeat the above argument for every interval of finite length and, thanks to the uniqueness of the solution of the resolvent equation we obtain a global solutions by gluing the local solutions. □

### A.3 Existence, Uniqueness and Representation of the Solution of the Renewal Equation

#### Definition A.17

$\mathscr {X}$ denotes the set of functions $f: \mathbb{R}_{+} \times \mathscr {B}(\Omega _{0}) \rightarrow \mathbb{R}_{+} $ such that for every $a \in \mathbb{R}_{+}$, $f(a, \cdot )$ is a measure, the function $f(\cdot , \omega )$ is measurable for every $\omega \in \mathscr {B}(\Omega _{0})$ and $f(\cdot , \Omega _{0})$ is locally integrable.

The set $\mathscr {X}$ can be seen as a subset of the space of the measures on the product $\sigma $-algebra $\mathscr {B} (\mathbb{R}_{+}) \times \mathscr {B}(\Omega _{0}) $ that have a density in the first variable. A better characterization of this set, as well as a possible topological structure on it, are not, at the moment, available. These might be subject of future work.

#### Lemma A.18

*Every locally bounded kernel* $k: \mathbb{R}_{+} \times \Omega _{0} \times \mathscr {B}(\Omega _{0}) \rightarrow \mathbb{R}_{+}$ *induces a linear operator* $\mathscr {L}_{k}: \mathscr {X} \rightarrow \mathscr {X} $ *defined as*

$$ \mathscr {L}_{k}( f )(t, \omega ) := \int _{0}^{t} \int _{\Omega _{0}} k(t- \sigma , x, \omega ) f(\sigma , dx)d\sigma \quad t >0 \quad \omega \in \mathscr {B}(\Omega _{0}). $$

#### Proof

Let us first show that, if $f\in \mathscr {X}$, then $\mathscr {L}_{k}( f ) \in \mathscr {X}$. The fact that for every $t \in \mathbb{R}_{+}$ the map $\mathscr {L}_{k}( f )(t, \cdot )$ is a measure in the second variable follows as in Lemma A.12. The measurability of $\mathscr {L}_{k} (f)(\cdot , \omega )$ can be proven using the measurability of $f$ as in the proof of Lemma A.12. Notice that, applying Fubini-Tonelli theorem we deduce that for every $\omega \in \mathscr {B}(\Omega _{0})$

$$\begin{aligned} \int _{0}^{T} \mathscr {L}_{k} (f)(t, \omega ) dt &= \int _{0}^{T} \int _{0}^{t} \int _{\Omega _{0}} k(t-\sigma , x, \omega ) d\sigma f( \sigma , dx) dt \\ & = \int _{0}^{T} \int _{\sigma }^{T} \int _{\Omega _{0}} k(t-\sigma , x, \omega ) f(\sigma , dx)dt d\sigma \\ & \leq \int _{0}^{T} \int _{\Omega _{0}} \int _{0}^{T} k(\tau , x, \omega ) d\tau f(\sigma , dx) d\sigma \\ & \leq \int _{0}^{T} f(\sigma , \Omega _{0}) d\sigma \sup _{ x \in \Omega _{0}} \int _{0}^{T} k(\tau , x, \Omega _{0}) d\tau < \infty . \end{aligned}$$

The linearity of the operator $\mathscr {L}_{k} $ can be checked via a direct computation. □

#### Remark A.19

Notice that, if $k $ is a locally bounded kernel, then, for every $\xi \in \Omega _{0}$, the map $(a, \omega ) \mapsto k(a, \xi , \omega )$ belongs to the set $\mathscr {X}$. Therefore, the convolution of an arbitrary kernel $k_{1}$ with a given kernel $k_{2}$ can be seen as the application of the linear operator $\mathscr {L}_{ k_{2}} $ to $k_{1}(\cdot , \xi , \cdot )$

#### Lemma A.20

*If* $k_{1}, k_{2}: \mathbb{R}_{+} \times \Omega _{0} \times \mathscr {B}( \Omega _{0}) \rightarrow \mathbb{R}_{+}$ *are locally bounded kernels*, *then*

$$ \mathscr {L}_{k_{2}} \mathscr {L}_{k_{1}} = \mathscr {L}_{ k_{2} * k_{1}} $$

#### Proof

Let us show that for any $f \in \mathscr {X}$, it holds that $\mathscr {L}_{k_{2}} (\mathscr {L}_{ k_{1}} (f))=\mathscr {L}_{ k_{2} * k_{1}}( f) $.

$$\begin{aligned} ( \mathscr {L}_{k_{2} * k_{1}} (f ) )(t, \omega )&= \int _{0}^{t} \int _{ \Omega _{0}} (k_{2} * k_{1} )(t-\sigma , x, \omega ) f(\sigma , dx) d \sigma \\ &= \int _{0}^{t} \int _{\Omega _{0}}^{(x)} \int _{0}^{t-\sigma } \int _{ \Omega _{0}}^{(\xi )} k_{2}(s, \xi , \omega ) k_{1}(t-\sigma -s, x, d \xi ) f(\sigma , dx) ds d\sigma \\ &= \int _{0}^{t} \int _{0}^{t-s} \int _{\Omega _{0}}^{(x)} \int _{ \Omega _{0}}^{(\xi )} k_{2}(s, \xi , \omega ) k_{1}(t-\sigma -s, x, d \xi ) f(\sigma , dx) d\sigma ds \\ &=\mathscr {L}_{k_{2}}( \mathscr {L}_{k_{1}} f (t, \omega )), \end{aligned}$$

where in the last equality we have used (A.17) with $h$ given by $\xi \mapsto k_{2}(s, \xi , \omega )$ and where we have applied Fubini-Tonelli theorem. □

The following theorem is a special case of [18, Theorem 2.3].

#### Theorem A.21

*Let* $f_{0} \in \mathscr {X}$ *and let* $k$ *be a locally bounded kernel*. *The unique solution of the renewal equation*:

A.19 $$\begin{aligned} b = \mathscr {L}_{k} b + f_{0} \end{aligned}$$

*is the function* $b \in \mathscr {X}$ *given by*

A.20 $$ b : = f_{0} + \mathscr {L}_{r} f_{0} $$

*where* $r$ *is the resolvent of* $k$.

#### Proof

Let $b$ be given by

$$ b(t, \omega ) = f_{0}(t, \omega ) +( \mathscr {L}_{r} f_{0}) (t, \omega ), $$

it follows that $b(t, \omega )$ is a measure in the second variable and $b(\cdot , \omega ) $ is locally integrable because $\mathscr {L}_{r} f_{0}$ is locally integrable and $f_{0}(\cdot , \omega )$ is integrable.

The function $b $ solves equation (3.1), indeed

$$\begin{aligned} \mathscr {L}_{k} b + f_{0} &= \mathscr {L}_{k}( \mathscr {L}_{r} f_{0}+ f_{0}) + f_{0}= \mathscr {L}_{k * r} f_{0}+ \mathscr {L}_{k} f_{0} + f_{0} = \mathscr {L}_{ k * r+ k} f_{0}+ f_{0} \\ &= \mathscr {L}_{r} f_{0}+f_{0}=b. \end{aligned}$$

Notice that every solution of equation (3.1) is of the above form, indeed, let $b$ be a solution of (3.1), then

$$\begin{aligned} f_{0}= b-\mathscr {L}_{k} b = b-\mathscr {L}_{ r-r * k} b = b - \mathscr {L}_{r} b + \mathscr {L}_{ r * k } b = b - \mathscr {L}_{ r} (b - \mathscr {L}_{k} b ) =b -\mathscr {L}_{r} f_{0}.\qquad \end{aligned}$$

 □

### A.4 Laplace Kernel, Next Generation Operator, Basic Reproduction Number, Malthusian Parameter

Following the terminology in [18] we define the Laplace kernels as follows.

#### Definition A.22

Laplace kernel

A Laplace kernel is a kernel such that there exists $z_{0} \leq 0$ with

$$ \sup _{\xi \in \Omega _{0} } \int _{0}^{\infty }e^{-z_{0} a} k(a,\xi , \Omega _{0}) da < \infty . $$

By the definition of Laplace kernel, for every $\Re \lambda > z_{0} $, with $z_{0} \leq 0$, the Laplace transform of $k $ is well defined,

$$ \hat{k}(\lambda , x, \omega ):=\int _{0}^{\infty }e^{- \lambda a }k(a, x, \omega ) da < \infty \quad\ \text{and}\ \quad \hat{k}(0, x, \omega )= \int _{0}^{\infty }k(a, x, \omega ) da < \infty . $$

#### Definition A.23

Let $k $ be a Laplace kernel with $z_{0} \leq 0$. The operator $\mathbb{K}: M_{b}^{+}(\Omega _{0}) \rightarrow M_{b}^{+}(\Omega _{0}) $ defined as

A.21 $$\begin{aligned} (\mathbb{K} \varphi )(\omega )=\int _{\Omega _{0}} \varphi (d\xi ) \int _{0}^{\infty }k(a,\xi , \omega ) da \quad \omega \in \mathscr {B}( \Omega _{0}), \end{aligned}$$

is the *next generation operator*.

#### Definition A.24

The *basic reproduction number* $R_{0} $ is the spectral radius of the next generation operator $\mathbb{K}$.

#### Definition A.25

If the eigenproblem

A.22 $$ \nu (\omega )=\int _{\Omega _{0}} \hat{k}(r, x, \omega ) \nu (dx ) $$

has a unique solution $(r, \nu ) \in \mathbb{R} \times M_{+}(\Omega _{0})$, then $r$ is called *Malthusian parameter*.

To prove the existence of $R_{0}$, of the eigencouple $(r, \nu )$ and to prove the classical relation

$$ {\mathrm{sign}} (R_{0} -1 )=\mathrm{sign} (r) $$

additional assumptions on the kernel $k $ are needed (see for instance [56, 57]. In the main text of the present paper we elaborate the details under a factorisation assumption on the kernel $k$.

## Appendix B: The PDE Formulation of Structured Population Models

Historically, models of cell growth and division were first formulated as PDEs with non-local terms [5, 49, 60]. It was observed by Bell [6] that there is a close relation between the PDE and a Volterra integral equation (renewal equation). This was later elaborated and exploited to gain information about the asymptotic behaviour (balanced exponential growth) in, for instance, [16, 33, 36].

In the case of cells reproducing by fission into equal parts, the PDE for the size density $n$ takes the form

B.1 $$ \partial _{t} n(t, x) + \partial _{x} (g(x) n(t, x)) =- \left ( \mu (x)+ \gamma (x) \right ) n(t, x) + 4 \gamma (2x) n(t, 2x) , $$

with $0< x<1$ and where the term with argument $2x$ should be interpreted as zero when $2x \geq 1$. Below we consider a more general version of (B.1) allowing for unequal division.

Our results imply that the (weak) solutions of (B.1) converge, under the non-degeneracy condition $g(2x)<2g(x)$, to a stable distribution if $R_{0}=1$, while they exhibit balanced exponential growth or decline if $R_{0}>1$ or $R_{0}<1$, respectively. We plan to explain in detail how our results allow to deduce the asymptotic behaviour of $n$ in a follow-up paper.

Next we briefly discuss the relation between the PDE and renewal equation formulations. Assume, for the moment, that the initial condition $\Phi (\cdot , \cdot ) $ is absolutely continuous with respect to the Lebesgue measure on $\mathbb{R}^{2}$ and denote its density with $\psi (\cdot , \cdot )$.

Let $n(t,x)$ be the density of cells with size $x$ at time $t$. The interpretation of $n$ suggests that it should be defined as follows: If $x \in \Omega _{M}:=[\overline{x}, 1)$, then

B.2 $$ n(t,x):= \textstyle\begin{cases} n_{0}(X(-t, x)) \hat{\mathscr {F}} (X(-t,x), x) \frac{g(X(-t,x))}{g(x)} &\text{ if } t < T(x) \\ \frac{\hat{\mathscr {F}}( \overline{x} , x )}{g(x)} \tilde{b}(t-T( x)) &\text{ if } t \geq T(x) \end{cases} $$

with

$$ n_{0}(x)=\int _{\Omega _{0}}\frac{ \hat{\mathscr {F}}(\xi , x)}{g(x)} \psi (-\tau (\xi , x), \xi ) d\xi , \quad x \in \Omega $$

and $\tilde{b} $ the representative of $B'$, which solves the differentiated version of (3.5) (i.e. (5.13) Sect. 5). If instead $x \in \Omega _{0}$, then

B.3 $$ n(t,x):= \textstyle\begin{cases} n_{0}(X(-t, x)) \hat{\mathscr {F}} (X(-t,x), x) \frac{g(X(-t,x))}{g(x)} & \\ +\int _{\Omega _{M}} \int _{\{ \xi \in \Omega _{0}: t> \tau (\xi ,x) \}} \hat{\mathscr {F}}(z,x) \frac{\gamma (y)}{g(x)} \eta (y,dz) \cdot &\\ \cdot n(t-\tau (z,x), y) dy &\text{ if } t < T(x ) \\ \int _{\Omega _{M}} \int _{\Omega _{0}} \hat{\mathscr {F}}(z,x) \frac{\gamma (y)}{g(x)} \eta (y,dz) n(t-\tau (z,x), y) dy &\text{ if } t \geq T(x). \end{cases} $$

It is possible to prove that the function $n$ given by the formulas (B.2) and (B.3) is the solution, in the sense of distributions, of

B.4 $$\begin{aligned} \partial _{t} n(t, x) + \partial _{x} (g(x) n(t, x)) = & - \left ( \mu (x)+ \gamma (x) \right ) n(t, x) \\ &+ \partial _{x} \int _{\Omega }\gamma (y) \eta (y, (0, x ]) n(t, y) dy. \end{aligned}$$

We postpone the proof to a follow-up paper.

Notice that in case $\eta (x, \omega ):= 2 \delta _{x/2 }(\omega ) $ we have that

$$\begin{aligned} \partial _{x} \int _{\Omega }\gamma (y) \eta (y, (0, x ]) n(t, y) dy= 2 \partial _{x} \int _{2\inf \Omega }^{2x} \gamma (y) n(t, y) dy= 4 \gamma (2x) n(t, 2x) \end{aligned}$$

and the equation (B.4) reduces to (B.1).

### Proposition B.1

*Let* $\mu $, $g$, $\eta $ *and* $\gamma $ *be as in Assumption* 4.1*and*, *moreover*, *let* $\gamma $ *and* $g$ *be as in case* 2 *of Assumption* 4.2. *Let one of the two assumptions* 1 *or* 2 *of Proposition* 4.4*hold and let us adopt the notation of the Proposition*. *If* $R_{0}$ *given by* (4.7) *is equal to* 1, *then the solution* $n$ *of* (B.4) *satisfies*

$$\begin{aligned} \left \| \frac{ n(t,\cdot ) }{D}- \chi _{\Omega _{M}}(\cdot ) \frac{\hat{\mathscr {F}}(\overline{x}, \cdot )}{g(\cdot )} - \chi _{ \Omega _{0}}(\cdot ) \int _{\Omega _{0}} \int _{\Omega _{M}} \hat{\mathscr {F}}(z,\cdot ) \gamma (y) \eta (y,dz) \frac{\hat{\mathscr {F}}( \overline{x} , y)}{g(y)} dy \right \| _{L^{1}( \Omega )} \rightarrow 0 \end{aligned}$$

*as time goes to infinity*.

We postpone the proof of this and similar results for $R_{0} \neq 1$ to a future work.

Let us now return to the general case in which $\Phi $ is a Radon measure. We first define the bounded variation function $N(t,x)$ representing the number of cells of size in $(0,x]$ at time $t$. As we already pointed out in the introduction, measures and functions giving *numbers* of individuals in a set are conceptually easier than densities.

If $x \in \Omega _{0}$, then

B.5 $$\begin{aligned} N(t,x)=&\int _{\Omega _{0}} \int _{t-\tau (\xi , x) }^{0} \mathscr {F}(t-s, \xi ) \Phi (d\xi , ds) \\ &+ \int _{\Omega _{0}} \int _{\Omega _{M}} \int ^{t}_{t-\tau (z,x)} \mathscr {F}(t-s, z) \gamma (y) \eta (y,dz) n(s,y) dy ds \end{aligned}$$

and if $x \in \Omega _{M}$

B.6 $$\begin{aligned} N(t,x)=&\int _{\Omega _{0}} \int _{t-\tau (\xi , x) }^{0} \mathscr {F}(t-s, \xi ) \Phi (d\xi , ds) + \int _{t-\tau (\overline{x}, x)}^{t} \mathscr {F}(t-s, \overline{x}) \tilde{b}(s) ds \\ &+ \int _{\Omega _{0}} \int _{\Omega _{M}} \int ^{t}_{t-\tau (z, \overline{x})} \mathscr {F}(t-s, z) \gamma (y) \eta (y,dz) n(s,y) dy ds \end{aligned}$$

The function $N$ satisfies the integrated version of (B.4):

$$\begin{aligned} \partial _{t} N(t, x)=& - g(x) \partial _{x} N(t,x) - \int _{(0, x] } ( \mu (y) +\gamma (y) ) N (t, dy) \\ &+ \int _{\Omega }\gamma (y) \eta (y,(0,x]) N(t, dy) \end{aligned}$$

and therefore the asymptotic behaviour of $N$ can be analysed in a similar way.

As for the model of waning and boosting we can, if $\Phi $ is absolutely continuous with respect to Lebesgue measure, write down an expression for the density $n(t,x)$ of individuals with immune level $x$ at time $t$ as a function of $\tilde{b}$ as we did for the cell fission model.

If the boosting function $f$ is such that $f^{-1}$ takes only finitely many values, one can formulate a PDE for the density, like in [17], where $f^{-1}$ is double valued. When $f^{-1}$ can take a continuum of values one needs to consider measures/NBV functions, and work with the number $N(t,x)$ of individuals with immunity level in $(0,x]$ at time $t$. We postpone the elaboration of this to a follow up paper.
